# Genome-Wide Transcriptional Analysis of Genes Associated with Acute Desiccation Stress in *Anopheles gambiae*

**DOI:** 10.1371/journal.pone.0026011

**Published:** 2011-10-04

**Authors:** Mei-Hui Wang, Osvaldo Marinotti, Anne Vardo-Zalik, Rajni Boparai, Guiyun Yan

**Affiliations:** 1 Program in Public Health, University of California Irvine, Irvine, California, United States of America; 2 Department of Molecular Biology and Biochemistry, University of California Irvine, Irvine, California, United States of America; 3 Department of Biology, Pennsylvania State University, York, Pennsylvania, United States of America; University of North Carolina at Charlotte, United States of America

## Abstract

Malaria transmission in sub-Saharan Africa varies seasonally in intensity. Outbreaks of malaria occur after the beginning of the rainy season, whereas, during the dry season, reports of the disease are less frequent. *Anopheles gambiae* mosquitoes, the main malaria vector, are observed all year long but their densities are low during the dry season that generally lasts several months. Aestivation, seasonal migration, and local adaptation have been suggested as mechanisms that enable mosquito populations to persist through the dry season. Studies of chromosomal inversions have shown that inversions 2La, 2Rb, 2Rc, 2Rd, and 2Ru are associated with various physiological changes that confer aridity resistance. However, little is known about how phenotypic plasticity responds to seasonally dry conditions. This study examined the effects of desiccation stress on transcriptional regulation in *An. gambiae*. We exposed female *An. gambiae* G3 mosquitoes to acute desiccation and conducted a genome-wide analysis of their transcriptomes using the Affymetrix *Plasmodium/Anopheles* Genome Array. The transcription of 248 genes (1.7% of all transcripts) was significantly affected in all experimental conditions, including 96 with increased expression and 152 with decreased expression. In general, the data indicate a reduction in the metabolic rate of mosquitoes exposed to desiccation. Transcripts accumulated at higher levels during desiccation are associated with oxygen radical detoxification, DNA repair and stress responses. The proportion of transcripts within 2La and 2Rs (2Rb, 2Rc, 2Rd, and 2Ru) (67/248, or 27%) is similar to the percentage of transcripts located within these inversions (31%). These data may be useful in efforts to elucidate the role of chromosomal inversions in aridity tolerance. The scope of application of the anopheline genome demonstrates that examining transcriptional activity in relation to genotypic adaptations greatly expands the number of candidate regions involved in the desiccation response in this important malaria vector.

## Introduction

Malaria is an acute infectious disease caused by *Plasmodia* parasites, which are transmitted by the female *Anopheles* mosquitoes. *Anopheles gambiae* is responsible for approximately 80% of malaria transmission in sub-Saharan Africa [1]. In this region of Africa, mosquito vector abundance and malaria transmission intensity fluctuate between distinct annual dry and rainy seasons [2]-[6]. Depending on the specific locality, dry seasons may last from one month to several months, during which *An. gambiae* density is very low. However, mosquito populations increase rapidly following the onset of the rainy season [7], [8], and malaria cases peak a few weeks after the rainy season begins [9]. Vector control measures aimed at reducing mosquito dry-season populations have, therefore, been proposed as a means of reducing vector abundance at the onset of the rainy season [10].

The mechanisms associated with mosquito survival during the dry season and the rapid malaria vector population build up at the onset of the rainy season are an ongoing subject of investigation. Caged *An. gambiae* mosquitoes, maintained with daily access to human blood and sucrose solution, survived on average 34 days during the dry season in western Kenya [11]. This longevity is remarkably shorter than the duration of the dry season in most parts of sub-Saharan Africa. Several hypotheses have been proposed regarding mechanisms by which mosquito populations survive through the dry season and maintain population growth in the rainy season. For example, mosquitoes may disperse from surrounding refugia, where larval breeding sites persist throughout the dry season or the whole year [12]. However, mosquito dispersal is limited, and the normal flight range of *An. gambiae* is generally no more than 1 km [13], [14]. Therefore, mosquito dispersal is an important mechanism for population persistence only when the area is adjacent to long-lasting breeding sites.

Another theory suggests that, during dry periods, mosquitoes undergo aestivation, a dormant state extending longevity. However, reports of *An. gambiae* aestivation in the field are rare. For example, Charlwood et al. [15] conducted extensive sampling of *An. funestus*, *An. gambiae*, and *An. arabiensis* in a 300 square km area of the dry savannah zone of Tanzania, and found no substantiated evidence of aestivation. We know of only two reports of anopheline mosquito aestivation in the field. In the arid areas about 20 km from the Nile Valley in the Khartoum region of Sudan, *An. arabiensis* females exhibited reduced blood feeding activity during the dry season, but ovarian development was retarded and the females were unable to fully develop eggs [16], [17]. In Sahelian villages of Mali, mark-release-recapture studies involving a total of 2,397 males and 4,534 females of *An. gambiae s.l*. mosquitoes identified one female that survived for 7 months over the whole dry season [18]. While these data are suggestive of aestivation, whether the observed changes in feeding behavior, reproductive physiology, and longevity were the results of genetic regulatory mechanisms responding to arid environmental conditions remains unclear. Laboratory studies showed that mosquitoes can adjust their physiology to deal with stress resulting from exposure to low humidity in the environment [18]. For example, *An. arabiensis* displays significantly higher desiccation resistance than *An. gambiae*. This difference is associated with body water content prior to desiccation, and is independent of water loss during desiccation, metabolic rate, and respiratory pattern [19].

Chromosomal polymorphisms, such as inversions, have been observed in populations that connect to harsh environmental conditions [20]. Inversion can reduce recombination along inverted chromosomal segments, functioning as adaptive gene complexes that promote the buildup of favorable combinations of alleles within inversions [21], [22]. Research over the past two decades has identified in *An. gambiae* chromosomal inversions and inversion combinations (2La, 2Rb, 2Rc, 2Rd and 2Ru) that are significantly correlated with adaptations to aridity/humidity conditions [20], [23], [24]. Polymorphic inversions tend to cluster on chromosomal arms 2R and 2L, but not on X, 3R, and 3L [25]. Inversion 2La is physically located between positions 20.52–42.16 Mb on the left arm of chromosome 2, and inversions 2Rb, 2Rc, 2Rd, and 2Ru are located on the right arm at 18.50–26.74 Mb, 26.78–31.45 Mb, 31.37–43.84 Mb and 31.48–35.50 Mb regions, respectively [20], [23]. The effects of such chromosomal inversions may include desiccation resistance and may be modulated by mosquito age or environmental conditions. For example, while the effect became obscured with age, *An. gambiae* mosquitoes with the 2La inversion displayed increased resistance to desiccation [26]. Although previous studies support the role of natural selection in maintaining polymorphic inversions, whether these inversions alter gene structure [27] or do they modify expression attributes [28], [29] under conditions of desiccation stress remains unclear. Allelic differentiation between the chromosomal rearrangements may also contribute to local adaptation [30].

We examined genome-wide transcriptional responses to desiccation in *An. gambiae* G3 mosquitoes (MR4, http://www.mr4.org/). *An. gambiae* G3 are polymorphic for 2La and 2Rbc [31], and are thus appropriate for a study of putative desiccation-responsive genes within the associated inversions. To explicitly screen for genes responding to desiccation, mosquitoes were exposed to periods of low (30%) and high (70%) relative humidity (RH). Desiccation exposure periods were chosen based on a stress survival curve. A total of 248 genes displayed altered transcript accumulation in response to desiccation stress. Although the range of humidity used in this study may not reflect natural humidity ranges in the field, gene expression under these experimental conditions does, nonetheless, shed light on *Anopheline* mosquito response to desiccation at the molecular level. Our results suggest a correlation between 2La and 2Rs and aridity tolerance. Substantial numbers of other genes not carried on inversions also responded to desiccation, demonstrating the importance of understanding the effect of aridity in relation to adaptive phenotypes.

## Results

### Mosquito survival under different humidity conditions

**Figure 1:**
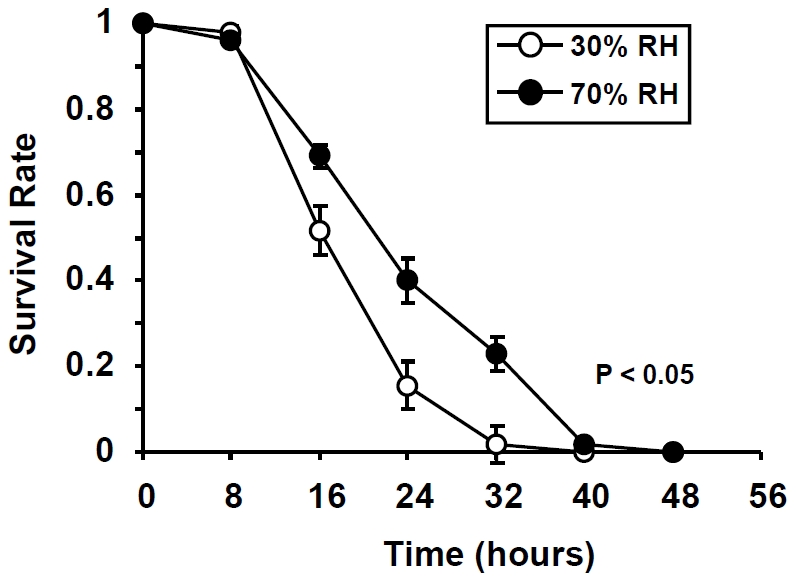
Survival of *An. gambiae* mosquitoes under desiccation stress. Four-day old female mosquitoes were exposed to 70% (black circle) or 30% (white circle) relative humidity without access to water or sugar. The number of dead mosquitoes was recorded every 8 hours until all died. Data represent means ± SEM for n = 10 independent replicates.

*An. gambiae* mosquitoes exhibited a significantly longer average lifespan when deprived of a water source and kept at 70% RH, than those that were deprived of water at 30% RH (Fig. 1) (Wilcoxon test, χ^2^ = 5.56, P<0.05). After 16 h of exposure to desiccation, 48% of the mosquitoes maintained at 30% RH died, whereas only 31% in the 70% RH treatment group died. At 32 h of desiccation stress, 95% and 77% of mosquitoes had died in the 30% and 70% RH groups, respectively. The average length of survivorship was 26.2±1.8 hrs for mosquitoes exposed to 70% RH, and 15.6±1.1 hrs for those maintained at 30% RH.

### Desiccation-responsive gene profiling

**Figure 2:**
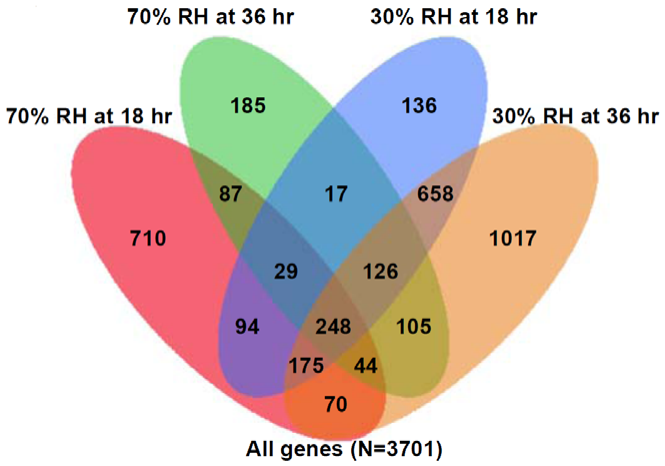
Venn diagram illustrating the distribution of transcripts significantly regulated in response to desiccation in each of the four experimental conditions analyzed in this study (*P*<0.001). The transcriptional profiles of desiccated mosquitoes were compared to control profiles (from four-day old mosquitoes not exposed to desiccation).

Out of 16,941 *An. gambiae* probe sets investigated with the Affymetrix GeneChip *Anopheles*/*Plasmodium* array (http://media.affymetrix.com/support/technical/datasheets/plasmodium_datasheet.pdf), 3,701 probes (21.8%) were found to be significantly (*P*<0.001) regulated in at least one of the four comparisons, 18 h or 36 h post-exposure time points versus 0 h time points for each of the two desiccation treatments (Fig. 2). A total of 1,460 (8.6%) and 844 (5.0%) probe sets revealed significant changes in the accumulation of the corresponding transcripts after 18 h and 36 h under 70% RH, respectively. Exposure at 30% RH for 18 h and 36 h resulted in 1,483 (8.8%) and 2,443 (14.4%) probes showing significant changes in transcription, respectively.

**Figure 3:**
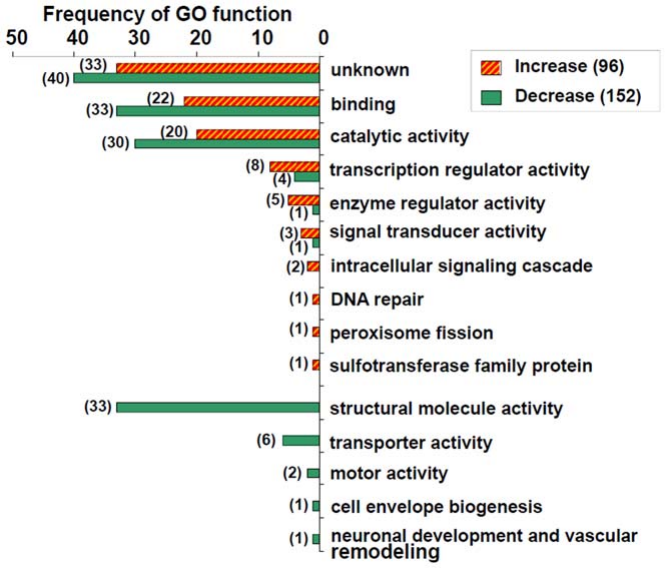
Function classification of desiccation-responsive genes in *Anopheles gambiae*. Gene annotations were derived from gene ontology. The detailed list of the desiccation-responsive genes is in Table S1.

A total of 248 (1.5%) transcripts were found to consistently exhibit significantly altered transcript accumulation in all four comparisons (Table S1), which we thus describe as “desiccation-responsive” genes. Following desiccation, 96 of these desiccation responsive genes showed increased transcriptional accumulation, and 152 showed decreased transcription. Based on their putative functions, these genes were categorized as encoding proteins associated with protein or ion binding, catalytic activity, enzyme regulator activity, signal transduction activity, structural proteins, transcription regulation, transporter activity, and motor activity (Fig. 3). Transcripts showing increased levels of accumulation code for proteins that function in intracellular signaling cascades (2 transcripts), DNA repair (1), peroxisome fission (1), and sulfotransferase activity (1). In contrast, transcripts that code for proteins with transporter activity (6), motor activity (2), cell envelope biogenesis (1), neuronal development and vascular remodeling (1) displayed decreased transcriptional activity, as did transcripts that code for structural proteins (33).

### Transcriptional activity varied according to length of desiccation period

**Figure 4:**
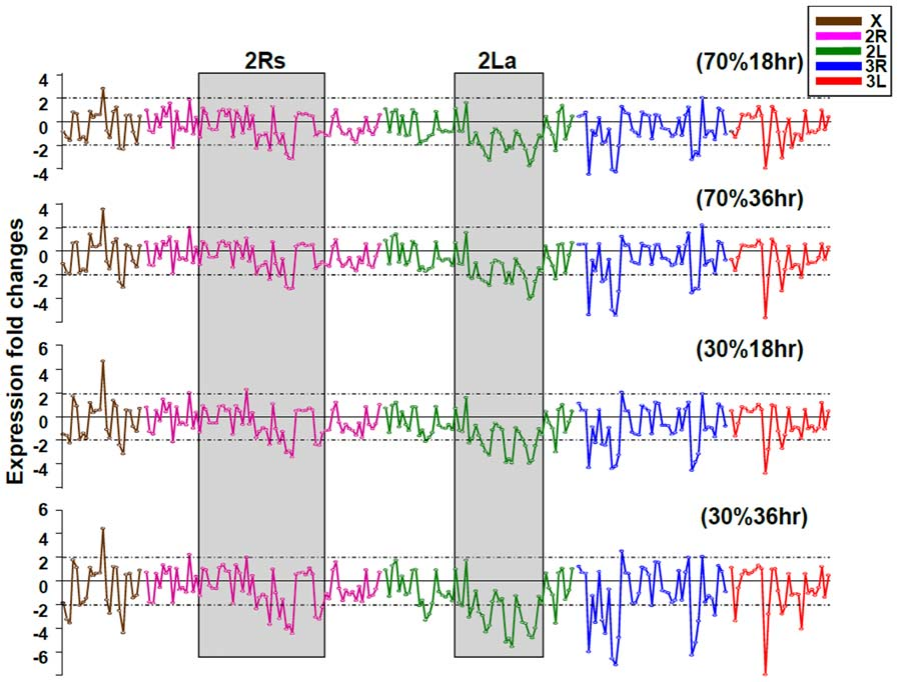
Changes in transcriptional profiles for the desiccation-responsive genes in two treatments (30% and 70% relative humidity) at 18 and 36 hours in relation to the control (0 h, no stress exposure). The transcripts are arranged according to their chromosomal locations in each of the chromosomes X, 2R, 2L, 3R, and 3L.

**Figure 5:**
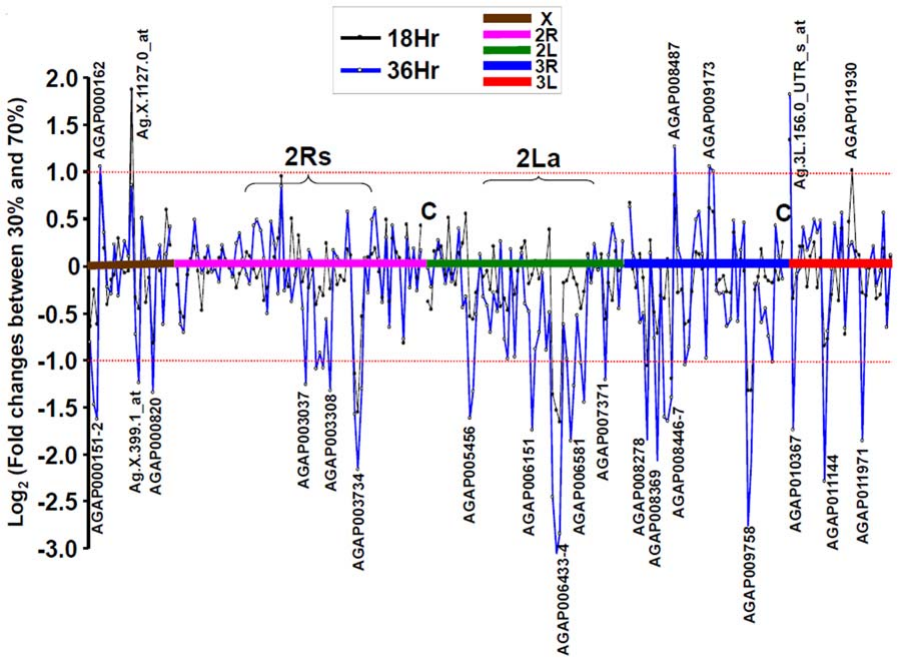
Changes in transcriptional profiles on a logarithm **scale between** ***Anopheles gambiae*** **mosquitoes exposed to 30% relative humidity in comparison to 70% relative humidity at 18 and 36 hours.** The transcripts are arranged according to their chromosomal locations (X, 2R, 2L, 3R, and 3L). “C” represents the centromere. The transcript identity was labeled for those genes exhibiting more than a 2-fold difference in mRNA production (dashed lines).

The observed changes in transcriptional levels of the desiccation-responsive genes are shown in Fig. 4. The genes are presented according to their chromosomal locations. Transcription profiles of desiccation-responsive genes correlated with reduction in RH (70% RH to 30% RH) and exposure time (18 h to 36 h). Figure 5 illustrates the changes in the transcript accumulation of desiccation-responsive genes (n = 248) between 30% RH and 70% RH at 18 h or 36 h of desiccation exposures. The threshold value for a significant change in transcription was 2 (Fig. 4) or 1 on a logarithmic scale (Fig. 5).

The genes exhibiting the largest reduction in transcript accumulation show putative functions in chitin metabolism (AGAP000820, AGAP003261, AGAP003308, AGAP005456, AGAP006433, AGAP006434, AGAP006829, AGAP008446-7 and AGAP009758), lipid transports (AGAP008369), nucleotide catabolic process (AGAP011971), response to oxidative stress (AGAP003714), and binding function (AGAP003037, AGAP003734, AGAP006581, AGAP006586, AGAP008278 and AGAP008450) (Fig. 5). Genes showing the largest increase in transcript accumulation have functions in biosynthesis of amino acids (AGAP000162), sphingomyelin phosphodiesterase activity (AGAP008487), and phosphoric ester hydrolase activity (AGAP009173) (Fig. 5).

### Desiccation-induced transcriptional responses of genes in relation to chromosomal inversions

In the Affymetrix *Plasmodium/Anopheles* Genome Array, approximately 31% (5,342/16,941) of probe sets are located within 2La and 2Rs (2Rb, 2Rc, 2Rd, and 2Ru). Only 27% (67/248) of the identified desiccation-responsive genes were mapped within these inversion areas (χ^2^ = 1.29, *P*>0.10) (see detailed gene list in Table S2). Thus, the hypothesis that a higher proportion of genes in the 2Rs and 2La chromosomal inversions exhibited a significant transcriptional response to desiccation stress was not supported by our experimental data. Genes located outside of the 2La and 2Rs inversions boundaries or at other chromosomes (X, 3R and 3L) also exhibited increased or decreased transcriptional activity following desiccation.

Among the 248 desiccation-responsive genes, 67 genes located within the 2La and 2Rs inversion regions responded to desiccation stress (Table S2). Thirty were located in 2La inversions, 10 in 2Rb, 8 in 2Rc, 4 in 2Ru, and 19 in 2Rd (four genes located in the 2Ru region overlap with 2Rd). Among these genes, 72% (48/67) exhibited decreased levels of transcript accumulation in response to a low-humidity environment, and 28% (19/67) showed increased transcriptional activity under the same conditions. The majority of the genes (27/30) in the 2La inversion displayed decreased levels of transcript accumulation under desiccation conditions, whereas about half (21/37) of the genes in 2Rs inversions exhibited similar decreases.

### qRT-PCR validation of microarray results

**Figure 6:**
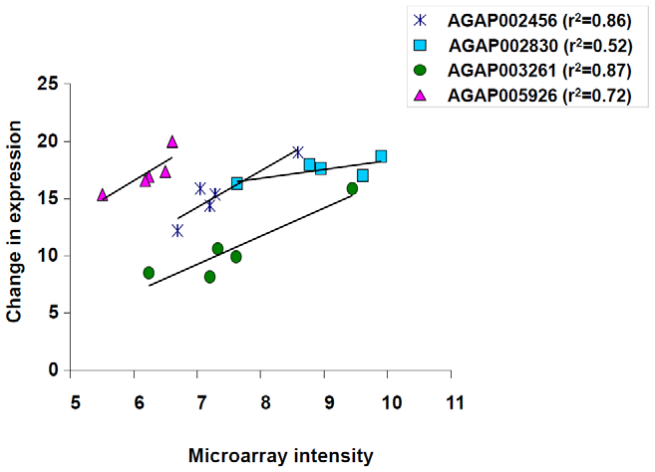
Quantitative reverse-transcript (qRT-PCR) validation of differential transcription of four genes originally identified from microarray analysis. The transcription levels of the four candidate genes were measured in triplicate by qRT-PCR for each desiccation exposure and non-exposure group. The X axis is characterized as the intensity of gene expression from the microarray, and the Y axis represents the fold increase/decrease in transcription from qRT-PCR experiments.

To confirm the changes in the transcriptional activity observed for genes AGAP002456, AGAP003261, AGAP002830, and AGAP005926 in the microarray experiments, qRT-PCR was used to determine relative levels of mRNA accumulation. The qRT-PCR analysis was conducted using mosquito RNA samples independent of those samples used in microarray experiments (Table 1). Results of qRT-PCR experiments were consistent with the microarray data (Fig. 6), with both methods showing down-regulation of AGAP002456 and AGAP003261 and up-regulation of AGAP002830 and AGAP005926 in mosquitoes following desiccation.

**Table 1:** List of the genes and primers used for the qRT-PCR assay.

Gene	Regulation by desiccation	Location	Forward (5′ -3′)	Reverse (5′ -3′)	Size
AGAP002830	↑	2Rc	CGTTCCACTACGCAGGATG	GAGTGGCATCTGCACGATAA	90 bp
AGAP005926	↑	2La	CAAAAGAAGCTGAAACGACGA	CGCTGCTCCTTTTGATCTTT	92 bp
AGAP002456	↓	2Rb	ACGAAGTGAGCAACCATGC	TTTCCGCTTGCACCTGTAAT	97 bp
AGAP003261	↓	2Ru	ACCACAGCCTTCAACCACTT	GGTAGCACCGAGGAGCAG	95 bp

“↑” increased expression in response to desiccation, and “↓” decreased expression in response to desiccation.

## Discussion

This study demonstrates the effects of desiccation stress on transcriptional regulation in the malaria vector, *An. gambiae*. In order to survive desiccation, insects adjust gene expression to achieve physiological plasticity, including discontinuous gas exchange [32], changes in cuticle lipid composition [33], decreased of metabolic rates, and the suppression of reproductive and developmental processes [34]. In an effort to gather more information on mechanisms involved in desiccation responses in *An. gambiae*, we conducted a genome-wide survey of the transcriptional profiles of desiccated and non-desiccated mosquitoes, and identified 248 desiccation-responsive genes.

In *An. gambiae*, chromosomal inversions or a combination of inversions are correlated with aridity/humidity conditions [20], [23], [24]. One aim of this study was to determine whether desiccation-responsive genes were located within 2La or 2Rs inversions. This Affymetrix array covered approximately 16,941 *A. gambiae* transcripts, 31% of which were located within the inversion areas (2La and 2Rs). Twenty-seven percent (67/248) of transcripts affected by desiccation were located in these regions, indicating that genes located outside the 2La or 2Rs inversions are also affected by desiccation. Interestingly, a block of genes in inversion 2La (22 genes, from AGAP005998 to AGAP006964) consistently exhibited decreased transcript accumulation in response to desiccation. In inversions 2Rc and 2Rd, one block of genes exhibited increased levels of transcription (6 genes, AGAP003397 to AGAP003652), while another block displayed decreased levels of mRNA accumulation (6 genes, AGAP002936 to AGAP3308) (see Table S2). Genes within an inversion are in linkage disequilibrium, segregate as a single locus, and are inherited together as a “supergene” [35]. Our data show that these genes also may be co-regulated. Possibly blocks of genes within inversions 2La, 2Rc, and 2Rd share regulatory elements and transcription factors, causing consistent increased or decreased transcription in response to desiccation stress.

One important function of the cuticle is to protect insects from losing water. The cuticle is formed mainly by proteins and chitin and is covered with a layer of epicuticular lipids and waxes. Cuticle proteins (CP) include non-structural and structural proteins [36]. The largest CP family in arthropods is the CPR family, which is characterized by a conserved motif, the R&R Consensus, first identified by Rebers and Riddiford (1988) [37]. In *Drosophila melanogaster*, CPR gene expression was found to be regulated by extrinsic factors, such as the circadian clock [38], and in pea aphids by photoperiod changes [39]–[41]. We identified 33 *An. gambiae* desiccation-responsive genes coding for cuticle structural proteins, all exhibiting reduced transcript accumulation when mosquitoes were exposed to the desiccation treatment. It is possible that the decreased CPR gene expression reflects the impact of a dry environment upon the epidermal cells located immediately underneath the cuticle. CPR proteins are synthesized by the epidermal cells of the insect integument. In our experiments mosquitoes suffered an abrupt change in environmental humidity conditions. It would be interesting to examine whether natural mosquito populations that acclimate to gradual changes in humidity exhibit similar changes in CPR gene transcription.

We also identified 50 desiccation responsive genes with known metabolism-related functions. The metabolic functions of virtually all organisms are altered in response to dehydration. For example, the metabolic rate of female *Culex pipiens* mosquitoes is reduced in response to desiccation [42]. In a addition to reduced metabolism, mosquitoes may respond to unfavorable climatic factors by reducing flight activity. This behavioral change is reflected by the reduced transcription of motor activity genes, flightin (AGAP004877) and myosin (AGAP007249), observed in our study could have been the result of a corresponding reduction in metabolism. A reduction in transcription of genes encoding muscle components was previously observed in *An. gambiae* gravid females, which also display reduced flight activity [43].

Oxidative damage can be one of the most deleterious effects of water depletion [44]. Desiccation stress increases the formation of reactive oxygen species (ROS) resulting in nucleic acid damage with severe consequences on overall metabolism [45]. A positive association between DNA repair efficiency and thermo-tolerance has been demonstrated in *D. melanogaster*. Thermo-tolerant flies tend to repair DNA more efficiently after heat stress [46]. A similar phenomenon has been observed in plant seeds and desiccation-resistant bacteria in which expression in genes involving DNA repair increased after dehydration [47]–[49]. AGAP004261 and AGAP005926 displayed increase transcription in response to desiccation and are associated with DNA repair functions. AGAP005926 codes for a protein with forkhead-associated domain that plays an important role in the DNA-damage response [50]. In plants, forkhead-associated domain mutants are more sensitive to osmotic stress and drought than wild types [51]. Peroxisomes are single-membrane organelles derived from the endoplasmic reticulum, and are replicated by fission [52]. The interior of the peroxisome is the sight of a diverse range of biochemical reactions, including the detoxification of hydrogen peroxide (H_2_O_2_), one major contributor to oxidative damage [53]. Since desiccation stress increases the production of ROS, peroxisome activity may be adjusted to minimize the deleterious effects of molecules such as H_2_O_2_. We identified AGAP007812, a gene associated with peroxisome fission, which is accumulated at higher levels during desiccation.

Increased expression of heat shock proteins (HSP) during dehydration has been reported in *An. gambiae*, *Ae. aegypti* and *Culex pipiens* [54] as well as other organisms such as nematodes and bacteria [55], [56]. RNAi directed against HSP70 or HSP90 in *Ae. aegypti* results in a reduction in dehydration tolerance [54]. Increased transcription of AGAP002386, which codes for a DnaJ (Hsp40) homolog, was observed in this study.

We observed that the number of genes exhibiting significant changes in expression is contingent on the duration of desiccation stress. For example, a total of 1,483 genes exhibited significant change at 18 h after exposure to 30% relative humidity, including those encoding proteins with functions associated with DNA replication, protein folding, and the cell cycle. In contrast, after exposure to the same low humidity condition for 36 h, nearly twice the number of genes (2,443) showed significant changes in transcription levels. A significant number of these genes are involved in apoptosis, oxidation reduction, signal transduction, biosynthetic processes, and cell redox homeostasis, which are notably involved in stress responses [57], [58].

In summary, as a step toward a better understanding of mosquitoes' response to dry conditions, we used a DNA microarray to examine transcriptional responses when *An. gambiae* mosquitoes received acute exposure to a low-humidity environment. Mosquito *An. gambiae* is observed to have strong correlation between chromosomal inversions and degree of aridity [59]. This study provided a deeper understanding of how these genes located in the inversions 2La and 2Rs and their alleles respond to desiccation stress. Genetic variations in the 2La and 2Rs inversions should be further investigated to determine if different genes within these inversions respond similarly to stress in different *An. gambiae* strains and natural populations. This may explain the persistence of inversions in natural populations and reveal favorable combinations of alleles that act together to facilitate adaptation to diverse environments. Although our experiment did not gradually introduce the low humidity conditions, as would occur naturally in the field, the desiccation-responsive genes identified in this study provide a set of candidate genes for further examination of genetic mechanisms of physiological responses to desiccation stress for this important malaria vector.

## Materials and Methods

### Effects of desiccation on mosquito survivorship

Eggs of *Anopheles gambiae* G3 strain were received from MR4 (http://www.mr4.org/) and hatched immediately upon arrival. Larvae were reared under standard conditions [60]. Adults were maintained at 27°C, 70% relative humidity (RH) on a 12:12 light cycle with access to water and sugar ad libitum. At four days post-eclosion, groups of 10 adult females were transferred to 600 ml mosquito rearing cups, and subsequently exposed to 70% RH (low desiccation) or 30% RH (high desiccation) without access to sugar or water. HOBO data loggers (Onset Computer Corporation, Pocasset, MA) were placed inside each humidity incubator to monitor temperature and RH. The number of dead mosquitoes was recorded every 8 hours until no mosquitoes survived. Five replicates were used for each humidity condition, and two independent trials were conducted. Kaplan-Meier survival analysis was used to determine the statistical significance between the two trials within one humidity treatment, and the survivorship data were pooled for the two trials, as no significant difference was detected.

### Microarray experiment design

Approximately 250 female mosquitoes (4–5 days old) were transferred to a 3.8 liter mosquito cage, and were subsequently exposed to 70% or 30% RH without access to sugar or water. A total of 4 replicate cages were used simultaneously for each humidity condition. Twelve live mosquitoes were collected from each cage prior to desiccation (t = 0 h). Twelve survivors were collected at 18 h and again at 36 h post-exposure. Specimens were collected for microarray analysis of only nine survivors for t = 36 h at 30% RH, as it yielded fewer surviving mosquitoes. All samples were preserved in RNAlater® (Ambion, Inc.), and placed in a −80°C freezer until RNA extraction. Total RNA from three whole-mosquito bodies was extracted and used as one biological sample for microarray analysis. Four biological samples were prepared for each treatment. The entire microarray experiment used a total of 19 biological samples, consisting of 4 for t = 0 h, 4 for t = 18 h at 70% RH, 4 for t = 36 h at 70% RH, 4 for t = 18 h at 30% RH, and 3 for t = 36 h at 30% RH.

### RNA isolation, amplification, labeling, and array hybridizations

A total of 19 Affymetrix GeneChip® *Plasmodium*/*Anopheles* genome arrays were used. The arrays included probe sets representing approximately 16,941 *An. gambiae* transcripts, each set consisting of 11 pairs of 25-bp oligonucleotide. Total RNA was isolated from frozen samples using TRIzol Reagent (Gibco BRL Life Technologies, Rockville, MD). The details of amplification, labeling, and hybridization have been previously described [60]. RNA processing and hybridization were performed at the DNA and Protein MicroArray Facility at the University of California, Irvine.

### Microarray data analysis

The measured probe intensities were analyzed with Expression Console ver.1.1 software (Affymetrix, Inc.) using PLIER (probe logarithmic intensity error) algorithm default values to produce a summary value of the probe sets (Quantification Scale: Linear; Quantification Type: Signal and Detection P-Value; Background: PM-GCBG; Normalization Method: Sketch-Quantile). A JMP Genomics software package (SAS Institute Inc., Cary, NC) was then used to identify desiccation-responsive genes. We first performed the analysis of variance (ANOVA) with a false discovery rate of 0.05, and used the method described by Benjamini and Hochberg [61] to correct for multiple comparisons. The analysis involved a total of four comparisons of gene expression value,18 h vs. 0 h and 36 h vs. 0 h for each of the two treatments (70% and 30% RH). Genes were defined as displaying significantly altered transcriptional activity using a cutoff threshold value of *P*<0.001. Genes showing significant changes in transcription levels in the four comparisons were identified by Venn Diagram analysis [62]. The probes for transcripts produced from genes located in inversions 2La and 2Rs were determined by White et al. [20] . To examine whether probes in chromosome inversions showed consistently increased or decreased levels of transcription, we plotted the logarithm of the difference in probe hybridization intensities between 30% and 70% RH against the probes' locations along the chromosomes for all 248 desiccation-responsive genes using 2 as the threshold value for defining significance.

### Data deposition

All data sets have been deposited in the Gene Expression Omnibus, http://www.ncbi.nlm.nih.gov/geo/(accession nos. GSE25433 and GSM624288-624306). All data are MIAME-compliant.

### Microarray result validation with quantitative reverse-transcription PCR (qRT-PCR)

Four genes exhibiting significant changes in levels of transcription in response to desiccation stress were selected for qRT-PCR analysis based on chromosomal location and magnitude of difference of altered transcriptional activity in order to confirm the robustness of the microarray data. The PCR primers were designed based on the consensus nucleotide sequences used in the Affymetrix GeneChip® *Plasmodium*/*Anopheles* Genome array design and in the AnoXcel database (http://www.anobase.org/) [63] using Primer 3.0 software (http://www.broad.mit.edu/cgi-bin/primer/primer3_www.cgi). A cohort of female mosquitoes that was independent of those used in the microarray analysis was used for qRT-PCR analysis. Each biological sample consisted of total RNA from three female mosquitoes, and was isolated as described above. Approximately 500 ng of total RNA was used as template for cDNA synthesis using the cDNA Reverse Transcription Kit (Qiagen, Valencia, CA). The qRT-PCR was performed using SYBR Green Master Mix (Fermentas Inc., Glen Burnie, MD) on the MJ Research DNA Engine Opticon RT-PCR System (Bio-RAD). Thermocycler conditions were 94°C for 2 min, followed by 35 cycles of 94°C for 30 s, 58°C for 30 s, and 72°C for 1 min, with a final elongation at 72°C for 10 min. Each thermo cycling reaction was conducted in triplicate, and the ribosomal protein S7 gene was used as a positive control for normalization. The nucleotide sequences of the S7-F and S7-R primers used to amplify the S7 mRNA were 5′-CACCGCCGTGTACGATGCCA-3′ and 5′-ATGGTGGTCTGCTGGTTCTT-3′. Quantification of transcription in the qRT-PCR used the delta-delta Ct method [64]. The average value of the triplicates was used extraneous cDNA production was evaluated by visualization of 5 µL of qRT-PCR product on a 2% agarose gel using ethidium bromide staining.

## Supporting Information

Table S1 **A list of 248 desiccation-responsive transcripts, including transcript identity, start and end points, expression intensity, regulation under desiccation, statistical significance, and gene ontology molecular functions.**
(DOC)

Table S2 **List of** ***Anopheles gambiae*** **desiccation-responsive genes located in the 2La and 2Rs chromosomal inversions.** The order or the gene list in this table is based on the start position on the chromosome.
(DOC)
