# Supplementary material for: Genome-Wide Transcriptional Analysis of Genes Associated with Acute Desiccation Stress in Anopheles gambiae
Source: PLoS One. 2011 Oct 4;6(10):e26011. doi: 10.1371/journal.pone.0026011 (PMC3186805; doi:10.1371/journal.pone.0026011)
Supplement: Table S2 — List of Anopheles gambiae desiccation-responsive genes located in the 2La and 2Rs chromosomal inversions. The order or the gene list in this table is based on the start position on the chromosome. (DOC) [file pone.0026011.s002.doc]

**Table S2** List of *Anopheles gambiae* desiccation-responsive genes located in the 2La and 2Rs chromosomal inversions. The order or the gene list in this table is based on the start position on the chromosome.

| **Affymatrix**  **Probe Set ID** | **Ensembl ID** | **Position** | **Regulation under desiccation**** | **Gene Ontology Molecular Function** |
| --- | --- | --- | --- | --- |
| Ag.2L.2621.0_CDS_at | AGAP006581 | 2La | **↓** | trypsin Inhibitor like cysteine rich domain |
| Ag.2L.1085.0_CDS_at | AGAP005887 | 2La | **↓** | specific transcriptional repressor activity |
| Ag.2L.700.0_CDS_a_at | AGAP005890 | 2La | **↓** | transcription factor |
| Ag.2L.2508.0_CDS_a_at | AGAP005926* | 2La | **↑** | ATP-dependent RNA helicase activity/ Forkhead associated domain |
| Ag.2L.1996.2_at | AGAP005952* | 2La | **↓** | eye morphogenesis |
| Ag.2L.1996.0_CDS_at | AGAP005952 | 2La | **↓** | binding |
| Ag.2L.651.0_CDS_at | AGAP005987 | 2La | **↑** | negative regulation of neuroblast proliferation |
| Ag.2L.619.0_CDS_at | AGAP005998(CPR23) | 2La | **↓** | structural molecule activity |
| Ag.2L.1645.0_CDS_at | AGAP005999(CPR24) | 2La | **↓** | structural molecule activity |
| Ag.2L.1655.0_CDS_at | AGAP006052 | 2La | **↓** | protein phosphatase type 1 regulator activity |
| Ag.2L.990.0_CDS_s_at | AGAP006178 | 2La | **↓** | calcium ion binding |
| Ag.2L.1221.0_CDS_at | AGAP006145(CPLC2) | 2La | **↓** | unknown |
| Ag.2L.246.0_CDS_at | AGAP006149(CPLC17) | 2La | **↓** | structural molecule activity |
| Ag.2L.2030.0_CDS_a_at | AGAP006151 | 2La | **↓** | unknown |
| Ag.2L.2030.1_at | AGAP006151 | 2La | **↓** | unknown |
| Ag.2L.288.0_CDS_a_at | AGAP006181 | 2La | **↓** | calcium ion binding |
| Ag.2L.2571.0_CDS_at | AGAP006257 | 2La | **↓** | unknown |
| Ag.2L.39.0_CDS_at | AGAP006430(CTLGA2) | 2La | **↓** | binding |
| Ag.2L.2854.2_s_at | AGAP006433 | 2La | **↓** | chitin binding |
| Ag.2L.24.0_CDS_s_at | AGAP006434 | 2La | **↓** | chitin binding |
| Ag.2L.2845.0_at | AGAP006434 | 2La | **↓** | chitin binding |
| Ag.2L.658.0_CDS_a_at | AGAP006467 | 2La | **↓** | unknown |
| Ag.2L.2603.1_at | AGAP006480 | 2La | **↓** | unknown |
| Ag.2L.3961.0_at | AGAP006586 | 2La | **↓** | trypsin Inhibitor like cysteine rich domain |
| Ag.2L.918.0_UTR_a_at | AGAP006829(CPR59) | 2La | **↓** | structural molecule activity |
| Ag.2L.918.0_CDS_a_at | AGAP006829 | 2La | **↓** | structural molecule activity |
| Ag.2L.3526.0_at | --- | 2La | **↓** | unknown |
| Ag.2L.1716.0_CDS_at | AGAP006898* | 2La | **↓** | chitinase activity |
| Ag.2L.1266.0_CDS_at | AGAP006964* | 2La | **↓** | pyroglutamyl-peptidase I activity (Enzymes responsible for cleaving pyroglutamate (pGlu) from the N-terminal end of specialized proteins) |
| Ag.2L.167.3_s_at | AGAP007050* | 2La | **↑** | GTP binding |
| Ag.2R.300.31_UTR_a_at | AGAP002350* | 2Rb | **↓** | troponin T type 3,adult muscle formation |
| Ag.2R.300.0_CDS_a_at | AGAP002350 | 2Rb | **↓** | binding |
| Ag.2R.662.0_UTR_at | AGAP002352 | 2Rb | **↑** | transcription factor activity |
| Ag.2R.662.0_CDS_at | AGAP002352 | 2Rb | **↑** | transcription factor activity |
| Ag.2R.1390.0_UTR_at | AGAP002386* | 2Rb | **↑** | heat shock protein binding |
| Ag.2R.2584.0_CDS_at | AGAP002404* | 2Rb | **↑** | ubiquitin protein ligase binding |
| Ag.2R.1393.0_CDS_at | AGAP002456* | 2Rb | **↓** | calcium ion binding |
| Ag.2R.3248.0_CDS_at | AGAP002479 | 2Rb | **↑** | unknown |
| Ag.2R.335.0_CDS_at | AGAP002499* | 2Rb | **↑** | valine, leucine and isoleucine degradation |
| Ag.2R.378.11_at | AGAP002737* | 2Rb | **↓** | Ribosomal protein S8e |
| Ag.2R.365.0_CDS_a_at | AGAP002830 | 2Rc | **↑** | catalytic activity |
| Ag.2R.1108.0_CDS_at | AGAP002848 | 2Rc | **↓** | binding |
| Ag.2R.769.0_CDS_at | AGAP002929 | 2Rc | **↑** | chromatin remodeling |
| Ag.2R.473.0_CDS_at | AGAP002936 | 2Rc | **↓** | unknown |
| Ag.2R.351.0_CDS_at | AGAP002994 | 2Rc | **↓** | structural constituent of cuticle |
| Ag.2R.591.0_CDS_a_at | AGAP003027 | 2Rc | **↓** | unknown |
| Ag.2R.1411.0_CDS_a_at | AGAP003030* | 2Rc | **↓** | catalytic activity |
| Ag.2R.1718.0_CDS_a_at | AGAP003037* | 2Rc | **↓** | structural constituent of cuticle |
| Ag.2R.442.0_UTR_at | AGAP003308* | 2Rd | **↓** | chitin binding |
| Ag.2R.441.1_UTR_s_at | AGAP003308* | 2Rd | **↓** | chitin binding |
| Ag.2R.442.1_CDS_s_at | AGAP003308* | 2Rd | **↓** | chitin binding |
| Ag.2R.1304.0_CDS_at | AGAP003397* | 2Rd | **↑** | helicase activity |
| Ag.2R.304.11_at | AGAP003439* | 2Rd | **↑** | protein binding |
| Ag.2R.5132.0_at | --- | 2Rd | **↑** | unknown |
| Ag.2R.1307.1_at | AGAP003486 | 2Rd | **↑** | unknown |
| Ag.2R.1309.1_CDS_s_at | AGAP003525* | 2Rd | **↑** | RNA degradation |
| Ag.2R.899.0_CDS_a_at | AGAP003652* | 2Rd | **↑** | oxidoreductase activity |
| Ag.2R.2003.0_CDS_at | AGAP003714 | 2Rd | **↓** | Animal haem peroxidase |
| Ag.2R.2004.0_CDS_at | --- | 2Rd | **↓** | catalytic activity |
| Ag.2R.1128.0_CDS_a_at | AGAP003734 | 2Rd | **↓** | Sec14p-like lipid-binding domain |
| Ag.2R.2009.0_CDS_at | AGAP003746* | 2Rd | **↓** | cell surface flocculin with structure similar to serine |
| Ag.2R.2291.0_CDS_at | AGAP003760 | 2Rd | **↓** | Protein Kinases, catalytic activity |
| Ag.2R.353.0_CDS_at | AGAP003769* | 2Rd | **↑** | Armadillo/beta-catenin-like repeats,segment polarity gene armadillo |
| Ag.2R.3684.0_at | --- | 2Rd/2Ru | **↑** | unknown |
| Ag.2R.847.0_CDS_at | AGAP003184 | 2Rd/2Ru | **↓** | catalytic activity |
| Ag.2R.1575.1_CDS_a_at | AGAP003261 | 2Rd/2Ru | **↓** | structural molecule activity |
| Ag.2R.2762.0_CDS_s_at | AGAP003289* | 2Rd/2Ru | **↓** | catalyze the reversible hydration of carbon dioxide |

* The functions of the genes are conserved in other organisms.

****** “↑’’ increased expression in response to desiccation, and “↓’’ decreased expression in response to desiccation.
