# Supplementary material for: Genome-Wide Transcriptional Analysis of Genes Associated with Acute Desiccation Stress in Anopheles gambiae
Source: PLoS One. 2011 Oct 4;6(10):e26011. doi: 10.1371/journal.pone.0026011 (PMC3186805; doi:10.1371/journal.pone.0026011)
Supplement: Supplementary file 2 [file pone.0026011.s001.doc]

## Sheet: S1

Probe Set ID	Gene_Start	Gene_End	Alignments	Regulation under desiccation	Ensembl	Chromosome 	Seq name	Mean Treatment 0hr	Mean Treatment 18hr30%	Mean Treatment 18hr70%	Mean Treatment 36hr30%	Mean Treatment 36hr70%	RSquare	Log of (30%-70%)@18hr	Log of (30%-70%)@36hr	Function assignment	Gene Ontology Biological Process	Gene Ontology Cellular Component	Gene Ontology Molecular Function	Std Mean Treatment 0hr	Std Mean Treatment 18hr30%	Std Mean Treatment 18hr70%	Std Mean Treatment 36hr30%	Std Mean Treatment 36hr70%	Diff of Treatment = (18hr30%) - (0hr)	Diff of Treatment = (18hr70%) - (0hr)	Diff of Treatment = (36hr30%) - (0hr)	Diff of Treatment = (36hr70%) - (0hr)	t Value for Diff of Treatment = (18hr30%) - (0hr)	t Value for Diff of Treatment = (18hr70%) - (0hr)	t Value for Diff of Treatment = (36hr30%) - (0hr)	t Value for Diff of Treatment = (36hr70%) - (0hr)	Prob>|t| for Diff of Treatment = (18hr30%) - (0hr)	Prob>|t| for Diff of Treatment = (18hr70%) - (0hr)	Prob>|t| for Diff of Treatment = (36hr30%) - (0hr)	Prob>|t| for Diff of Treatment = (36hr70%) - (0hr)	-log10(p-value) for Diff of Treatment = (18hr30%) - (0hr)	-log10(p-value) for Diff of Treatment = (18hr70%) - (0hr)	-log10(p-value) for Diff of Treatment = (36hr30%) - (0hr)	-log10(p-value) for Diff of Treatment = (36hr70%) - (0hr)
Ag.2L.1533.0_CDS_at	3748880	3749485	2L:3748878-3749567 (-) // 100.0 //	Up	AGAP004803	2L	ENSANGP00000011431	6.752178	7.459504	7.837221	7.66488	7.687202	0.897966	-0.3777170000000005	-0.022321999999999953	binding	---	---	---	-1.69851	-0.04828	0.832959	0.430876	0.482956	0.707326	1.085043	0.912702	0.935024	6.279681	9.633076	7.501937	8.301197	0.00001484	0.00000008244	0.000001898	0.0000005496	4.828462	7.08387	5.721765	6.259962
Ag.2L.947.0_CDS_at	4243691	4249872	2L:4243333-4252301 (-) // 100.0 //	Down	AGAP004834	2L	ENSANGP00000013108	8.535045	7.172813	7.638492	7.212489	7.435351	0.848439	-0.4656790000000006	-0.22286200000000012	transporter activity	---	0016020 // membrane // inferred from electronic annotation	---	1.684505	-0.76654	0.071349	-0.69515	-0.29416	-1.36223	-0.89655	-1.32256	-1.09969	-7.83817	-5.15869	-7.04538	-6.32755	0.000001116	0.000117	0.000004	0.00001364	5.952423	3.931459	5.398316	4.865108
Ag.2L.1544.0_CDS_at	4497020	4497507	2L:4496967-4497555 (-) // 100.0 //	Up	AGAP004857	2L	ENSANGP00000025807	6.800491	7.7567	7.598843	8.120111	8.093584	0.815858	0.1578570000000008	0.026526999999999745	catalytic activity	0006508 // proteolysis // inferred from electronic annotation	---	0003824 // catalytic activity // inferred from electronic annotation /// 0004252 // serine-type endopeptidase activity // inferred from electronic annotation	-1.6286	0.154299	-0.14003	0.831896	0.782434	0.956209	0.798353	1.319621	1.293093	5.128312	4.281699	6.552351	6.935077	0.000124	0.000657	0.000009218	0.000004804	3.906149	3.182253	5.035387	5.318366
Ag.2L.544.0_CDS_at	4502064	4503060	2L:4501582-4503073 (-) // 100.0 //	Up	AGAP004859	2L	ENSANGP00000013154	9.485095	10.69439	10.51436	11.21532	10.94927	0.909353	0.18003000000000036	0.2660499999999999	catalytic activity	0006508 // proteolysis // inferred from electronic annotation	---	0003824 // catalytic activity // inferred from electronic annotation /// 0004252 // serine-type endopeptidase activity // inferred from electronic annotation	-1.64003	0.185203	-0.08652	0.971456	0.569896	1.209299	1.029269	1.730227	1.464175	7.873253	6.701147	10.42918	9.532645	0.000001057	0.000007138	0.00000002902	0.00000009447	5.97613	5.146435	7.5373	7.024693
Ag.2L.188.0_CDS_a_at	4852047	4865278	2L:4851782-4866265 (+) // 100.0 //	Down	AGAP004877	2L	ENSANGP00000013163	11.72163	10.84471	10.63993	10.8421	10.77627	0.882047	0.20477999999999952	0.06583000000000006	motor activity	---	0016459 // myosin complex // inferred from electronic annotation	0003774 // motor activity // inferred from electronic annotation	1.755324	-0.27887	-0.7539	-0.28492	-0.43764	-0.87692	-1.0817	-0.87953	-0.94536	-7.18987	-8.86887	-6.67631	-7.75104	0.000003148	0.0000002389	0.000007447	0.000001279	5.50198	6.621738	5.127993	5.893257
Ag.2L.211.0_CDS_at	4939314	4940319	UNKN:22707085-22707704 (+) // 66.09 // /// 2L:4939314-4940310 (+) // 99.03 // /// 2L:4939314-4940319 (+) // 100.0 //	Down	AGAP004883	2L	ENSANGT00000013165	12.439	11.41746	11.67465	11.06021	11.76736	0.843036	-0.2571899999999996	-0.7071500000000004	unknown	---	---	---	1.50729	-0.49951	0.005726	-1.20135	0.187848	-1.02153	-0.76435	-1.37879	-0.67164	-6.46171	-4.83488	-8.07458	-4.24847	0.00001079	0.000219	0.0000007747	0.000703	4.967091	3.65915	6.110882	3.153201
Ag.2L.681.0_CDS_at	5769012	5769658	2L:5768979-5769889 (-) // 100.0 //	Up	AGAP004903	2L	ENSANGP00000021352	10.66776	11.06691	11.16484	11.0747	11.26199	0.816042	-0.09792999999999985	-0.18729000000000084	unknown	---	---	---	-1.67588	0.08685	0.51935	0.121279	0.948398	0.399142	0.497075	0.406938	0.594226	4.507302	5.613207	4.254455	6.710285	0.000418	0.00004966	0.000694	0.000007027	3.378361	4.303999	3.158436	5.153211
Ag.2L.733.0_CDS_a_at	8693251	8695812	2L:8689348-8695897 (+) // 100.0 //	Down	AGAP005038	2L	ENSANGP00000029450	7.584484	6.888341	6.375098	6.389039	6.375185	0.851503	0.5132429999999992	0.013854000000000255	signal transducer activity	---	---	---	1.62693	0.313119	-0.65551	-0.6292	-0.65534	-0.69614	-1.20939	-1.19545	-1.2093	-4.22557	-7.34093	-6.71804	-7.3404	0.000736	0.00000246	0.000006935	0.000002462	3.133154	5.609066	5.158957	5.608692
Ag.2L.287.0_CDS_a_at	9725791	9746130	2L:9725791-9746130 (+) // 100.0 //	Down	AGAP005079	2L	ENSANGT00000025205	9.97768	8.759754	8.843405	8.902978	9.077836	0.761379	-0.08365100000000147	-0.1748580000000004	binding	0006471 // protein amino acid ADP-ribosylation // inferred from electronic annotation /// 0007165 // signal transduction // inferred from electronic annotation /// 0007186 // G-protein coupled receptor protein signaling pathway // inferred from electronic	---	0000166 // nucleotide binding // inferred from electronic annotation /// 0004871 // signal transducer activity // inferred from electronic annotation /// 0005525 // GTP binding // inferred from electronic annotation /// 0019001 // guanyl nucleotide bindin	1.738997	-0.70853	-0.54043	-0.42071	-0.06932	-1.21793	-1.13428	-1.0747	-0.89984	-5.89843	-5.49331	-4.8187	-4.35795	0.0000294	0.00006211	0.000226	0.000564	4.531677	4.206866	3.645398	3.248765
Ag.2L.1571.0_CDS_at	10566416	10569237	2L:10566414-10569430 (-) // 100.0 //	Up	AGAP005147	2L	ENSANGP00000027880	6.523654	7.340373	7.540881	7.403179	7.398969	0.698984	-0.20050800000000013	0.004209999999999603	binding	---	---	---	-1.75949	0.242593	0.734111	0.396552	0.386233	0.81672	1.017227	0.879525	0.875316	4.106795	5.115028	4.094537	4.401439	0.000936	0.000127	0.000959	0.000517	3.028906	3.895065	3.018122	3.286593
Ag.2L.3267.0_at	10590619	10590965	2L:10590619-10590965 (+) // 100.0 //	Up	---	2L		7.357924	8.181337	8.042396	8.301178	7.900777	0.828631	0.1389409999999991	0.40040100000000045	unknown	---	---	---	-1.63255	0.612385	0.233578	0.939116	-0.15253	0.823413	0.684472	0.943254	0.542853	6.577602	5.467708	6.975977	4.336431	0.000008824	0.00006516	0.000004486	0.000589	5.054325	4.186022	5.348096	3.230014
Ag.2L.53.1_CDS_at	14202579	14217941	2L:14202579-14217941 (+) // 100.0 //	Down	AGAP005333	2L		5.807993	4.161022	3.921915	3.709072	4.153138	0.800322	0.23910700000000018	-0.4440660000000003	unknown	---	---	---	1.743674	-0.22686	-0.51294	-0.76759	-0.23629	-1.64697	-1.88608	-2.09892	-1.65485	-5.45545	-6.24747	-6.43676	-5.48156	0.00006668	0.00001571	0.00001127	0.00006349	4.176027	4.803723	4.948208	4.197309
Ag.2L.53.0_CDS_at	14230351	14230962	2L:14222516-14231188 (+) // 100.0 //	Down	AGAP005332	2L	ENSANGP00000020938	5.712701	4.579867	4.031112	4.06255	4.389421	0.87636	0.5487549999999999	-0.3268709999999997	binding	---	---	0005488 // binding // inferred from electronic annotation /// 0005529 // sugar binding // inferred from electronic annotation	1.686128	0.036032	-0.76329	-0.7175	-0.24137	-1.13283	-1.68159	-1.65015	-1.32328	-5.97203	-8.86493	-8.05389	-6.97602	0.00002572	0.0000002403	0.0000007996	0.000004486	4.589649	6.619286	6.097137	5.348124
Ag.2L.444.1_a_at	15758482	15760042	2L:15758482-15760042 (+) // 99.45 //	Down	AGAP005456	2L		11.87992	10.10371	10.67431	9.052765	10.38743	0.910323	-0.5706000000000007	-1.3346649999999993	structural molecule activity	---	---	0042302 // structural constituent of cuticle // inferred from electronic annotation	1.430824	-0.30954	0.24954	-1.33928	-0.03155	-1.77621	-1.20562	-2.82716	-1.49249	-7.94308	-5.39142	-11.705	-6.67432	0.0000009482	0.00007522	0.000000006155	0.000007473	6.023117	4.123681	8.210748	5.126511
Ag.2L.444.0_CDS_a_at	15797749	15801337	2L:15758482-15759877 (+) // 100.0 //	Down	AGAP005456	2L	ENSANGP00000014280	11.57606	9.442131	9.980022	8.229869	9.841476	0.933286	-0.5378910000000001	-1.6116069999999993	structural molecule activity	---	---	0042302 // structural constituent of cuticle // inferred from electronic annotation	1.465796	-0.30926	0.138175	-1.31764	0.022929	-2.13393	-1.59604	-3.34619	-1.73458	-9.49189	-7.0993	-13.78	-7.71557	0.00000009987	0.000003655	0.00000000065	0.000001352	7.000547	5.437145	9.18732	5.869043
Ag.2L.872.0_UTR_a_at	15800917	15801951	2L:15800917-15801951 (+) // 100.0 //	Down	AGAP005459	2L	ENSANGT00000014280	12.04389	10.66203	10.94383	10.1268	10.63804	0.949585	-0.2818000000000005	-0.5112400000000008	structural molecule activity	---	---	0042302 // structural constituent of cuticle // inferred from electronic annotation	1.628868	-0.30991	0.085459	-1.06085	-0.34357	-1.38186	-1.10006	-1.91709	-1.40585	-11.2782	-8.97831	-14.4859	-11.474	0.00000001018	0.0000002043	0.000000000323	0.000000008066	7.992295	6.689626	9.490989	8.093345
Ag.2L.203.0_CDS_at	17369577	17371537	2L:17369399-17371537 (+) // 100.0 //	Up	AGAP005581	2L	ENSANGP00000017720	9.752053	10.59237	10.59935	10.68852	10.56113	0.822571	-0.006979999999998654	0.12739000000000011	catalytic activity	0006098 // pentose-phosphate shunt // inferred from electronic annotation /// 0006573 // valine metabolic process // inferred from electronic annotation /// 0008152 // metabolic process // inferred from electronic annotation	---	0003824 // catalytic activity // inferred from electronic annotation /// 0004616 // phosphogluconate dehydrogenase (decarboxylating) activity // inferred from electronic annotation /// 0005488 // binding // inferred from electronic annotation /// 0008442 	-1.77538	0.397369	0.415426	0.64599	0.316599	0.840314	0.847298	0.936469	0.809076	6.189531	6.24097	6.386102	5.959441	0.00001742	0.0000159	0.00001231	0.00002632	4.75907	4.798724	4.909752	4.579756
Ag.2L.156.0_CDS_a_at	17980918	18000155	2L:17980918-18000080 (+) // 96.47 // /// 2L:17980918-18000155 (+) // 100.0 //	Down	AGAP005627	2L	ENSANGT00000024748	11.49066	10.72712	10.84771	10.56447	10.89229	0.886066	-0.12058999999999997	-0.3278199999999991	catalytic activity	---	---	0003824 // catalytic activity // inferred from electronic annotation /// 0016301 // kinase activity // inferred from electronic annotation /// 0016740 // transferase activity // inferred from electronic annotation /// 0016772 // transferase activity, tran	1.668021	-0.50458	-0.16146	-0.96739	-0.03459	-0.76355	-0.64296	-0.9262	-0.59837	-7.77896	-6.5504	-8.73605	-6.09618	0.000001224	0.000009249	0.0000002894	0.00002057	5.912259	5.033925	6.538552	4.686697
Ag.2L.156.1_CDS_s_at	17989785	18000155	2L:17989785-18000080 (+) // 96.49 // /// 2L:17989785-18000155 (+) // 100.0 //	Down	AGAP005627 /// AGAP012924	2L	ENSANGP00000022538	9.553733	8.616233	8.674916	8.297196	8.714086	0.881364	-0.05868300000000026	-0.4168900000000004	catalytic activity	---	---	0003824 // catalytic activity // inferred from electronic annotation /// 0016301 // kinase activity // inferred from electronic annotation /// 0016740 // transferase activity // inferred from electronic annotation /// 0016772 // transferase activity, tran	1.67441	-0.33167	-0.2061	-1.01435	-0.12228	-0.9375	-0.87882	-1.25654	-0.83965	-7.3405	-6.88102	-9.1087	-6.57433	0.000002462	0.000005261	0.0000001699	0.000008874	5.608761	5.278924	6.769752	5.051871
Ag.2L.2621.0_CDS_at	21515255	21515776	2L:34267245-34277766 (-) // 100.0 //	Down	AGAP006581	2La	ENSANGP00000029754	11.17685	9.261774	9.257488	7.621956	9.479731	0.894102	0.004286000000000456	-1.8577749999999993	binding	---	---	---	1.441633	-0.07757	-0.08097	-1.37842	0.09533	-1.91508	-1.91937	-3.5549	-1.69712	-6.39719	-6.41151	-10.994	-5.66912	0.000012	0.00001177	0.00000001435	0.00004477	4.918183	4.92906	7.843096	4.34901
Ag.2L.1085.0_CDS_at	23173292	23175777	2L:23173124-23175778 (+) // 100.0 //	Down	AGAP005887	2La	ENSANGP00000014168	7.469832	6.785436	6.579993	6.520557	6.810759	0.887423	0.20544299999999982	-0.29020199999999985	transcription regulator activity	---	---	---	1.686256	-0.12684	-0.6711	-0.82856	-0.05976	-0.6844	-0.88984	-0.94928	-0.65907	-6.55767	-8.52615	-8.42094	-6.31503	0.000009133	0.0000003933	0.0000004596	0.000014	5.039378	6.405291	6.337647	4.855541
Ag.2L.700.0_CDS_a_at	23185086	23185598	2L:23184932-23185695 (-) // 100.0 //	Down	AGAP005890	2La	ENSANGP00000013599	11.3252	10.20662	10.47611	9.597905	10.08607	0.851513	-0.2694900000000011	-0.48816499999999863	transcription regulator activity	---	---	---	1.54963	-0.20691	0.216277	-1.16279	-0.3962	-1.11858	-0.84909	-1.72729	-1.23913	-5.88896	-4.47019	-8.41906	-6.52358	0.00003	0.000451	0.0000004609	0.000009688	4.524197	3.346239	6.336434	5.013765
Ag.2L.2508.0_CDS_a_at	23798973	23799393	2L:23798845-23804811 (-) // 100.0 //	Up	AGAP005926	2La	ENSANGP00000013515	5.508872	6.182145	6.613896	6.500303	6.235493	0.888868	-0.4317510000000002	0.26480999999999977	catalytic activity	---	---	---	-1.62533	-0.06042	0.943106	0.679078	0.063574	0.673274	1.105024	0.991431	0.726622	5.760791	9.455019	7.853797	6.217258	0.00003781	0.000000105	0.000001089	0.00001658	4.422407	6.978638	5.962992	4.780464
Ag.2L.1996.2_at	23918188	23926525	2L:24033822-24034701 (+) // 100.0 //	Down	AGAP005952	2La	ENSANGP00000031641	10.17586	8.853931	9.322499	8.048257	9.032158	0.939284	-0.4685680000000012	-0.9839010000000012	binding	---	---	---	1.412978	-0.30172	0.306064	-1.34678	-0.07054	-1.32193	-0.85337	-2.12761	-1.14371	-9.40798	-6.07326	-14.0186	-8.13956	0.000000112	0.00002144	0.000000000511	0.0000007016	6.950594	4.668849	9.29143	6.153909
Ag.2L.1996.0_CDS_at	24030658	24034725	2L:24030658-24034725 (+) // 100.0 //	Down	AGAP005952	2La	ENSANGP00000031890	9.112235	7.934899	8.285023	7.269963	8.04397	0.952612	-0.350124000000001	-0.7740070000000001	binding	---	---	---	1.476118	-0.29179	0.233959	-1.29027	-0.12801	-1.17734	-0.82721	-1.84227	-1.06826	-10.6738	-7.49959	-15.4632	-9.68499	0.00000002131	0.000001905	0.000000000129	0.00000007686	7.671315	5.720133	9.890672	7.114283
Ag.2L.651.0_CDS_at	24547015	24555776	2L:24546616-24555968 (+) // 99.9 // /// 2L:24546616-24555969 (+) // 99.95 //	Up	AGAP005987	2La	ENSANGP00000010885	7.241222	8.825477	8.863738	8.987084	8.815374	0.864417	-0.03826100000000032	0.17170999999999914	signal transducer activity	---	---	---	-1.78106	0.380535	0.43274	0.601036	0.366751	1.584255	1.622516	1.745862	1.574152	7.419103	7.598282	7.569425	7.371792	0.000002168	0.000001627	0.000001704	0.00000234	5.663968	5.788503	5.768568	5.630781
Ag.2L.619.0_CDS_at	24621452	24621891	2L:24621219-24621964 (-) // 100.0 // /// 2L:24621229-24621964 (-) // 98.39 //	Down	AGAP005998	2La	ENSANGP00000023428	8.766198	6.498116	6.799121	5.697012	6.661323	0.982957	-0.30100500000000086	-0.9643110000000004	structural molecule activity	---	---	0042302 // structural constituent of cuticle // inferred from electronic annotation	1.657304	-0.34015	-0.07506	-1.04567	-0.19642	-2.26808	-1.96708	-3.06919	-2.10488	-20.0828	-17.4176	-25.1603	-18.6377	0.00000000000304	0.0000000000236	0.000000000000114	0.00000000000894	11.51696	10.6265	12.94341	11.04871
Ag.2L.1645.0_CDS_at	24624266	24624595	2L:24624132-24624667 (-) // 100.0 //	Down	AGAP005999	2La	ENSANGP00000010846	8.104715	6.275194	6.324524	5.773939	5.770049	0.855616	-0.04933000000000032	0.003890000000000171	structural molecule activity	---	---	0042302 // structural constituent of cuticle // inferred from electronic annotation	1.719942	-0.18133	-0.13007	-0.70225	-0.70629	-1.82952	-1.78019	-2.33078	-2.33467	-6.37312	-6.20128	-7.51695	-8.13279	0.00001259	0.000017	0.000001853	0.0000007089	4.899871	4.768138	5.732199	6.149433
Ag.2L.1655.0_CDS_at	25646726	25650336	2L:25646467-25648619 (-) // 100.0 //	Down	AGAP006052	2La	ENSANGP00000012516	7.539401	6.770052	6.586428	6.674068	6.539944	0.788926	0.183624	0.1341239999999999	enzyme regulator activity	---	---	---	1.747348	-0.12647	-0.5737	-0.36025	-0.68692	-0.76935	-0.95297	-0.86533	-0.99946	-4.8127	-5.96137	-5.01159	-6.25216	0.000229	0.00002623	0.000155	0.00001558	3.640299	4.581274	3.80844	4.807327
Ag.2L.990.0_CDS_s_at	26162257	26162935	2L:27787695-27792177 (-) // 100.0 //	Down	AGAP006178	2La	ENSANGP00000031431	9.877802	8.726603	8.862055	8.133599	8.833794	0.923028	-0.13545199999999902	-0.700194999999999	binding	0001539 // ciliary or flagellar motility // inferred from electronic annotation	0009288 // flagellin-based flagellum // inferred from electronic annotation	0005509 // calcium ion binding // inferred from electronic annotation	1.577603	-0.25497	-0.03934	-1.19896	-0.08433	-1.1512	-1.01575	-1.7442	-1.04401	-8.68075	-7.65936	-12.1767	-7.87247	0.0000003136	0.000001477	0.000000003591	0.000001058	6.503658	5.83054	8.444779	5.9756
Ag.2L.1221.0_CDS_at	27150725	27151406	2L:27150725-27151406 (-) // 100.0 //	Down	AGAP006145	2La	ENSANGP00000031226	6.689534	5.100078	4.843878	4.073989	4.474892	0.837188	0.25619999999999976	-0.40090299999999957	unknown	---	---	---	1.649867	0.063481	-0.19222	-0.96063	-0.5605	-1.58946	-1.84566	-2.61554	-2.21464	-5.03639	-5.84819	-7.67289	-7.01736	0.000148	0.00003222	0.000001446	0.000004187	3.829255	4.491921	5.839829	5.378078
Ag.2L.246.0_CDS_at	27173461	27174125	2L:27173055-27174213 (-) // 100.0 // /// 2L:27173061-27174213 (-) // 99.34 //	Down	AGAP006149	2La	ENSANGP00000017752	8.169304	5.748482	5.941735	5.200474	5.687831	0.892003	-0.19325300000000034	-0.48735700000000026	structural molecule activity	---	---	---	1.738736	-0.34528	-0.17892	-0.81705	-0.39749	-2.42082	-2.22757	-2.96883	-2.48147	-8.29924	-7.63671	-9.42296	-8.50717	0.0000005512	0.000001531	0.0000001098	0.0000004045	6.258685	5.814979	6.959537	6.393124
Ag.2L.2030.0_CDS_a_at	27207515	27214386	2L:27207515-27214386 (+) // 100.0 //	Down	AGAP006151	2La	ENSANGT00000017812	8.406217	5.142234	5.096107	4.598327	5.484994	0.937079	0.04612700000000025	-0.8866670000000001	unknown	---	---	---	1.749729	-0.39678	-0.42711	-0.75447	-0.17137	-3.26398	-3.31011	-3.80789	-2.92122	-11.4347	-11.5963	-12.3506	-10.2339	0.00000000845	0.000000006987	0.000000002957	0.00000003727	8.073151	8.155725	8.529154	7.428585
Ag.2L.2030.1_at	27207752	27210858	2L:27207515-27211138 (+) // 100.0 //	Down	AGAP006151	2La	ENSANGP00000017812	8.780046	5.80605	5.902259	4.423021	6.161534	0.930826	-0.09620899999999999	-1.7385129999999993	unknown	---	---	---	1.617812	-0.25763	-0.19695	-1.12978	-0.03345	-2.974	-2.87779	-4.35703	-2.61851	-9.74746	-9.43213	-13.2211	-8.58234	0.00000007068	0.0000001084	0.000000001156	0.0000003621	7.150714	6.965004	8.937202	6.44118
Ag.2L.288.0_CDS_a_at	27817332	27818435	2L:27817332-27818435 (+) // 100.0 // /// 2L:27817332-27818425 (+) // 98.88 //	Down	AGAP006181	2La	ENSANGT00000017152	12.65111	12.09513	12.10169	11.80732	11.88469	0.915486	-0.006560000000000343	-0.07737000000000016	binding	---	---	0005509 // calcium ion binding // inferred from electronic annotation	1.64611	-0.03898	-0.01909	-0.91126	-0.67678	-0.55598	-0.54942	-0.84378	-0.76642	-6.76764	-6.68776	-9.50903	-9.3292	0.000006373	0.000007303	0.00000009756	0.0000001249	5.195636	5.136497	7.010711	6.9034
Ag.2L.2571.0_CDS_at	28684634	28685317	2L:28684633-28685872 (-) // 100.0 //	Down	AGAP006257	2La	ENSANGP00000028165	7.216142	6.367228	6.31428	5.429547	6.326062	0.902306	0.05294799999999977	-0.896515	structural molecule activity	---	---	---	1.401086	0.057874	-0.0259	-1.42579	-0.00726	-0.84891	-0.90186	-1.78659	-0.89008	-5.76532	-6.12491	-11.2334	-6.04489	0.0000375	0.00001954	0.00000001074	0.00002256	4.426017	4.709027	7.968975	4.64672
Ag.2L.39.0_CDS_at	31772242	31780811	2L:31772153-31780978 (+) // 100.0 //	Down	AGAP006430	2La	ENSANGP00000032027	7.66524	6.558284	6.168622	6.152792	6.648471	0.862122	0.3896619999999995	-0.495679	binding	---	---	0005488 // binding // inferred from electronic annotation	1.666709	-0.13053	-0.76318	-0.78889	0.015893	-1.10696	-1.49662	-1.51245	-1.01677	-6.11705	-8.27032	-7.73781	-5.61867	0.00001982	0.0000005757	0.000001305	0.00004916	4.702923	6.239809	5.884236	4.308407
Ag.2L.2854.2_s_at	31791914	31792798	2L:31792055-31793059 (-) // 93.14 // /// 2L:31791748-31793059 (-) // 100.0 //	Down	AGAP006433	2La	ENSANGP00000032025	8.770216	5.178275	6.715269	3.896682	6.945463	0.926289	-1.536994	-3.048781	chitin binding	0006030 // chitin metabolic process // inferred from electronic annotation	0005576 // extracellular region // inferred from electronic annotation	0008061 // chitin binding // inferred from electronic annotation	1.3329	-0.6062	0.223544	-1.29806	0.347813	-3.59194	-2.05495	-4.87353	-1.82475	-9.91191	-5.6706	-12.4508	-5.03538	0.00000005678	0.00004465	0.000000002646	0.000148	7.245803	4.350199	8.577357	3.828414
Ag.2L.24.0_CDS_s_at	31793430	31795063	2L:31788975-31795126 (+) // 95.08 // /// 2L:31788975-31795126 (+) // 100.0 //	Down	AGAP006434	2La	ENSANGP00000021035	9.587527	5.674169	7.037921	4.387615	6.847561	0.903395	-1.3637519999999999	-2.4599459999999995	chitin binding	0006030 // chitin metabolic process // inferred from electronic annotation	0005576 // extracellular region // inferred from electronic annotation	0008061 // chitin binding // inferred from electronic annotation	1.493857	-0.5356	0.171637	-1.20281	0.072916	-3.91336	-2.54961	-5.19991	-2.73997	-8.88954	-5.79165	-10.9358	-6.22407	0.000000232	0.00003573	0.00000001541	0.00001638	6.634603	4.447006	7.812181	4.785717
Ag.2L.2845.0_at	31794053	31795112	2L:31794295-31795112 (-) // 88.93 // /// 2L:31794053-31795112 (-) // 100.0 //	Down	AGAP006434	2La	ENSANGP00000011037	8.723214	4.773285	6.42736	3.11986	5.963993	0.911057	-1.6540750000000006	-2.8441330000000002	binding	---	---	---	1.408835	-0.49583	0.30177	-1.29311	0.078334	-3.94993	-2.29585	-5.60335	-2.75922	-8.80543	-5.11807	-11.5647	-6.15103	0.0000002617	0.000127	0.00000000725	0.00001865	6.582118	3.897601	8.139679	4.729287
Ag.2L.658.0_CDS_a_at	32532216	32533483	2L:32532216-32533483 (+) // 100.0 //	Down	AGAP006467	2La		11.63018	10.66893	10.84453	10.36196	10.98276	0.878807	-0.1756000000000011	-0.6208000000000009	unknown	---	---	---	1.556467	-0.48604	-0.11292	-1.1383	0.180795	-0.96125	-0.78565	-1.26822	-0.64742	-7.46161	-6.09852	-9.11417	-5.02555	0.000002025	0.00002049	0.0000001686	0.000151	5.693675	4.688524	6.773093	3.820158
Ag.2L.2603.1_at	32842108	32843886	2L:32842108-32843886 (-) // 100.0 //	Down	AGAP006480	2La		12.29971	11.02903	11.1838	10.11968	11.11137	0.916271	-0.15476999999999919	-0.9916900000000002	unknown	---	---	---	1.485725	-0.1545	0.045284	-1.3283	-0.04821	-1.27068	-1.11591	-2.18003	-1.18834	-7.65377	-6.72153	-12.157	-7.15781	0.00000149	0.000006894	0.000000003672	0.000003318	5.826705	5.161546	8.435156	5.479086
Ag.2L.3961.0_at	34276177	34276540	2L:34276025-34276587 (-) // 100.0 //	Down	AGAP006586	2La	ENSANGP00000030923	7.772082	5.283272	5.411696	4.174239	5.445174	0.834918	-0.12842399999999987	-1.2709349999999997	binding	---	---	---	1.639561	-0.25415	-0.15644	-1.09801	-0.13096	-2.48881	-2.36039	-3.59784	-2.32691	-6.04366	-5.7318	-8.08867	-5.65051	0.00002261	0.00003988	0.0000007582	0.00004634	4.645757	4.399252	6.12023	4.334047
Ag.2L.918.0_UTR_a_at	39074449	39077011	2L:39074449-39077011 (+) // 100.0 //	Down	AGAP006829	2La	ENSANGT00000013220	11.60438	7.885936	8.338515	6.748872	7.782507	0.980854	-0.4525789999999992	-1.0336349999999994	structural molecule activity	---	---	0042302 // structural constituent of cuticle // inferred from electronic annotation	1.697647	-0.31765	-0.07237	-0.93391	-0.37371	-3.71845	-3.26587	-4.85551	-3.82188	-19.8402	-17.4254	-23.9853	-20.392	0.00000000000363	0.0000000000235	0.000000000000229	0.00000000000244	11.44057	10.62929	12.63934	11.61307
Ag.2L.918.0_CDS_a_at	39075645	39076184	2L:39074449-39077011 (+) // 100.0 //	Down	AGAP006829	2La	ENSANGP00000013220	10.75241	6.76399	6.968154	6.139629	6.662812	0.987356	-0.20416400000000046	-0.5231829999999995	structural molecule activity	---	---	0042302 // structural constituent of cuticle // inferred from electronic annotation	1.764717	-0.37137	-0.26203	-0.70576	-0.42556	-3.98842	-3.78425	-4.61278	-4.0896	-25.3596	-24.0614	-27.1538	-26.0029	0.000000000000101	0.000000000000219	0.0000000000000372	0.0000000000000703	12.99364	12.65948	13.42948	13.15322
Ag.2L.3526.0_at	39275780	39275994	2L:39275780-39275979 (+) // 92.99 // /// 2L:39275780-39275994 (+) // 100.0 //	Down	---	2La		11.27355	8.797815	9.075567	7.263503	8.712253	0.936429	-0.27775199999999955	-1.4487500000000004	unknown	---	---	---	1.56027	-0.15729	0.035401	-1.22173	-0.21665	-2.47574	-2.19799	-4.01005	-2.5613	-9.3842	-8.33139	-14.0724	-9.70852	0.0000001158	0.0000005253	0.000000000485	0.00000007447	6.936378	6.279621	9.314702	7.128024
Ag.2L.1716.0_CDS_at	39574329	39577645	2L:39574334-39577645 (-) // 100.0 //	Down	AGAP006898	2La	ENSANGP00000021620	5.281546	4.246433	4.11987	3.957123	3.857358	0.873293	0.12656299999999998	0.0997650000000001	catalytic activity	0005975 // carbohydrate metabolic process // inferred from electronic annotation /// 0006030 // chitin metabolic process // inferred from electronic annotation /// 0006032 // chitin catabolic process // inferred from electronic annotation /// 0008152 // m	0005576 // extracellular region // inferred from electronic annotation	0003824 // catalytic activity // inferred from electronic annotation /// 0004553 // hydrolase activity, hydrolyzing O-glycosyl compounds // inferred from electronic annotation /// 0004568 // chitinase activity // inferred from electronic annotation /// 00	1.727034	-0.08038	-0.30137	-0.58554	-0.75974	-1.03511	-1.16168	-1.32442	-1.42419	-6.35684	-7.13409	-7.5302	-8.74623	0.000013	0.000003451	0.000001814	0.0000002851	4.887467	5.462106	5.741399	6.54496
Ag.2L.1266.0_CDS_at	40279733	40280548	2L:40279725-40280600 (-) // 100.0 //	Down	AGAP006964	2La	ENSANGP00000021839	10.36864	8.794048	8.921359	8.458187	8.635623	0.941883	-0.12731100000000062	-0.17743600000000015	catalytic activity	0006508 // proteolysis // inferred from electronic annotation	---	---	1.742246	-0.31566	-0.14927	-0.75461	-0.52271	-1.57459	-1.44728	-1.91045	-1.73302	-11.0669	-10.1721	-12.4314	-12.1803	0.00000001313	0.00000004038	0.000000002704	0.000000003576	7.881636	7.39383	8.568027	8.446549
Ag.2L.167.3_s_at	41829053	41831327	2L:41828919-41831427 (-) // 100.0 //	Up	AGAP007050	2La	ENSANGP00000021465	10.42746	10.83506	10.84215	11.09533	10.86235	0.838672	-0.007089999999999819	0.2329800000000013	binding	---	---	0005525 // GTP binding // inferred from electronic annotation	-1.59825	0.093786	0.123219	1.174196	0.207052	0.407605	0.414696	0.667871	0.434891	4.838739	4.92291	7.34027	5.162648	0.000218	0.000185	0.000002463	0.000116	3.662423	3.733717	5.608599	3.934754
Ag.2L.168.0_CDS_at	44639573	44640831	2L:44638664-44642299 (-) // 100.0 //	Down	AGAP007249	2L	ENSANGP00000016640	12.64215	12.10659	12.14621	12.03536	12.08079	0.858215	-0.03961999999999932	-0.04542999999999964	motor activity	---	---	---	1.765347	-0.38375	-0.22474	-0.66959	-0.48727	-0.53556	-0.49593	-0.60679	-0.56136	-6.40442	-5.93056	-6.71796	-6.71292	0.000012	0.00002773	0.000006936	0.000007	4.923675	4.557028	5.158902	5.155166
Ag.2L.719.1_a_at	44653283	44657449	2L:44653283-44657449 (-) // 98.78 // /// 2L:44653283-44657449 (-) // 100.0 //	Down	AGAP007250	2L		7.855944	6.837664	6.714545	6.662505	6.638383	0.708216	0.12311899999999998	0.0241220000000002	unknown	---	---	---	1.768932	-0.20153	-0.43977	-0.54048	-0.58715	-1.01828	-1.1414	-1.19344	-1.21756	-4.18153	-4.68711	-4.53727	-4.99987	0.000804	0.000293	0.000394	0.000159	3.094555	3.53311	3.404252	3.798583
Ag.2L.786.0_CDS_at	46133328	46134066	2L:46133196-46134337 (+) // 100.0 //	Down	AGAP007371	2L	ENSANGP00000028160	7.80695	4.754569	5.317471	4.211554	5.421377	0.940208	-0.5629020000000002	-1.209823	unknown	---	---	---	1.674416	-0.54334	-0.13435	-0.93787	-0.05886	-3.05238	-2.48948	-3.5954	-2.38557	-12.2307	-9.97515	-13.3378	-9.55881	0.00000000338	0.00000005223	0.000000001023	0.00000009117	8.471061	7.282053	8.990168	7.040156
Ag.2L.1756.0_CDS_at	46355966	46357012	2L:46356311-46357433 (-) // 100.0 //	Up	AGAP007413	2L	ENSANGP00000029644	7.726625	8.318733	8.503048	8.373561	8.262891	0.77585	-0.18431499999999978	0.11067000000000071	binding	---	0005622 // intracellular // inferred from electronic annotation	---	-1.70766	0.27358	0.890312	0.457037	0.086729	0.592109	0.776424	0.646936	0.536267	4.792237	6.283994	4.84758	4.340281	0.000238	0.00001473	0.000214	0.000584	3.622884	4.831769	3.669929	3.233369
Ag.2L.459.0_UTR_a_at	47149951	47187968	2L:47149951-47187968 (+) // 100.0 //	Up	AGAP007523	2L	ENSANGT00000009410	6.886773	7.889649	8.257339	7.940686	7.506302	0.882308	-0.36768999999999963	0.43438400000000055	binding	---	0016459 // myosin complex // inferred from electronic annotation	0000166 // nucleotide binding // inferred from electronic annotation /// 0003774 // motor activity // inferred from electronic annotation /// 0003779 // actin binding // inferred from electronic annotation /// 0005524 // ATP binding // inferred from elect	-1.54111	0.368435	1.068542	0.465612	-0.36148	1.002875	1.370566	1.053912	0.619529	6.91341	9.448116	6.726302	4.270778	0.000004982	0.000000106	0.000006838	0.000672	5.302577	6.974529	5.165078	3.172708
Ag.2L.2790.0_CDS_at	47234189	47238682	2L:47234189-47238682 (-) // 100.0 //	Down	AGAP007530	2L	ENSANGT00000019141	7.458665	6.111128	5.953765	5.864892	5.59418	0.942913	0.15736300000000014	0.2707120000000005	catalytic activity	0006508 // proteolysis // inferred from electronic annotation	---	0008270 // zinc ion binding // inferred from electronic annotation	1.728786	-0.11697	-0.33252	-0.45425	-0.82505	-1.34754	-1.5049	-1.59377	-1.86448	-9.86096	-11.0125	-10.7977	-13.6439	0.00000006075	0.00000001403	0.00000001827	0.000000000746	7.216467	7.852896	7.738245	9.1272
Ag.2L.327.0_CDS_at	47533130	47534969	2L:47525154-47535429 (+) // 100.0 //	Down	AGAP007550	2L	ENSANGP00000011013	10.74511	10.308	10.36282	9.933653	10.38595	0.864729	-0.054819999999999425	-0.4522969999999997	catalytic activity	0006730 // one-carbon compound metabolic process // inferred from electronic annotation	---	0004089 // carbonate dehydratase activity // inferred from electronic annotation /// 0008270 // zinc ion binding // inferred from electronic annotation	1.38017	-0.13562	0.054483	-1.43374	0.13471	-0.43711	-0.38229	-0.81146	-0.35916	-4.98689	-4.36146	-8.57094	-4.09751	0.000163	0.00056	0.0000003682	0.000953	3.787662	3.251818	6.433912	3.020742
Ag.2L.1526.0_CDS_at	49317814	49320731	2L:49317814-49320731 (+) // 100.0 //	Up	---	2L	ENSANGT00000021568	9.112598	9.559732	9.566477	10.11879	9.862271	0.922069	-0.006745000000000445	0.25651900000000083	unknown	---	---	---	-1.40942	-0.22344	-0.20555	1.259399	0.579017	0.447135	0.453879	1.006189	0.749674	5.190281	5.268574	10.81331	8.702118	0.00011	0.000095	0.00000001792	0.000000304	3.957728	4.022592	7.746636	6.51716
Ag.2R.553.0_CDS_at	1285922	1291040	2R:1285746-1291280 (+) // 100.0 //	Down	AGAP001200	2R	ENSANGP00000013922	10.39559	9.133336	9.633174	8.592725	9.217532	0.891309	-0.49983800000000045	-0.6248070000000006	catalytic activity	0005975 // carbohydrate metabolic process // inferred from electronic annotation /// 0005978 // glycogen biosynthetic process // inferred from electronic annotation	---	0003824 // catalytic activity // inferred from electronic annotation /// 0004134 // 4-alpha-glucanotransferase activity // inferred from electronic annotation /// 0004135 // amylo-alpha-1,6-glucosidase activity // inferred from electronic annotation /// 0	1.491752	-0.38911	0.355687	-1.19467	-0.26365	-1.26225	-0.76242	-1.80287	-1.17806	-7.50991	-4.53607	-9.93064	-7.00897	0.000001874	0.000395	0.00000005539	0.000004246	5.727304	3.403215	7.256558	5.372006
Ag.2R.2380.0_CDS_at	1402918	1404841	2R:1402917-1404841 (+) // 100.0 //	Down	AGAP001202	2R	ENSANGP00000013892	5.633295	4.142199	4.686022	3.709342	4.416964	0.832583	-0.5438230000000006	-0.7076220000000002	transporter activity	0006810 // transport // inferred from electronic annotation	0016021 // integral to membrane // inferred from electronic annotation	0005215 // transporter activity // inferred from electronic annotation	1.548179	-0.52085	0.233751	-1.12148	-0.13959	-1.4911	-0.94727	-1.92395	-1.21633	-6.48728	-4.12128	-7.74959	-5.29187	0.00001032	0.000909	0.000001282	0.00009082	4.986413	3.041647	5.892263	4.041825
Ag.2R.1640.0_CDS_at	1747788	1749481	2R:1747888-1749532 (-) // 100.0 //	Up	AGAP001233	2R	ENSANGP00000013907	8.798123	9.30311	9.399339	9.433499	9.406861	0.857473	-0.09622899999999923	0.02663800000000016	Golgin subfamily A member 5	---	---	---	-1.75812	0.130621	0.490535	0.6183	0.518668	0.504987	0.601216	0.635376	0.608738	5.695557	6.780884	6.634579	6.86572	0.00004264	0.000006232	0.000008	0.000005398	4.370227	5.205402	5.096917	5.267727
Ag.2R.3650.0_at	4739064	4740058	2R:4739064-4740058 (+) // 100.0 //	Down	---	2R		11.73908	11.34414	11.27783	11.16436	11.14366	0.891242	0.06630999999999965	0.02069999999999972	unknown	---	---	---	1.681491	0.042846	-0.23229	-0.70306	-0.78898	-0.39494	-0.46125	-0.57472	-0.59543	-5.40562	-6.31325	-7.28274	-8.14969	0.00007323	0.000014	0.000002704	0.0000006909	4.135306	4.854175	5.567967	6.160594
Ag.2R.570.0_CDS_s_at	4746244	4748013	2R:4746144-4748008 (-) // 100.0 //	Up	AGAP001423	2R	ENSANGP00000015783	9.178818	10.62771	10.42478	10.50988	10.02296	0.874088	0.20293000000000028	0.48692000000000135	catalytic activity	0006164 // purine nucleotide biosynthetic process // inferred from electronic annotation /// 0006188 // IMP biosynthetic process // inferred from electronic annotation	---	0003824 // catalytic activity // inferred from electronic annotation /// 0003937 // IMP cyclohydrolase activity // inferred from electronic annotation /// 0004643 // phosphoribosylaminoimidazolecarboxamide formyltransferase activity // inferred from elect	-1.6508	0.804849	0.460911	0.605147	-0.22011	1.448891	1.245959	1.331062	0.84414	8.688312	7.471426	7.38966	5.061904	0.0000003101	0.000002	0.000002273	0.000141	6.508439	5.700521	5.643329	3.850645
Ag.2R.3645.0_at	5267623	5267877	2R:5267484-5268886 (-) // 100.0 //	Up	AGAP001455	2R	ENSANGP00000030629	9.210098	9.612041	9.667815	9.839121	9.719669	0.89286	-0.055773999999999546	0.11945200000000078	unknown	---	---	---	-1.67472	0.009606	0.243324	0.961173	0.460616	0.401943	0.457717	0.629023	0.509571	5.626648	6.407407	8.152264	7.13329	0.00004844	0.00001186	0.0000006882	0.000003455	4.314836	4.925944	6.162293	5.461533
Ag.2R.1164.0_CDS_at	7006343	7007019	2R:7006336-7007119 (-) // 100.0 //	Up	AGAP001626	2R	ENSANGP00000017409	5.996006	7.094167	7.567155	7.141031	7.217223	0.782174	-0.47298799999999996	-0.07619199999999982	binding	---	---	0005515 // protein binding // inferred from electronic annotation	-1.69896	0.153599	0.95151	0.232657	0.36119	1.098162	1.571149	1.145026	1.221218	4.736849	6.777052	4.572621	5.267644	0.000266	0.000006272	0.000367	0.000095	3.57565	5.202577	3.434744	4.021823
Ag.2R.1251.0_CDS_s_at	7656429	7657147	2R:7656372-7657278 (+) // 100.0 //	Down	AGAP001664 /// AGAP001665 /// AGAP001666 /// AGAP001667 /// AGAP001668 /// AGAP001669	2R	ENSANGP00000010127	8.060531	5.893948	5.815636	6.167879	6.150787	0.722872	0.07831200000000038	0.017091999999999885	structural molecule activity	---	---	0005198 // structural molecule activity // inferred from electronic annotation /// 0042302 // structural constituent of cuticle // inferred from electronic annotation	1.763942	-0.56244	-0.64653	-0.26831	-0.28666	-2.16658	-2.24489	-1.89265	-1.90974	-5.12954	-5.31495	-4.14859	-4.52146	0.000124	0.000087	0.00086	0.000407	3.907174	4.060854	3.065645	3.390596
Ag.2R.956.0_CDS_a_at	8788986	8790628	2R:8781984-8790785 (+) // 100.0 //	Up	AGAP001699	2R	ENSANGP00000012194	5.580378	6.380053	6.454506	6.62087	6.417709	0.787899	-0.0744530000000001	0.2031609999999997	catalytic activity	---	---	0016884 // carbon-nitrogen ligase activity, with glutamine as amido-N-donor // inferred from electronic annotation	-1.74275	0.219216	0.401881	0.810048	0.311602	0.799676	0.874128	1.040493	0.837331	5.106175	5.581577	6.151024	5.346617	0.00013	0.00005267	0.00001865	0.00008186	3.887673	4.278456	4.729281	4.086909
Ag.2R.289.0_CDS_a_at	10541398	10584693	2R:10541398-10584693 (+) // 100.0 // /// 2R:10541398-10584692 (+) // 99.96 //	Down	AGAP001797	2R		11.62744	10.92021	10.91438	10.85293	10.91373	0.843482	0.005830000000001334	-0.06079999999999863	binding/Tropomyosin	---	---	---	1.782505	-0.38466	-0.40251	-0.59082	-0.40451	-0.70723	-0.71305	-0.7745	-0.71371	-6.4598	-6.51301	-6.54952	-6.51896	0.00001082	0.000009867	0.000009263	0.000009766	4.965651	5.005806	5.033262	5.010289
Ag.2R.289.15_CDS_a_at	10557934	10581071	2R:10557934-10581071 (+) // 100.0 //	Down	AGAP001797	2R		11.5171	11.01423	11.01029	10.9333	10.95327	0.699855	0.003940000000000055	-0.01997000000000071	unknown	---	---	---	1.770071	-0.29296	-0.3091	-0.62497	-0.54304	-0.50287	-0.50681	-0.5838	-0.56383	-4.11507	-4.14727	-4.42294	-4.61391	0.00092	0.000862	0.000495	0.000339	3.036185	3.064487	3.305271	3.470291
Ag.2R.288.0_CDS_a_at	10593313	10595066	2R:10591412-10597580 (+) // 100.0 //	Down	AGAP001799	2R	ENSANGP00000024231	11.61492	10.8821	10.79942	10.65832	10.82759	0.816886	0.08267999999999986	-0.16927000000000092	binding	---	---	---	1.745416	-0.19714	-0.4163	-0.79034	-0.34164	-0.73282	-0.8155	-0.9566	-0.78733	-5.46042	-6.07648	-6.59913	-5.86662	0.000066	0.00002131	0.000008503	0.00003115	4.180079	4.671359	5.070442	4.506525
Ag.2R.2131.0_CDS_at	11429676	11451065	2R:11429676-11451065 (-) // 100.0 //	Up	AGAP001855	2R		5.203016	7.232765	7.06836	7.427751	7.205925	0.936754	0.16440499999999947	0.22182600000000008	unknown	---	---	---	-1.77129	0.441801	0.262547	0.6544	0.412538	2.029749	1.865344	2.224735	2.00291	11.50058	10.56906	11.67031	11.34851	0.000000007818	0.00000002431	0.000000006409	0.00000000936	8.106929	7.614215	8.193234	8.028724
Ag.2R.1515.2_a_at	14687325	14688464	2R:14687325-14688464 (+) // 100.0 //	Down	---	2R		7.430001	6.405659	6.332549	6.502383	6.438182	0.728299	0.07310999999999979	0.06420099999999973	unknown	---	---	---	1.772679	-0.47395	-0.6343	-0.26181	-0.40262	-1.02434	-1.09745	-0.92762	-0.99182	-4.94534	-5.29831	-4.14617	-4.78833	0.000177	0.00008971	0.000864	0.00024	3.752657	4.047137	3.063521	3.619555
Ag.2R.285.0_CDS_at	14826954	14831434	2R:14826954-14831434 (+) // 99.61 //	Up	---	2R		11.88867	12.2295	12.25248	12.2565	12.24415	0.769434	-0.022980000000000445	0.012350000000001415	unknown	---	---	---	-1.78512	0.345305	0.488925	0.514046	0.436841	0.340835	0.363812	0.367831	0.355479	4.514397	4.818731	4.51056	4.708363	0.000413	0.000226	0.000416	0.000281	3.384494	3.645424	3.381178	3.5513
Ag.2R.528.1_CDS_a_at	15138130	15143602	2R:15137271-15143753 (+) // 100.0 // /// 2R:15138084-15143698 (+) // 84.38 //	Down	AGAP002085	2R	ENSANGP00000019896	10.73324	9.396902	9.395414	9.486276	9.599588	0.911983	0.0014880000000001559	-0.11331200000000052	unknown	---	---	---	1.769592	-0.56955	-0.57216	-0.41311	-0.21477	-1.33634	-1.33783	-1.24697	-1.13366	-9.87643	-9.88742	-8.53226	-8.37845	0.00000005951	0.00000005865	0.0000003898	0.0000004896	7.225387	7.231718	6.4092	6.310165
Ag.2R.1384.0_CDS_at	15759803	15763147	2R:15758743-15763174 (-) // 100.0 //	Up	AGAP002114	2R	ENSANGP00000017686	4.506129	5.430733	5.658611	5.56882	5.32719	0.749789	-0.22787799999999958	0.2416299999999998	enzyme regulator activity	---	---	---	-1.71955	0.28748	0.782132	0.587223	0.062718	0.924605	1.152482	1.062692	0.821061	4.703828	5.863131	5.005288	4.17706	0.000284	0.00003135	0.000157	0.000812	3.547419	4.503761	3.80314	3.090635
Ag.2R.963.0_CDS_at	15977750	15978675	2R:15977667-15978676 (+) // 100.0 //	Up	AGAP002117	2R	ENSANGP00000025857	8.824153	9.41346	9.501341	9.748268	9.401011	0.831344	-0.08788099999999943	0.34725699999999904	binding	---	---	---	-1.63067	0.105512	0.36442	1.091901	0.068836	0.589308	0.677188	0.924115	0.576858	5.133521	5.899056	7.452912	5.025076	0.000123	0.00002936	0.000002053	0.000151	3.910493	4.532173	5.687604	3.819763
Ag.2R.1093.0_CDS_at	16605761	16606700	2R:16605760-16607511 (+) // 100.0 //	Down	AGAP002159	2R	ENSANGP00000028149	8.884382	8.319769	8.328802	8.454991	8.380944	0.868894	-0.009032999999998736	0.0740470000000002	unknown	---	---	---	1.741728	-0.65328	-0.61497	-0.07969	-0.39379	-0.56461	-0.55558	-0.42939	-0.50344	-7.19859	-7.08342	-5.06846	-6.41863	0.000003103	0.000003752	0.000139	0.00001163	5.508197	5.425728	3.856134	4.934461
Ag.2R.300.31_UTR_a_at	20520779	20530749	2R:20520779-20530749 (+) // 93.26 // /// 2R:20520779-20530749 (+) // 98.12 //	Down	AGAP002350	2Rb		12.24457	11.69394	11.55014	11.61871	11.71644	0.892277	0.14379999999999882	-0.09773000000000032	binding	---	---	---	1.73771	-0.25649	-0.77729	-0.52894	-0.17499	-0.55063	-0.69443	-0.62586	-0.52813	-6.82951	-8.61309	-7.18676	-6.55041	0.000005739	0.0000003461	0.000003164	0.000009248	5.241176	6.460754	5.499764	5.03393
Ag.2R.300.0_CDS_a_at	20522392	20529622	2R:20520779-20530779 (+) // 98.95 // /// 2R:20520779-20530779 (+) // 95.12 //	Down	AGAP002350	2Rb	ENSANGP00000024163	12.46907	11.894	11.79296	11.78411	11.97647	0.804819	0.10103999999999935	-0.19236000000000075	binding	---	---	---	1.717652	-0.31585	-0.67314	-0.70442	-0.02424	-0.57507	-0.67612	-0.68496	-0.49261	-5.31773	-6.25207	-5.86402	-4.55517	0.00008647	0.00001559	0.0000313	0.00038	4.063142	4.807263	4.504466	3.419696
Ag.2R.662.0_UTR_at	20538786	20542557	2R:20538786-20542547 (+) // 99.5 // /// 2R:20538786-20542557 (+) // 100.0 //	Up	AGAP002352	2Rb		8.938278	9.788355	9.825638	10.05505	9.62559	0.822515	-0.0372830000000004	0.42945999999999884	transcription regulator activity	0006355 // regulation of transcription, DNA-dependent // inferred from electronic annotation /// 0045449 // regulation of transcription // inferred from electronic annotation	0005634 // nucleus // inferred from electronic annotation	0003677 // DNA binding // inferred from electronic annotation /// 0003700 // transcription factor activity // inferred from electronic annotation	-1.66817	0.3339	0.421708	0.961999	-0.04944	0.850077	0.88736	1.116767	0.687312	5.840408	6.096561	7.103532	4.72214	0.00003268	0.00002056	0.000003629	0.000273	4.485752	4.686997	5.440184	3.563081
Ag.2R.662.0_CDS_at	20538905	20542137	2R:20538786-20542557 (+) // 100.0 // /// 2R:20538786-20542547 (+) // 99.5 //	Up	AGAP002352	2Rb	ENSANGP00000014785	8.283381	9.197678	9.327825	9.646026	9.160507	0.816456	-0.1301470000000009	0.48551900000000003	transcription regulator activity	0006355 // regulation of transcription, DNA-dependent // inferred from electronic annotation /// 0045449 // regulation of transcription // inferred from electronic annotation	0005634 // nucleus // inferred from electronic annotation	0003677 // DNA binding // inferred from electronic annotation /// 0003700 // transcription factor activity // inferred from electronic annotation	-1.65682	0.147182	0.403975	1.031819	0.07384	0.914296	1.044443	1.362645	0.877125	5.24801	5.995046	7.241307	5.034649	0.00009872	0.00002468	0.000002893	0.000149	4.005588	4.60771	5.53859	3.827797
Ag.2R.1390.0_UTR_at	20873298	20873710	2R:20872913-20874721 (-) // 100.0 //	Up	AGAP002386	2Rb	ENSANGP00000014850	7.837837	8.321388	8.320498	8.649003	8.271689	0.922456	0.0008900000000000574	0.37731400000000015	transcription regulator activity	0006457 // protein folding // inferred from electronic annotation	---	0031072 // heat shock protein binding // inferred from electronic annotation /// 0051082 // unfolded protein binding // inferred from electronic annotation	-1.52796	0.142709	0.139632	1.274616	-0.029	0.483551	0.482661	0.811166	0.433852	6.815095	6.802545	10.58439	6.114646	0.00000588	0.000006	0.00000002385	0.00002	5.230586	5.221356	7.622598	4.701058
Ag.2R.2584.0_CDS_at	20970024	20972749	2R:20970023-20972749 (+) // 100.0 //	Up	AGAP002404	2Rb	ENSANGP00000014259	6.67876	7.310389	7.684282	7.477582	7.395005	0.843886	-0.3738929999999998	0.08257699999999968	ubiquitin protein ligase binding	0006511 // ubiquitin-dependent protein catabolic process // inferred from electronic annotation	0031461 // cullin-RING ubiquitin ligase complex // inferred from electronic annotation	0031625 // ubiquitin protein ligase binding // inferred from electronic annotation	-1.66446	0.00313	0.990262	0.444543	0.226528	0.631629	1.005523	0.798822	0.716245	5.046729	8.034143	5.909142	5.72281	0.000145	0.0000008242	0.00002883	0.00004055	3.837929	6.083992	4.540136	4.392057
Ag.2R.1393.0_CDS_at	21565121	21571825	2R:21568980-21571872 (-) // 96.89 //	Down	AGAP002456	2Rb	ENSANGP00000021340	8.598377	7.054366	7.285877	6.694621	7.197427	0.819734	-0.23151100000000024	-0.5028060000000005	binding	---	---	---	1.700069	-0.43013	-0.11073	-0.92645	-0.23276	-1.54401	-1.3125	-1.90376	-1.40095	-6.30935	-5.36332	-7.20231	-5.72475	0.000014	0.00007932	0.000003084	0.0000404	4.851192	4.100627	5.51085	4.393612
Ag.2R.3248.0_CDS_at	21868964	21869363	2R:21869099-21869623 (-) // 95.56 //	Up	---	2Rb	ENSANGP00000024914	6.679818	7.600339	7.618735	8.014465	7.544095	0.836948	-0.01839600000000008	0.47036999999999907	unknown	---	---	---	-1.65413	0.221826	0.259316	1.065786	0.107205	0.920521	0.938917	1.334647	0.864277	5.83906	5.955752	7.837946	5.482295	0.00003276	0.00002649	0.000001116	0.0000634	4.484682	4.576855	5.952272	4.197905
Ag.2R.335.0_CDS_at	22045958	22048654	2R:22045703-22048873 (-) // 100.0 //	Up	AGAP002499	2Rb	ENSANGP00000022164	11.17474	11.62508	11.52909	11.82167	11.58051	0.834332	0.09599000000000046	0.24115999999999893	catalytic activity	0006573 // valine metabolic process // inferred from electronic annotation /// 0008152 // metabolic process // inferred from electronic annotation /// 0055114 // oxidation reduction // inferred from electronic annotation	0005739 // mitochondrion // inferred from electronic annotation	0004491 // methylmalonate-semialdehyde dehydrogenase (acylating) activity // inferred from electronic annotation /// 0016491 // oxidoreductase activity // inferred from electronic annotation /// 0018478 // malonate-semialdehyde dehydrogenase (acetylating)	-1.57841	0.335065	-0.07278	1.170398	0.145724	0.450339	0.354352	0.646936	0.405777	5.375033	4.229374	7.148743	4.843164	0.00007758	0.00073	0.000003368	0.000216	4.110243	3.136487	5.472597	3.666181
Ag.2R.378.11_at	26384338	26385195	2R:26384338-26385195 (+) // 100.0 //	Down	---	2Rb		7.112094	6.410346	6.117195	6.209844	6.504329	0.857972	0.29315099999999994	-0.2944849999999999	unknown	---	---	---	1.643401	-0.15481	-0.90601	-0.6686	0.086016	-0.70175	-0.9949	-0.90225	-0.60776	-5.77961	-8.194	-6.87972	-5.00556	0.00003652	0.000000646	0.000005273	0.000157	4.437414	6.189783	5.277973	3.803369
Ag.2R.365.0_CDS_a_at	28075093	28081793	2R:28074843-28082001 (-) // 100.0 //	Up	AGAP002830	2Rc	ENSANGP00000016878	7.632622	9.90447	8.952654	9.615682	8.763899	0.918243	0.9518159999999991	0.8517829999999993	catalytic activity	0008152 // metabolic process // inferred from electronic annotation /// 0009396 // folic acid and derivative biosynthetic process // inferred from electronic annotation	---	0003824 // catalytic activity // inferred from electronic annotation /// 0004329 // formate-tetrahydrofolate ligase activity // inferred from electronic annotation /// 0005488 // binding // inferred from electronic annotation /// 0005524 // ATP binding //	-1.51831	1.053462	-0.02401	0.726549	-0.23769	2.271847	1.320031	1.98306	1.131277	11.73334	6.817528	9.482111	5.842672	0.000000005956	0.000005856	0.0000001012	0.00003254	8.225027	5.232375	6.994743	4.487547
Ag.2R.1108.0_CDS_at	28300520	28301010	2R:28300453-28301118 (+) // 100.0 //	Down	AGAP002848	2Rc	ENSANGP00000017011	9.465614	8.794689	8.841482	8.337064	8.603123	0.811109	-0.046792999999999196	-0.26605900000000027	binding	---	---	---	1.573858	-0.03282	0.079235	-1.12871	-0.49157	-0.67092	-0.62413	-1.12855	-0.86249	-4.57102	-4.25222	-7.11847	-5.87616	0.000369	0.000697	0.000003541	0.00003061	3.433367	3.156483	5.45091	4.514076
Ag.2R.769.0_CDS_at	29819156	29819519	2R:29818617-29819712 (-) // 100.0 //	Up	AGAP002929	2Rc	ENSANGP00000010971	8.46063	8.782464	9.001455	8.893381	8.852233	0.830035	-0.21899100000000082	0.04114799999999974	binding	---	---	---	-1.64885	-0.07608	0.994104	0.465957	0.264869	0.321834	0.540825	0.432751	0.391603	4.09092	6.874579	5.092767	4.977768	0.000966	0.000005318	0.000133	0.000166	3.014938	5.274211	3.87647	3.779987
Ag.2R.473.0_CDS_at	29851787	29852210	2R:29851709-29852735 (+) // 99.57 //	Down	AGAP002936	2Rc	ENSANGP00000011063	9.021249	7.21905	6.716295	6.667008	7.043102	0.798764	0.5027550000000005	-0.37609400000000015	binding	---	---	---	1.738385	-0.11771	-0.6355	-0.68626	-0.29892	-1.8022	-2.30495	-2.35424	-1.97815	-5.10594	-6.53033	-6.17519	-5.60443	0.00013	0.000009575	0.00001787	0.00005048	3.887478	5.018844	4.747989	4.296921
Ag.2R.351.0_CDS_at	30716628	30717304	2R:30716521-30717472 (-) // 100.0 //	Down	AGAP002994	2Rc	ENSANGP00000018253	12.06792	10.7988	10.90878	10.63255	10.7339	0.82452	-0.10998000000000019	-0.10135000000000005	structural molecule activity	---	---	0042302 // structural constituent of cuticle // inferred from electronic annotation	1.762767	-0.38932	-0.20282	-0.67124	-0.49938	-1.26912	-1.15913	-1.43537	-1.33402	-6.34337	-5.79364	-6.64213	-6.66778	0.00001327	0.0000356	0.000007897	0.000007557	4.877194	4.448592	5.102548	5.121645
Ag.2R.591.0_CDS_a_at	31240601	31243274	2R:31240394-31243767 (+) // 100.0 // /// 2R:31240394-31243676 (+) // 95.01 //	Down	AGAP003027	2Rc	ENSANGP00000018251	10.24209	9.306599	8.982535	9.109552	9.051404	0.845766	0.3240639999999999	0.05814800000000098	transcription regulator activity	---	---	---	1.739852	-0.0613	-0.68524	-0.44068	-0.55264	-0.93549	-1.25955	-1.13253	-1.19068	-5.62997	-7.58026	-6.31024	-7.16579	0.00004814	0.000001675	0.000014	0.000003275	4.317515	5.776059	4.851877	5.48479
Ag.2R.1411.0_CDS_a_at	31252368	31253909	2R:31252304-31253772 (-) // 100.0 //	Down	AGAP003030	2Rc	ENSANGP00000018271	10.75271	9.647328	9.816969	9.404881	9.854843	0.898779	-0.16964100000000037	-0.4499620000000011	catalytic activity	0008152 // metabolic process // inferred from electronic annotation	---	0016624 // oxidoreductase activity, acting on the aldehyde or oxo group of donors, disulfide as acceptor // inferred from electronic annotation	1.677462	-0.48526	-0.15335	-0.95961	-0.07925	-1.10538	-0.93574	-1.34783	-0.89787	-8.62252	-7.29924	-9.73381	-7.0038	0.0000003414	0.000002632	0.00000007198	0.000004283	6.466746	5.579641	7.142771	5.368265
Ag.2R.1718.0_CDS_a_at	31276970	31291392	2R:31283505-31291682 (-) // 100.0 // /// 2R:31283542-31291682 (-) // 94.77 //	Down	AGAP003037	2Rc	ENSANGP00000031939	8.041295	5.627755	5.61754	4.344125	5.602024	0.944369	0.010214999999999641	-1.257899	binding	---	---	---	1.631986	-0.16269	-0.17029	-1.11718	-0.18183	-2.41354	-2.42376	-3.69717	-2.43927	-10.3961	-10.4401	-14.7439	-10.5069	0.00000003027	0.00000002862	0.000000000252	0.00000002629	7.51899	7.54333	9.598727	7.58015
Ag.2R.3684.0_at	32391613	32392649	2R:32391613-32392649 (-) // 100.0 //	Up	---	2Rd/2Ru		8.018427	9.105456	9.336849	8.991984	8.827854	0.78856	-0.23139300000000063	0.1641300000000001	unknown	---	---	---	-1.66356	0.495166	0.954689	0.269824	-0.05612	1.087029	1.318423	0.973558	0.809428	5.590436	6.780459	4.63546	4.162772	0.00005181	0.000006236	0.000324	0.000835	4.285617	5.205089	3.488807	3.078096
Ag.2R.847.0_CDS_at	33620457	33622184	2R:33620003-33622184 (-) // 100.0 //	Down	AGAP003184	2Rd/2Ru	ENSANGP00000014729	7.751288	6.709793	6.749509	6.71783	6.673223	0.793957	-0.03971599999999942	0.04460700000000006	catalytic activity	0009058 // biosynthetic process // inferred from electronic annotation /// 0033014 // tetrapyrrole biosynthetic process // inferred from electronic annotation	---	0003824 // catalytic activity // inferred from electronic annotation /// 0003870 // 5-aminolevulinate synthase activity // inferred from electronic annotation /// 0016769 // transferase activity, transferring nitrogenous groups // inferred from electronic	1.785807	-0.45246	-0.36711	-0.43519	-0.53105	-1.04149	-1.00178	-1.03346	-1.07806	-5.8862	-5.66174	-5.40751	-6.09288	0.00003	0.00004539	0.000073	0.0000207	4.522012	4.343076	4.136852	4.684133
Ag.2R.1575.1_CDS_a_at	34561813	34566923	2R:34564235-34566818 (+) // 99.94 //	Down	AGAP003261	2Rd/2Ru	ENSANGP00000004103	9.446264	7.202722	7.617098	6.237903	7.332409	0.877467	-0.41437600000000074	-1.094506	structural molecule activity	---	---	---	1.6041	-0.31122	0.04253	-1.1349	-0.20051	-2.24354	-1.82917	-3.20836	-2.11386	-7.26662	-5.9245	-9.62074	-6.84658	0.000002776	0.000028	0.00000008383	0.000005576	5.556553	4.552248	7.076624	5.253703
Ag.2R.2762.0_CDS_s_at	35084574	35085003	2R:35084574-35085003 (+) // 91.47 //	Down	AGAP003289	2Rd/2Ru		9.709098	8.44038	8.671882	8.148047	9.073645	0.903982	-0.23150200000000076	-0.9255980000000008	catalytic activity	0006730 // one-carbon compound metabolic process // inferred from electronic annotation	---	0004089 // carbonate dehydratase activity // inferred from electronic annotation /// 0008270 // zinc ion binding // inferred from electronic annotation	1.484534	-0.60706	-0.22541	-1.089	0.436933	-1.26872	-1.03722	-1.56105	-0.63545	-8.7723	-7.17162	-9.9929	-4.39371	0.0000002746	0.000003244	0.00000005103	0.000525	6.56134	5.488955	7.292196	3.279875
Ag.2R.442.0_UTR_at	35613943	35628369	2R:35613943-35628369 (+) // 97.86 //	Down	AGAP003308	2Rd		9.877563	6.8156	7.134723	5.769459	6.853391	0.955951	-0.31912300000000027	-1.083932	chitin binding	0006030 // chitin metabolic process // inferred from electronic annotation	0005576 // extracellular region // inferred from electronic annotation	0008061 // chitin binding // inferred from electronic annotation	1.683612	-0.30878	-0.10113	-0.9895	-0.28419	-3.06196	-2.74284	-4.1081	-3.02417	-12.9335	-11.5856	-16.0652	-12.7739	0.000000001567	0.000000007075	0.0000000000748	0.00000000186	8.804985	8.150284	10.12582	8.730512
Ag.2R.441.1_UTR_s_at	35614272	35628196	2R:35614272-35628196 (+) // 100.0 //	Down	AGAP003308	2Rd		10.01646	6.564929	6.8063	5.519602	6.843969	0.959285	-0.241371	-1.3243670000000005	chitin binding	0006030 // chitin metabolic process // inferred from electronic annotation	0005576 // extracellular region // inferred from electronic annotation	0008061 // chitin binding // inferred from electronic annotation	1.695649	-0.34628	-0.20348	-0.96469	-0.1812	-3.45154	-3.21016	-4.49686	-3.1725	-13.8095	-12.8438	-16.6572	-12.6931	0.000000000631	0.000000001725	0.0000000000447	0.00000000203	9.200276	8.763214	10.34953	8.692504
Ag.2R.442.1_CDS_s_at	35625294	35627484	2R:35613943-35629117 (+) // 98.83 //	Down	AGAP003308	2Rd	ENSANGP00000025214	10.64732	7.73141	7.493844	6.866199	7.431761	0.948417	0.23756600000000017	-0.5655619999999999	chitin binding	0006030 // chitin metabolic process // inferred from electronic annotation	0005576 // extracellular region // inferred from electronic annotation	0008061 // chitin binding // inferred from electronic annotation	1.748055	-0.20248	-0.3614	-0.78125	-0.40293	-2.91591	-3.15348	-3.78112	-3.21556	-11.5282	-12.4675	-13.84	-12.7129	0.000000007567	0.000000002598	0.000000000611	0.000000001987	8.121061	8.585314	9.21363	8.701842
Ag.2R.1304.0_CDS_at	37297285	37298816	2R:37297278-37299078 (+) // 100.0 //	Up	AGAP003397	2Rd	ENSANGP00000014950	9.906804	10.40045	10.35081	10.47841	10.31381	0.764926	0.04964000000000013	0.16460000000000008	catalytic activity	---	---	0003676 // nucleic acid binding // inferred from electronic annotation /// 0004386 // helicase activity // inferred from electronic annotation /// 0005524 // ATP binding // inferred from electronic annotation /// 0008026 // ATP-dependent helicase activity	-1.71912	0.4952	0.272523	0.844859	0.106538	0.49365	0.444008	0.571601	0.407004	5.078411	4.567715	5.444131	4.187038	0.000137	0.000371	0.00006811	0.000795	3.864464	3.430516	4.16679	3.099388
Ag.2R.304.11_at	37743337	37761185	2R:37743337-37761185 (-) // 100.0 //	Up	AGAP003439	2Rd		7.854013	8.401331	8.60756	8.518431	8.429627	0.820422	-0.20622899999999866	0.08880399999999966	binding	---	---	0005515 // protein binding // inferred from electronic annotation	-1.72056	0.132512	0.830752	0.528985	0.228315	0.547318	0.753548	0.664419	0.575614	5.063288	6.971138	5.690645	5.325057	0.000141	0.000004523	0.000043	0.00008528	3.851805	5.344583	4.366288	4.069175
Ag.2R.5132.0_at	37747534	37747828	2R:37747534-37747828 (-) // 100.0 //	Up	---	2Rd		8.019348	8.518584	8.625464	8.736227	8.690755	0.784847	-0.10687999999999853	0.04547200000000018	unknown	---	---	---	-1.71651	0.00175	0.369608	0.75083	0.594325	0.499236	0.606117	0.71688	0.671408	4.284818	5.202144	5.69638	5.762519	0.000653	0.000108	0.00004257	0.00003769	3.184977	3.967578	4.370887	4.423786
Ag.2R.1307.1_at	38595723	38596962	2R:38595723-38596962 (+) // 100.0 // /// 2R:38595723-38596962 (+) // 97.83 //	Up	---	2Rd		8.952406	9.47012	9.62688	9.442492	9.409644	0.767	-0.15676000000000023	0.032847999999999544	unknown	---	---	---	-1.68915	0.354534	0.973345	0.24547	0.115804	0.517714	0.674474	0.490086	0.457238	4.717293	6.145647	4.134295	4.166249	0.000276	0.00001883	0.000885	0.00083	3.558937	4.725114	3.053085	3.081148
Ag.2R.1309.1_CDS_s_at	39134630	39140616	2R:39134629-39140656 (+) // 99.4 //	Up	AGAP003525	2Rd	ENSANGP00000026958	9.806934	10.51151	10.3357	10.88207	10.30346	0.926742	0.17581000000000024	0.5786100000000012	transcription regulator activity	0045449 // regulation of transcription // inferred from electronic annotation	0005634 // nucleus // inferred from electronic annotation	0030528 // transcription regulator activity // inferred from electronic annotation	-1.44266	0.369208	-0.0829	1.322142	-0.1658	0.704574	0.528766	1.075138	0.496529	8.232421	6.178238	11.63033	5.801576	0.0000006095	0.00001777	0.000000006714	0.000035	6.215005	4.750343	8.172996	4.454903
Ag.2R.899.0_CDS_a_at	41060161	41061945	2R:41060332-41062114 (+) // 99.76 //	Up	AGAP003652	2Rd	ENSANGP00000020207	9.283818	9.844836	9.733811	9.84261	9.84378	0.774108	0.11102500000000148	-0.0011700000000001154	catalytic activity	0008152 // metabolic process // inferred from electronic annotation /// 0055114 // oxidation reduction // inferred from electronic annotation	---	0016491 // oxidoreductase activity // inferred from electronic annotation	-1.75414	0.556218	0.098999	0.547051	0.55187	0.561018	0.449993	0.558792	0.559962	5.427816	4.353651	5.005243	5.417601	0.00007023	0.000569	0.000157	0.0000716	4.153463	3.245018	3.803101	4.145111
Ag.2R.2003.0_CDS_at	42368200	42373400	2R:42368774-42371219 (+) // 100.0 //	Down	AGAP003714	2Rd	ENSANGP00000019620	7.278226	4.898588	6.046192	4.20215	5.780766	0.932158	-1.1476039999999994	-1.5786160000000002	catalytic activity	0006979 // response to oxidative stress // inferred from electronic annotation /// 0055114 // oxidation reduction // inferred from electronic annotation	---	0004601 // peroxidase activity // inferred from electronic annotation /// 0016491 // oxidoreductase activity // inferred from electronic annotation /// 0020037 // heme binding // inferred from electronic annotation	1.397894	-0.63411	0.345842	-1.22881	0.119191	-2.37964	-1.23203	-3.07608	-1.49746	-10.6762	-5.5275	-12.777	-6.71833	0.00000002125	0.00005826	0.000000001854	0.000006932	7.672595	4.234652	8.731968	5.159172
Ag.2R.2004.0_CDS_at	42372465	42373365	2R:42372358-42373450 (+) // 100.0 //	Down	---	2Rd	ENSANGP00000021348	11.99998	9.542044	11.10048	8.706099	10.8696	0.951224	-1.5584359999999986	-2.163501	catalytic activity	---	---	---	1.187819	-0.68811	0.501311	-1.32612	0.3251	-2.45794	-0.8995	-3.29388	-1.13038	-11.8195	-4.3254	-14.6643	-5.43563	0.000000005392	0.000602	0.000000000272	0.00006921	8.268231	3.220395	9.565663	4.159844
Ag.2R.1128.0_CDS_a_at	42668057	42668931	2R:42667960-42669219 (+) // 100.0 //	Down	AGAP003734	2Rd	ENSANGP00000015314	9.278098	7.902592	8.441285	7.055864	8.361616	0.886645	-0.5386930000000003	-1.305752	binding	---	---	---	1.316381	-0.37553	0.287081	-1.41702	0.189086	-1.37551	-0.83681	-2.22223	-0.91648	-6.80651	-4.14086	-10.1807	-4.53509	0.000005967	0.000873	0.00000003993	0.000396	5.224273	3.058849	7.398694	3.402368
Ag.2R.2009.0_CDS_at	42870660	42874111	2R:42870659-42874205 (+) // 99.96 // /// 2R:42870659-42874205 (+) // 98.49 //	Down	AGAP003746	2Rd	ENSANGP00000015235	7.011596	5.816675	5.820088	5.949939	5.872943	0.785684	-0.0034130000000001104	0.0769959999999994	unknown	---	---	---	1.779047	-0.53831	-0.53169	-0.27986	-0.42919	-1.19492	-1.19151	-1.06166	-1.13865	-5.95926	-5.94224	-4.9019	-5.67864	0.00002633	0.00002715	0.000192	0.000044	4.579613	4.566223	3.715951	4.356658
Ag.2R.2291.0_CDS_at	43097946	43098784	2R:43097946-43098784 (-) // 100.0 // /// 2R:43095032-43095655 (-) // 91.67 //	Down	AGAP003760	2Rd		6.51674	5.384054	5.28886	4.950176	5.238467	0.912015	0.09519400000000022	-0.2882910000000001	catalytic activity	---	---	---	1.723488	-0.15165	-0.30924	-0.86993	-0.39267	-1.13269	-1.22788	-1.56656	-1.27827	-8.10354	-8.78458	-10.3762	-9.14511	0.0000007412	0.0000002697	0.00000003105	0.0000001614	6.130081	6.569049	7.507981	6.791977
Ag.2R.353.0_CDS_at	43128616	43133832	2R:43127767-43134185 (-) // 100.0 //	Up	AGAP003769	2Rd	ENSANGP00000015236	9.643317	10.18245	10.06337	10.54913	10.05529	0.855919	0.11907999999999852	0.4938400000000005	binding	---	---	0005488 // binding // inferred from electronic annotation	-1.4047	0.258305	-0.10902	1.389343	-0.13393	0.539135	0.420051	0.905809	0.411975	5.412039	4.216634	8.418352	4.135567	0.00007235	0.000749	0.0000004613	0.000883	4.140561	3.12533	6.335975	3.054202
Ag.2R.2883.0_CDS_at	44347602	44349739	2R:44347601-44349739 (+) // 100.0 //	Up	AGAP003846	2R	ENSANGP00000018874	4.424472	5.639259	5.454841	6.018545	5.410706	0.831762	0.18441799999999997	0.6078389999999994	catalytic activity	0006644 // phospholipid metabolic process // inferred from electronic annotation /// 0016042 // lipid catabolic process // inferred from electronic annotation	---	0004623 // phospholipase A2 activity // inferred from electronic annotation /// 0005509 // calcium ion binding // inferred from electronic annotation	-1.63477	0.422956	0.110571	1.065428	0.035812	1.214786	1.030368	1.594073	0.986234	6.357714	5.392543	7.723885	5.161561	0.000013	0.000075	0.000001334	0.000116	4.888134	4.124597	5.874728	3.933849
Ag.2R.629.0_UTR_a_at	44668717	44674617	2R:44668717-44674617 (+) // 100.0 // /// UNKN:25040606-25043406 (+) // 79.64 // /// UNKN:25040606-25043406 (+) // 79.99 //	Down	---	2R		12.12828	11.6414	11.70602	11.50145	11.42818	0.8452	-0.06461999999999968	0.07327000000000083	unknown	---	---	---	1.637338	-0.14522	0.091365	-0.65761	-0.92587	-0.48688	-0.42226	-0.62683	-0.7001	-5.20064	-4.51039	-6.19885	-7.47819	0.000108	0.000416	0.00001713	0.000001971	3.966326	3.381031	4.766267	5.705236
Ag.2R.1323.0_CDS_at	46361954	46363930	2R:46361932-46364203 (+) // 100.0 //	Down	AGAP003926	2R	ENSANGP00000002729	7.242885	5.89521	6.234265	5.547094	5.939531	0.790626	-0.3390550000000001	-0.39243699999999926	catalytic activity	0006508 // proteolysis // inferred from electronic annotation	---	0008237 // metallopeptidase activity // inferred from electronic annotation /// 0008270 // zinc ion binding // inferred from electronic annotation	1.656756	-0.42782	0.096625	-0.96629	-0.35927	-1.34767	-1.00862	-1.69579	-1.30335	-5.63732	-4.21906	-6.5673	-5.45193	0.00004749	0.000746	0.000008983	0.00006712	4.323434	3.127451	5.046601	4.173152
Ag.2R.354.0_UTR_at	46558431	46565027	2R:46558431-46565027 (-) // 100.0 // /// 2R:46558432-46565027 (-) // 99.97 //	Down	---	2R		10.78817	10.13267	9.645199	10.00824	9.704785	0.864477	0.4874709999999993	0.30345500000000136	unknown	---	---	---	1.601626	0.168075	-0.89799	-0.10403	-0.76768	-0.6555	-1.14297	-0.77992	-1.08338	-4.73475	-8.25577	-5.21557	-7.82537	0.000267	0.0000005884	0.000105	0.000001138	3.573855	6.230294	3.978713	5.943758
Ag.2R.2314.0_CDS_at	48711824	48715293	2R:48706019-48712015 (-) // 100.0 //	Down	AGAP004050	2R	ENSANGP00000031393	8.502965	7.468589	7.851929	7.320967	7.971167	0.875286	-0.38334000000000046	-0.6502000000000008	binding	0006355 // regulation of transcription, DNA-dependent // inferred from electronic annotation /// 0007548 // sex differentiation // inferred from electronic annotation	0005634 // nucleus // inferred from electronic annotation	0003677 // DNA binding // inferred from electronic annotation /// 0003700 // transcription factor activity // inferred from electronic annotation	1.463814	-0.76337	0.062023	-1.08123	0.318763	-1.03438	-0.65104	-1.182	-0.5318	-7.97439	-5.01908	-8.4365	-4.09983	0.0000009035	0.000153	0.0000004491	0.000949	6.044095	3.814728	6.347684	3.022777
Ag.2R.2918.0_CDS_s_at	49723578	49725265	2R:49723465-49725265 (+) // 100.0 //	Down	AGAP004095	2R	ENSANGP00000020378	9.045659	7.871722	7.583475	8.101663	7.672335	0.841632	0.28824700000000014	0.42932799999999993	transporter activity	0006810 // transport // inferred from electronic annotation	0016020 // membrane // inferred from electronic annotation /// 0016021 // integral to membrane // inferred from electronic annotation	0005488 // binding // inferred from electronic annotation	1.68304	-0.31131	-0.80101	0.079324	-0.65004	-1.17394	-1.46218	-0.944	-1.37332	-6.14657	-7.6558	-4.57599	-7.19054	0.0000188	0.000001486	0.000365	0.000003144	4.725831	5.828095	3.437645	5.502456
Ag.2R.942.0_CDS_at	50617104	50618438	2R:50616511-50621276 (-) // 100.0 // /// 2R:50616532-50621276 (-) // 98.93 //	Down	AGAP004135	2R	ENSANGP00000011250	6.493228	4.874321	4.732562	4.822286	4.816777	0.907243	0.1417590000000004	0.005508999999999986	catalytic activity	---	---	---	1.784788	-0.36284	-0.5509	-0.43187	-0.43918	-1.61891	-1.76067	-1.67094	-1.67645	-9.00519	-9.79373	-8.60517	-9.32528	0.0000001967	0.00000006644	0.0000003502	0.0000001256	6.706214	7.177591	6.455717	6.901045
Ag.2R.363.0_CDS_a_at	50948265	50950104	2R:50947513-50952802 (-) // 100.0 //	Down	AGAP004159	2R	ENSANGP00000011712	10.82268	10.26116	10.18403	10.33549	10.2975	0.776211	0.07713000000000036	0.037990000000000634	catalytic activity	0006108 // malate metabolic process // inferred from electronic annotation /// 0008152 // metabolic process // inferred from electronic annotation /// 0055114 // oxidation reduction // inferred from electronic annotation	---	0003824 // catalytic activity // inferred from electronic annotation /// 0004470 // malic enzyme activity // inferred from electronic annotation /// 0005488 // binding // inferred from electronic annotation /// 0016491 // oxidoreductase activity // inferr	1.744811	-0.46926	-0.77338	-0.17618	-0.32599	-0.56152	-0.63865	-0.48719	-0.52519	-5.22301	-5.94045	-4.19549	-4.88503	0.000104	0.00002724	0.000782	0.000199	3.984883	4.564812	3.106799	3.701675
Ag.2R.2949.0_CDS_at	53226086	53226349	2R:53225864-53235430 (+) // 100.0 //	Down	---	2R	ENSANGP00000026895	7.457472	5.650421	6.474971	5.678629	6.457005	0.926248	-0.8245500000000003	-0.7783759999999997	unknown	---	---	---	1.50403	-0.9362	0.177268	-0.89811	0.153006	-1.80705	-0.9825	-1.77884	-1.00047	-11.7243	-6.37456	-10.6852	-6.49113	0.000000006019	0.00001256	0.00000002102	0.00001025	8.220483	4.90097	7.67746	4.989313
Ag.2R.2952.1_a_at	53441676	53449438	2R:53441676-53449438 (+) // 100.0 // /// 2R:53441676-53449438 (+) // 99.51 //	Up	AGAP004261	2R		8.633585	9.434442	8.991311	9.55616	9.256508	0.91872	0.44313100000000105	0.29965200000000003	DNA repair	0006281 // DNA repair // inferred from electronic annotation	---	0003913 // DNA photolyase activity // inferred from electronic annotation	-1.46366	0.703773	-0.49551	1.03319	0.222214	0.800857	0.357727	0.922576	0.622924	9.142805	4.083906	9.751084	7.111466	0.000000162	0.00098	0.00000007034	0.000003582	6.790574	3.008765	7.152824	5.445881
Ag.2R.31.0_CDS_at	54712520	54713599	2R:54712418-54713706 (-) // 100.0 //	Down	AGAP004334	2R	ENSANGP00000003578	10.00967	9.318806	9.262923	8.625351	8.862163	0.838155	0.05588299999999968	-0.23681200000000047	binding	---	---	---	1.502486	0.194981	0.089219	-1.11744	-0.66925	-0.69086	-0.74674	-1.38431	-1.1475	-4.12138	-4.45475	-7.64565	-6.84552	0.000908	0.000465	0.00000151	0.000005586	3.041735	3.332867	5.821123	5.252928
Ag.2R.381.0_CDS_at	55076751	55078560	2R:55075432-55081932 (-) // 100.0 //	Down	AGAP004366	2R	ENSANGP00000013138	9.618562	8.560266	8.518902	8.398002	8.216319	0.880489	0.041363999999999734	0.1816829999999996	catalytic activity	0006561 // proline biosynthetic process // inferred from electronic annotation /// 0008152 // metabolic process // inferred from electronic annotation	0005759 // mitochondrial matrix // inferred from electronic annotation	0003842 // 1-pyrroline-5-carboxylate dehydrogenase activity // inferred from electronic annotation /// 0016491 // oxidoreductase activity // inferred from electronic annotation	1.735475	-0.1854	-0.26048	-0.47992	-0.80968	-1.0583	-1.09966	-1.22056	-1.40224	-6.92665	-7.19738	-7.39608	-9.17781	0.000004873	0.000003109	0.00000225	0.0000001542	5.312227	5.507333	5.647831	6.811887
Ag.2R.314.2_UTR_a_at	55996971	56006074	2R:55996971-56006074 (+) // 100.0 // /// 2R:55996971-56006034 (+) // 98.84 //	Down	---	2R		9.357914	8.721445	8.853566	8.638357	8.900882	0.809083	-0.1321210000000015	-0.2625250000000001	unknown	---	---	---	1.659459	-0.61937	-0.14632	-0.91686	0.023092	-0.63647	-0.50435	-0.71956	-0.45703	-6.07913	-4.8172	-6.36292	-4.36527	0.00002121	0.000227	0.00001282	0.000556	4.673429	3.644123	4.892103	3.255131
Ag.2R.509.0_CDS_at	56365053	56366283	2R:56364922-56366536 (+) // 100.0 //	Up	AGAP004451	2R	ENSANGP00000017622	11.11114	12.11948	11.68804	11.85261	11.68989	0.906116	0.4314399999999985	0.1627200000000002	catalytic activity	0005975 // carbohydrate metabolic process // inferred from electronic annotation /// 0006012 // galactose metabolic process // inferred from electronic annotation	---	0003824 // catalytic activity // inferred from electronic annotation /// 0008108 // UDP-glucose:hexose-1-phosphate uridylyltransferase activity // inferred from electronic annotation /// 0016740 // transferase activity // inferred from electronic annotati	-1.57289	1.156463	-0.01134	0.434106	-0.00634	1.008342	0.576904	0.741471	0.578751	10.55066	6.036358	7.182781	6.055689	0.00000002488	0.000023	0.000003185	0.00002212	7.604141	4.640051	5.496922	4.655149
Ag.2R.1298.0_CDS_at	.	.	---	Up	---	2R	ENSANGT00000025542	7.232795	8.024734	8.227166	7.972801	7.984162	0.787062	-0.20243199999999995	-0.011361000000000843	unknown	---	---	---	-1.72232	0.358376	0.890238	0.22193	0.251779	0.791938	0.994371	0.740006	0.751366	5.275214	6.623647	4.563629	5.004959	0.00009374	0.000008152	0.000374	0.000157	4.028078	5.08876	3.426993	3.802863
Ag.3L.1690.0_CDS_at	2267563	2270082	3L:2267568-2271880 (+) // 100.0 //	Down	AGAP010367	3L	ENSANGP00000015986	6.701127	5.02066	5.367011	3.301635	5.044322	0.879087	-0.3463509999999994	-1.742687	catalytic activity	---	0005794 // Golgi apparatus // inferred from electronic annotation /// 0016020 // membrane // inferred from electronic annotation /// 0016021 // integral to membrane // inferred from electronic annotation	0005509 // calcium ion binding // inferred from electronic annotation /// 0005529 // sugar binding // inferred from electronic annotation /// 0016740 // transferase activity // inferred from electronic annotation /// 0016757 // transferase activity, trans	1.331626	-0.05469	0.231037	-1.47281	-0.03517	-1.68047	-1.33412	-3.39949	-1.65681	-5.47526	-4.34679	-10.2545	-5.39817	0.00006424	0.000577	0.0000000363	0.00007427	4.192175	3.239039	7.440135	4.129204
Ag.3L.165.0_CDS_a_at	3189394	3192278	3L:3189292-3193601 (+) // 99.84 //	Down	AGAP010429	3L	ENSANGP00000010243	12.03493	11.44295	11.43611	11.41454	11.50561	0.735182	0.006840000000000401	-0.0910700000000002	catalytic activity	0006099 // tricarboxylic acid cycle // inferred from electronic annotation	---	0009055 // electron carrier activity // inferred from electronic annotation /// 0016491 // oxidoreductase activity // inferred from electronic annotation /// 0016627 // oxidoreductase activity, acting on the CH-CH group of donors // inferred from electron	1.774046	-0.46948	-0.49542	-0.57713	-0.23202	-0.59198	-0.59883	-0.62039	-0.52932	-4.87026	-4.92655	-4.72533	-4.35476	0.000205	0.000183	0.000272	0.000568	3.689163	3.736792	3.565805	3.245984
Ag.3L.590.0_CDS_a_at	5054374	5055589	3L:5054458-5055696 (+) // 100.0 // /// 3L:5054458-5055696 (+) // 97.94 //	Up	AGAP010499	3L	ENSANGP00000020590	9.745711	10.56996	10.35765	10.29055	10.3154	0.83209	0.21231000000000044	-0.024850000000000705	catalytic activity	0008152 // metabolic process // inferred from electronic annotation /// 0055114 // oxidation reduction // inferred from electronic annotation	---	0003824 // catalytic activity // inferred from electronic annotation /// 0005488 // binding // inferred from electronic annotation /// 0008270 // zinc ion binding // inferred from electronic annotation /// 0016491 // oxidoreductase activity // inferred fr	-1.6683	1.027203	0.332892	0.113461	0.194748	0.824246	0.611936	0.544837	0.569694	7.606152	5.646954	4.654806	5.257141	0.000001607	0.00004665	0.000312	0.000097	5.793931	4.331188	3.505414	4.013141
Ag.UNKN.56.0_CDS_a_at	7363176	7364583	3L:7362969-7364716 (-) // 99.38 // /// 3L:7362969-7364725 (-) // 100.0 //	Up	AGAP010624	3L	ENSANGP00000016179	10.4052	10.82835	10.98719	10.90321	10.77617	0.858847	-0.15883999999999965	0.12703999999999915	binding	---	0005622 // intracellular // inferred from electronic annotation	0005525 // GTP binding // inferred from electronic annotation	-1.67235	0.215628	0.924312	0.549629	-0.01722	0.423149	0.581985	0.498008	0.370962	5.4643	7.515415	5.953935	4.79039	0.00006558	0.000001857	0.00002658	0.000239	4.183244	5.731133	4.575426	3.621311
Ag.3L.374.0_CDS_at	9851070	9851613	3L:9850991-9851807 (+) // 100.0 //	Up	AGAP010755	3L	ENSANGP00000020247	10.70398	11.12841	11.3464	11.26442	11.10218	0.81732	-0.21798999999999857	0.16223999999999883	unknown	---	0005634 // nucleus // inferred from electronic annotation	---	-1.63692	0.078123	0.958944	0.627719	-0.02786	0.424435	0.642418	0.560447	0.398206	4.525476	6.849693	5.532414	4.245812	0.000404	0.000005546	0.00005772	0.000707	3.394068	5.255986	4.23864	3.150875
Ag.3L.641.0_UTR_s_at	10579542	10585597	3L:10579542-10585597 (-) // 100.0 // /// 3L:10572361-10573285 (-) // 43.94 // /// 3L:10572361-10585597 (-) // 99.01 // /// 3L:10579542-10580307 (-) // 39.82 //	Up	AGAP010800	3L		10.56686	10.9644	10.86325	11.27403	10.99569	0.927692	0.10114999999999874	0.27834000000000003	binding	---	---	0003676 // nucleic acid binding // inferred from electronic annotation /// 0008270 // zinc ion binding // inferred from electronic annotation	-1.43513	0.123727	-0.2729	1.337871	0.24643	0.397539	0.296391	0.707171	0.428831	6.236062	4.649384	10.27026	6.726927	0.000016	0.000316	0.00000003557	0.000006831	4.794947	3.500761	7.448942	5.16554
Ag.3L.203.0_UTR_at	12373509	12376637	3L:12373509-12376637 (+) // 100.0 //	Up	---	3L		10.66526	11.30267	11.05268	11.55404	11.0644	0.895653	0.24999000000000038	0.4896400000000014	unknown	---	---	---	-1.40124	0.529705	-0.2276	1.291214	-0.19208	0.637411	0.387423	0.888787	0.39915	7.258618	4.411834	9.370413	4.545377	0.000002813	0.000506	0.000000118	0.000388	5.550877	3.295625	6.928125	3.41125
Ag.3L.2126.0_at	14393347	14393793	3L:14393347-14393793 (-) // 100.0 //	Up	---	3L		8.180737	9.221091	9.453749	9.485118	9.1354	0.761709	-0.2326580000000007	0.3497179999999993	unknown	---	---	---	-1.71728	0.236372	0.673273	0.73218	0.075455	1.040354	1.273012	1.304381	0.954663	4.779599	5.848476	5.548063	4.385917	0.000244	0.0000322	0.000056	0.000533	3.61212	4.49215	4.251329	3.273099
Ag.3L.528.0_CDS_a_at	17025705	17028014	3L:17023839-17028254 (+) // 100.0 //	Up	AGAP011110	3L	ENSANGP00000012561	7.071131	7.638898	7.553519	8.116322	7.635506	0.911469	0.08537900000000054	0.4808159999999999	transcription regulator activity	---	---	---	-1.43301	0.096504	-0.1335	1.382644	0.087366	0.567767	0.482387	1.04519	0.564374	6.378216	5.419077	10.87055	6.340109	0.00001248	0.0000714	0.0000000167	0.00001334	4.903751	4.146318	7.777326	4.874703
Ag.3L.951.0_CDS_at	17964556	17967603	3L:17964179-17967605 (+) // 100.0 //	Down	AGAP011144	3L	ENSANGP00000010079	8.488636	3.656677	4.509918	0.521457	2.812607	0.930871	-0.8532409999999997	-2.29115	unknown	---	---	---	1.539512	-0.11696	0.175542	-1.19177	-0.40632	-4.83196	-3.97872	-7.96718	-5.67603	-8.71243	-7.17396	-13.2999	-10.2344	0.0000002995	0.000003231	0.000000001064	0.00000003725	6.523667	5.490628	8.972991	7.428839
Ag.3L.1146.0_CDS_at	19245197	19247473	3L:19245197-19247473 (-) // 100.0 //	Down	---	3L		6.43262	3.663718	4.438628	3.620844	4.317535	0.816916	-0.7749099999999998	-0.6966910000000004	unknown	---	---	---	1.692514	-0.72571	-0.04894	-0.76316	-0.1547	-2.7689	-1.99399	-2.81178	-2.11509	-7.08878	-5.1049	-6.66455	-5.41492	0.000003719	0.00013	0.000007599	0.000072	5.429581	3.886609	5.119247	4.142915
Ag.3L.1152.0_CDS_at	19904502	19905219	3L:19904502-19905219 (-) // 100.0 //	Up	---	3L		6.092102	7.054154	7.360894	7.085128	7.120595	0.839344	-0.30674000000000046	-0.03546699999999969	unknown	---	---	---	-1.73353	0.227434	0.852667	0.290569	0.362862	0.962051	1.268792	0.993026	1.028493	5.950324	7.847522	5.686296	6.361265	0.00002676	0.0000011	0.00004337	0.00001286	4.572585	5.95875	4.362799	4.890841
Ag.3L.432.1_CDS_a_at	21636748	21647771	3L:21636765-21648300 (+) // 100.0 //	Up	AGAP011351	3L	ENSANGP00000029627	6.032732	6.918158	6.876145	7.064121	6.610281	0.824506	0.042012999999999856	0.45384000000000047	binding	---	---	---	-1.63783	0.534541	0.431463	0.892659	-0.22083	0.885427	0.843413	1.03139	0.57755	6.329651	6.029311	6.826163	4.12873	0.00001359	0.0000232	0.000005771	0.000895	4.866716	4.634541	5.238719	3.048194
Ag.3L.1932.0_CDS_at	26862873	26865561	3L:26862884-26865561 (-) // 100.0 //	Down	AGAP011530	3L	ENSANGP00000015940	7.908048	5.181574	4.783109	5.099099	4.530283	0.944855	0.39846500000000074	0.568816	binding	---	0005634 // nucleus // inferred from electronic annotation	0008270 // zinc ion binding // inferred from electronic annotation	1.756514	-0.23262	-0.52333	-0.29279	-0.70778	-2.72647	-3.12494	-2.80895	-3.37777	-11.14	-12.7681	-10.6256	-13.8011	0.00000001202	0.000000001872	0.00000002264	0.000000000636	7.920112	8.727768	7.64509	9.196571
Ag.3L.1976.0_CDS_at	32070346	32071268	3L:32070346-32071268 (-) // 100.0 //	Down	---	3L		9.030298	7.456609	8.185759	6.824336	7.490519	0.906341	-0.7291499999999989	-0.6661830000000002	unknown	---	---	---	1.466151	-0.40542	0.461747	-1.15738	-0.3651	-1.57369	-0.84454	-2.20596	-1.53978	-8.15347	-4.37566	-10.5815	-7.97779	0.0000006869	0.000544	0.00000002393	0.0000008987	6.163091	3.264179	7.621033	6.04637
Ag.3L.2357.0_at	33924383	33924823	3L:33924383-33924823 (-) // 100.0 // /// 3L:33924383-33924823 (-) // 94.76 //	Up	---	3L		10.50686	11.24975	10.78082	11.11326	10.9106	0.940306	0.4689300000000003	0.20265999999999984	unknown	---	---	---	-1.39891	1.164574	-0.45355	0.693603	-0.00572	0.742891	0.273963	0.606405	0.403742	11.15817	4.114898	8.432516	6.064181	0.00000001176	0.00092	0.0000004517	0.00002179	7.929644	3.036034	6.345116	4.661774
Ag.3L.2017.1_CDS_a_at	35231419	35233761	3L:35219173-35232580 (-) // 100.0 //	Down	AGAP011930	3L	ENSANGP00000008730	9.195089	7.969484	6.951936	7.961941	7.707794	0.844709	1.0175479999999997	0.25414700000000057	binding	---	---	0005515 // protein binding // inferred from electronic annotation	1.533789	0.01516	-1.24567	0.005814	-0.3091	-1.22561	-2.24315	-1.23315	-1.4873	-4.78454	-8.75686	-4.45688	-5.80613	0.000242	0.0000002808	0.000463	0.00003479	3.616328	6.551641	3.334714	4.458523
Ag.3L.324.0_CDS_a_at	35359199	35367736	3L:35359198-35368368 (+) // 100.0 //	Down	AGAP011936	3L	ENSANGP00000015354	7.047902	6.156462	6.004377	5.994858	5.927885	0.878295	0.15208500000000047	0.06697299999999995	catalytic activity	0005975 // carbohydrate metabolic process // inferred from electronic annotation /// 0006030 // chitin metabolic process // inferred from electronic annotation	0005576 // extracellular region // inferred from electronic annotation	0003824 // catalytic activity // inferred from electronic annotation /// 0008061 // chitin binding // inferred from electronic annotation /// 0016810 // hydrolase activity, acting on carbon-nitrogen (but not peptide) bonds // inferred from electronic anno	1.759913	-0.14959	-0.47536	-0.49575	-0.63921	-0.89144	-1.04353	-1.05304	-1.12002	-6.73054	-7.87882	-7.36091	-8.45635	0.000006789	0.000001047	0.000002382	0.000000436	5.168218	5.979887	5.623129	6.360476
Ag.3L.17.0_CDS_at	35698416	35700459	3L:35698398-35701209 (+) // 100.0 //	Down	AGAP011971	3L	ENSANGP00000015382	8.779118	6.903239	7.184529	4.673405	6.530123	0.931522	-0.28129000000000026	-1.8567179999999999	catalytic activity	0009166 // nucleotide catabolic process // inferred from electronic annotation	---	0016787 // hydrolase activity // inferred from electronic annotation	1.334747	0.060559	0.251625	-1.45405	-0.19288	-1.87588	-1.59459	-4.10571	-2.24899	-6.85317	-5.82553	-13.8868	-8.21628	0.000005514	0.00003358	0.000000000583	0.0000006246	5.258538	4.473946	9.234105	6.20442
Ag.3L.11.0_CDS_s_at	36520525	36520797	3L:8992159-8993131 (-) // 100.0 // /// UNKN:36519916-36520869 (-) // 95.27 // /// UNKN:36519916-36520869 (-) // 94.14 //	Up	AGAP010724 /// AGAP012885	3L	ENSANGP00000032063	9.724777	10.4461	10.24246	10.60896	10.20113	0.927942	0.20364000000000004	0.4078300000000006	binding	0006334 // nucleosome assembly // inferred from electronic annotation /// 0006334 // nucleosome assembly // inferred from sequence or structural similarity	0000786 // nucleosome // inferred from electronic annotation /// 0000786 // nucleosome // inferred from sequence or structural similarity /// 0005634 // nucleus // inferred from electronic annotation /// 0005694 // chromosome // inferred from electronic a	0003677 // DNA binding // inferred from electronic annotation /// 0003677 // DNA binding // inferred from sequence or structural similarity	-1.55793	0.603555	-0.00668	1.091561	-0.13051	0.721327	0.517681	0.884184	0.476356	9.365652	6.721526	10.62857	6.184975	0.0000001188	0.000006894	0.00000002256	0.00001756	6.925273	5.161541	7.646699	4.755549
Ag.3L.306.0_CDS_at	36837649	36838903	3L:36837578-36839002 (+) // 100.0 //	Up	AGAP012044	3L	ENSANGP00000008634	8.448509	9.046606	9.365793	9.031836	9.05981	0.852358	-0.31918699999999944	-0.027974000000000387	binding	---	---	0005488 // binding // inferred from electronic annotation	-1.62634	0.168321	1.126081	0.124003	0.20794	0.598098	0.917285	0.583328	0.611301	5.473864	8.395104	4.942666	5.594705	0.00006441	0.0000004776	0.000178	0.0000514	4.191038	6.320947	3.750398	4.289065
Ag.3L.153.0_CDS_a_at	36864110	36869012	3L:36859622-36870328 (+) // 100.0 // /// 3L:36859622-36870328 (+) // 98.84 //	Down	AGAP012048	3L	ENSANGP00000015768	10.56697	9.513493	9.531652	9.508838	9.476967	0.869618	-0.018158999999998926	0.03187100000000065	catalytic activity	0044262 // cellular carbohydrate metabolic process // inferred from electronic annotation	---	0004108 // citrate (Si)-synthase activity // inferred from electronic annotation /// 0016740 // transferase activity // inferred from electronic annotation /// 0046912 // transferase activity, transferring acyl groups, acyl groups converted into alkyl on 	1.787309	-0.43469	-0.39639	-0.44451	-0.51173	-1.05348	-1.03532	-1.05813	-1.09	-7.55598	-7.42573	-7.02638	-7.81795	0.000001741	0.000002145	0.000004125	0.000001152	5.759261	5.668604	5.384599	5.93873
Ag.3L.2909.0_at	36869211	36869377	3L:36869211-36869377 (-) // 100.0 //	Down	---	3L		11.24561	10.46235	10.44907	10.5141	10.30977	0.871649	0.013279999999999959	0.2043299999999988	unknown	---	---	---	1.751257	-0.36088	-0.3967	-0.22134	-0.77233	-0.78326	-0.79654	-0.73151	-0.93584	-7.01846	-7.1375	-6.06855	-8.38569	0.00000418	0.000003431	0.00002162	0.0000004843	5.378873	5.464546	4.665182	6.314853
Ag.3L.2049.0_CDS_at	37363403	37365080	3L:37363402-37369196 (-) // 100.0 //	Down	AGAP012069	3L	ENSANGP00000028181	6.784672	5.479844	5.82707	5.564498	5.771856	0.730001	-0.34722600000000003	-0.20735799999999927	catalytic activity	0006508 // proteolysis // inferred from electronic annotation	0005622 // intracellular // inferred from electronic annotation	0004198 // calcium-dependent cysteine-type endopeptidase activity // inferred from electronic annotation	1.720409	-0.7764	-0.11198	-0.61441	-0.21763	-1.30483	-0.9576	-1.22017	-1.01282	-5.60071	-4.11031	-4.84884	-4.34731	0.00005083	0.000929	0.000213	0.000576	4.293911	3.031998	3.670996	3.23949
Ag.3L.2922.0_at	37656104	37656565	3L:37656114-37656565 (-) // 97.83 // /// 3L:37656104-37656565 (-) // 100.0 //	Down	---	3L		11.00348	10.04514	10.34985	10.42477	10.47597	0.866406	-0.30471000000000004	-0.05119999999999969	unknown	---	---	---	1.567539	-1.19576	-0.31717	-0.10112	0.046504	-0.95835	-0.65364	-0.57871	-0.52751	-8.90846	-6.07601	-4.98045	-4.90359	0.0000002258	0.00002133	0.000165	0.000192	6.646367	4.670994	3.782248	3.717385
Ag.UNKN.867.0_CDS_s_at	39166945	39167398	3L:39166943-39167651 (-) // 98.22 // /// 3L:39166943-39167651 (-) // 98.4 //	Down	AGAP012232	3L	ENSANGP00000016702	6.030689	5.094496	5.070018	5.032183	5.113433	0.754559	0.024478000000000222	-0.08124999999999982	unknown	---	---	---	1.784331	-0.40639	-0.46367	-0.5522	-0.36207	-0.93619	-0.96067	-0.99851	-0.91726	-5.1723	-5.30755	-5.10736	-5.06768	0.000114	0.00008816	0.000129	0.000139	3.942788	4.054752	3.888663	3.855484
Ag.3L.2633.0_at	39774819	39774998	3L:39774819-39774998 (+) // 100.0 //	Up	---	3L		8.26756	9.425378	9.241081	9.469293	8.906157	0.901521	0.18429700000000082	0.5631360000000001	unknown	---	---	---	-1.60037	0.732326	0.361015	0.820803	-0.31377	1.157818	0.973521	1.201733	0.638597	9.327091	7.84244	8.962733	5.144376	0.0000001253	0.000001108	0.0000002089	0.00012	6.902132	5.955312	6.68	3.91954
Ag.3L.25.0_CDS_a_at	40213913	40214391	3L:40213527-40214477 (-) // 99.83 // /// 3L:40213813-40214477 (-) // 98.64 //	Down	AGAP012321	3L	ENSANGP00000020072	9.179284	8.090418	8.502901	7.776425	8.426278	0.862893	-0.41248299999999993	-0.6498530000000002	binding	0006810 // transport // inferred from electronic annotation	---	0005549 // odorant binding // inferred from electronic annotation	1.493333	-0.58011	0.20535	-1.17802	0.059443	-1.08887	-0.67638	-1.40286	-0.75301	-7.18924	-4.46582	-8.5753	-4.97173	0.000003151	0.000455	0.0000003659	0.000168	5.501528	3.342459	6.436691	3.774897
Ag.3L.368.0_CDS_a_at	41831176	41834030	3L:41831085-41834156 (-) // 100.0 //	Up	AGAP012417	3L	ENSANGP00000027128	9.70222	10.19477	10.10678	10.15384	10.03611	0.804328	0.08798999999999957	0.11772999999999989	unknown	---	---	---	-1.70693	0.791406	0.345097	0.583789	-0.01337	0.492552	0.404561	0.45162	0.333889	6.057414	4.9753	5.142031	4.106176	0.000022	0.000167	0.000121	0.000937	4.656495	3.777908	3.917586	3.028362
Ag.3L.156.0_UTR_s_at	.	.	---	Up	---	3L		10.93625	11.42077	10.08296	12.06261	10.23863	0.966686	1.3378099999999993	1.8239799999999988	unknown	---	---	---	-0.01455	0.573281	-1.04979	1.35199	-0.86093	0.484518	-0.85329	1.126365	-0.69762	4.30367	-7.57926	9.262641	-6.19654	0.000629	0.000001677	0.000000137	0.0000172	3.201439	5.775368	6.863299	4.764482
Ag.3L.1916.0_CDS_at	.	.	3L:25473135-25481026 (-) // 100.0 // /// 3L:25510073-25515373 (-) // 99.41 //	Down	AGAP011498	3L		6.193039	4.943822	5.313245	5.332212	5.308328	0.763996	-0.3694230000000003	0.023883999999999794	transporter activity	0006810 // transport // inferred from electronic annotation	0016020 // membrane // inferred from electronic annotation	---	1.67522	-1.02537	-0.22674	-0.18574	-0.23737	-1.24922	-0.87979	-0.86083	-0.88471	-6.50383	-4.58049	-4.14929	-4.60609	0.00001	0.000362	0.000859	0.000344	4.998888	3.441526	3.066257	3.463561
Ag.3L.324.0_UTR_a_at	.	.	3L:35359198-35368368 (+) // 100.0 //	Down	AGAP011936	3L		10.50087	9.515776	9.404361	9.347095	9.33593	0.905483	0.11141500000000093	0.011165000000000092	catalytic activity	0005975 // carbohydrate metabolic process // inferred from electronic annotation /// 0006030 // chitin metabolic process // inferred from electronic annotation	0005576 // extracellular region // inferred from electronic annotation	0003824 // catalytic activity // inferred from electronic annotation /// 0008061 // chitin binding // inferred from electronic annotation /// 0016810 // hydrolase activity, acting on carbon-nitrogen (but not peptide) bonds // inferred from electronic anno	1.770368	-0.21128	-0.43541	-0.55061	-0.57307	-0.9851	-1.09651	-1.15378	-1.16494	-7.9966	-8.90102	-8.67112	-9.45652	0.0000008731	0.0000002282	0.000000318	0.0000001048	6.058951	6.641741	6.497568	6.979529
Ag.UNKN.8.0_CDS_x_at	.	.	UNKN:37137565-37138241 (+) // 70.17 // /// 3L:38654985-38655749 (-) // 79.19 //	Up	AGAP012198	3L		6.319308	8.130478	8.702734	8.136437	7.993767	0.791506	-0.5722559999999994	0.14267000000000074	binding	---	---	---	-1.70506	0.303838	0.938569	0.310448	0.152203	1.81117	2.383427	1.81713	1.67446	5.33415	7.019527	4.954713	4.931519	0.00008382	0.000004172	0.000174	0.000182	4.076658	5.379644	3.76056	3.740988
Ag.UNKN.11.0_CDS_s_at	.	.	UNKN:37137561-37138186 (+) // 100.0 //	Up	AGAP012198 /// AGAP012895	3L		6.515781	8.455613	9.107352	8.508936	8.343931	0.7909	-0.651739000000001	0.16500500000000073	binding	0006334 // nucleosome assembly // inferred from electronic annotation	0000786 // nucleosome // inferred from electronic annotation /// 0005634 // nucleus // inferred from electronic annotation /// 0005694 // chromosome // inferred from electronic annotation	0003677 // DNA binding // inferred from electronic annotation	-1.70489	0.274828	0.939968	0.329247	0.160849	1.939832	2.591571	1.993155	1.82815	5.255086	7.020673	4.999002	4.952532	0.0000974	0.000004164	0.000159	0.000174	4.011441	5.380472	3.797854	3.758721
Ag.UNKN.8.0_CDS_s_at	.	.	UNKN:37137565-37138241 (+) // 70.17 // /// 3L:38654985-38655749 (-) // 79.19 //	Up	AGAP012198 /// AGAP012895	3L		6.609556	8.458858	9.065831	8.449329	8.294287	0.790772	-0.606973	0.1550419999999999	binding	0006334 // nucleosome assembly // inferred from electronic annotation	0000786 // nucleosome // inferred from electronic annotation /// 0005634 // nucleus // inferred from electronic annotation /// 0005694 // chromosome // inferred from electronic annotation	0003677 // DNA binding // inferred from electronic annotation	-1.69503	0.306625	0.963603	0.29631	0.128495	1.849303	2.456275	1.839773	1.684732	5.303657	7.044408	4.88493	4.83168	0.00008881	0.000004	0.000199	0.000221	4.051547	5.397618	3.701592	3.656428
Ag.3R.449.0_CDS_at	1325615	1326422	3R:1325433-1326459 (+) // 100.0 //	Up	AGAP007812	3R	ENSANGP00000010077	9.241888	10.3457	9.677404	10.45166	9.821623	0.935394	0.6682960000000016	0.6300369999999997	peroxisome fission	0016559 // peroxisome fission // inferred from electronic annotation	0005778 // peroxisomal membrane // inferred from electronic annotation	---	-1.33724	0.879843	-0.46247	1.09267	-0.1728	1.103812	0.435516	1.209772	0.579735	10.98341	4.333572	11.14479	5.768615	0.00000001454	0.000592	0.00000001195	0.00003727	7.837469	3.227522	7.922627	4.428648
Ag.3R.609.1_CDS_a_at	2538876	2550738	3R:2538558-2540007 (-) // 91.46 // /// 3R:2538558-2545043 (-) // 100.0 //	Up	AGAP007892	3R	ENSANGP00000007267	7.408699	7.903437	7.938372	8.104997	7.968841	0.76343	-0.03493499999999994	0.13615599999999883	transcription regulator activity	0006355 // regulation of transcription, DNA-dependent // inferred from electronic annotation	0005634 // nucleus // inferred from electronic annotation	---	-1.71363	0.144882	0.276117	0.902053	0.390576	0.494738	0.529673	0.696298	0.560142	4.37732	4.686415	5.703673	4.955998	0.000542	0.000293	0.000042	0.000173	3.265621	3.532512	4.376733	3.761644
Ag.3R.541.0_CDS_a_at	3384349	3385919	3R:3384225-3386165 (-) // 100.0 //	Up	AGAP007965	3R	ENSANGP00000009954	8.786787	9.328703	9.564832	9.447284	9.409018	0.85934	-0.23612899999999826	0.03826600000000013	binding	---	---	0003676 // nucleic acid binding // inferred from electronic annotation	-1.71696	0.070515	0.849373	0.461648	0.335428	0.541916	0.778045	0.660497	0.622231	5.531731	7.942073	6.242041	6.351563	0.0000578	0.0000009496	0.00001587	0.000013	4.238086	6.022439	4.799548	4.883443
Ag.3R.180.0_CDS_at	3648647	3649500	3R:3648525-3649937 (+) // 100.0 //	Down	AGAP007980	3R	ENSANGP00000012363	11.49062	7.131172	7.010643	5.486334	6.083647	0.981386	0.12052900000000033	-0.5973129999999998	binding	---	---	---	1.713648	-0.13087	-0.18187	-0.82682	-0.57409	-4.35944	-4.47997	-6.00428	-5.40697	-18.4703	-18.9809	-23.5521	-22.9085	0.0000000000102	0.00000000000687	0.000000000000299	0.000000000000449	10.99231	11.1629	12.52366	12.34796
Ag.3R.2184.0_CDS_at	7908909	7909193	3R:7908908-7909188 (-) // 100.0 //	Down	AGAP008253	3R	ENSANGP00000006757	8.206376	7.327478	7.456426	6.933579	7.439136	0.887318	-0.12894800000000028	-0.5055570000000005	unknown	---	---	---	1.590465	-0.31455	-0.03505	-1.16833	-0.07253	-0.8789	-0.74995	-1.2728	-0.76724	-7.22109	-6.16164	-9.68166	-6.3037	0.000003	0.0000183	0.00000007721	0.00001423	5.524217	4.737503	7.112333	4.846869
Ag.3R.34.0_CDS_x_at	8545842	8555880	3R:8544010-8545228 (+) // 99.42 //	Down	AGAP008278	3R	ENSANGP00000018280	11.39569	9.160459	10.22054	7.848449	9.693253	0.930047	-1.0600810000000003	-1.8448040000000008	binding	0006810 // transport // inferred from electronic annotation	---	0005549 // odorant binding // inferred from electronic annotation	1.322655	-0.38428	0.425249	-1.3862	0.022585	-2.23523	-1.17515	-3.54724	-1.70243	-8.98824	-4.72548	-13.206	-6.84578	0.0000002015	0.000272	0.000000001174	0.000005583	6.695759	3.565932	8.930298	5.25312
Ag.3R.780.0_CDS_at	10088633	10089362	3R:10088541-10089363 (+) // 100.0 //	Down	AGAP008369	3R	ENSANGP00000025714	6.82674	4.457037	4.950522	3.469181	4.234777	0.9552	-0.4934850000000006	-0.7655960000000004	transporter activity	0006869 // lipid transport // inferred from electronic annotation	---	0005319 // lipid transporter activity // inferred from electronic annotation	1.619938	-0.26265	0.129392	-1.04745	-0.43923	-2.3697	-1.87622	-3.35756	-2.59196	-12.078	-9.56275	-15.8435	-13.2108	0.000000004014	0.00000009068	0.0000000000912	0.000000001168	8.396408	7.042482	10.04014	8.932499
Ag.3R.231.0_CDS_at	10877893	10878411	3R:10873286-10874017 (+) // 100.0 //	Down	AGAP008447	3R	ENSANGP00000019347	11.38565	7.160908	7.085674	4.271759	5.920211	0.965997	0.07523400000000002	-1.6484519999999998	structural molecule activity	---	---	---	1.602759	-0.00149	-0.03006	-1.09858	-0.47262	-4.22474	-4.29997	-7.11389	-5.46544	-12.111	-12.3267	-18.8806	-15.6677	0.000000003867	0.000000003037	0.00000000000742	0.000000000107	8.412643	8.517622	11.12971	9.97146
Ag.3R.576.0_CDS_at	10882271	10882859	3R:10882221-10882897 (+) // 99.33 //	Down	AGAP008450	3R	ENSANGP00000019303	10.6781	7.425856	8.624468	5.876315	7.269542	0.94998	-1.1986120000000007	-1.3932270000000004	binding	---	---	---	1.503412	-0.30533	0.361281	-1.16711	-0.39226	-3.25225	-2.05364	-4.80179	-3.40856	-11.1924	-7.06744	-15.2992	-11.7303	0.00000001128	0.000003853	0.00000000015	0.000000005977	7.947544	5.414222	9.825163	8.223502
Ag.3R.283.0_CDS_at	11221160	11223012	3R:11220702-11223124 (+) // 99.95 //	Up	AGAP008487	3R	ENSANGP00000019358	6.116671	8.159832	7.405285	8.632874	7.37043	0.866003	0.7545469999999996	1.2624439999999995	catalytic activity	0006685 // sphingomyelin catabolic process // inferred from electronic annotation	---	0004767 // sphingomyelin phosphodiesterase activity // inferred from electronic annotation /// 0016787 // hydrolase activity // inferred from electronic annotation	-1.48663	0.651876	-0.13788	1.146992	-0.17436	2.043161	1.288614	2.516203	1.253759	7.691074	4.850732	8.769136	4.719527	0.000001405	0.000213	0.0000002758	0.000275	5.852288	3.672603	6.559356	3.560847
Ag.3R.302.0_CDS_a_at	13262401	13264364	3R:13260495-13264533 (+) // 100.0 //	Up	AGAP008578	3R	ENSANGP00000013029	9.244134	9.720465	10.00963	9.917673	9.728851	0.820315	-0.2891649999999988	0.18882200000000005	binding	---	---	0000166 // nucleotide binding // inferred from electronic annotation /// 0003676 // nucleic acid binding // inferred from electronic annotation /// 0004386 // helicase activity // inferred from electronic annotation /// 0005524 // ATP binding // inferred 	-1.62414	-0.01247	0.965925	0.654786	0.015902	0.476331	0.765498	0.67354	0.484717	4.421668	7.105929	5.788513	4.499512	0.000496	0.000003615	0.000036	0.000425	3.304165	5.441906	4.444506	3.371625
Ag.3R.184.2_CDS_a_at	20239532	20240493	3R:20239216-20240188 (-) // 100.0 //	Down	AGAP008878	3R	ENSANGP00000013935	9.743491	8.406117	9.027412	7.818046	8.86762	0.911433	-0.6212949999999999	-1.0495740000000007	catalytic activity	---	---	---	1.352312	-0.51034	0.354981	-1.32938	0.132428	-1.33737	-0.71608	-1.92545	-0.87587	-8.40526	-4.50048	-11.2036	-5.50476	0.0000004704	0.000424	0.00000001113	0.00006079	6.327517	3.372466	7.953394	4.216185
Ag.3R.586.1_a_at	23784089	23786267	3R:23784498-23786404 (+) // 100.0 //	Up	AGAP009024	3R	ENSANGP00000017197	9.761775	10.48626	10.36054	10.78956	10.21362	0.91706	0.1257199999999994	0.5759399999999992	transcription regulator activity	---	---	---	-1.48153	0.433181	0.100942	1.23477	-0.28736	0.72448	0.598769	1.027781	0.451844	8.152801	6.738134	10.70799	5.08474	0.0000006876	0.000006701	0.00000002043	0.000135	6.162647	5.173834	7.689824	3.869759
Ag.3R.209.0_CDS_at	25436126	25439559	3R:25435147-25439464 (+) // 100.0 //	Up	AGAP009075	3R	ENSANGP00000023803	10.49498	10.98642	11.02194	11.01981	10.89285	0.854017	-0.035519999999999996	0.1269600000000004	catalytic activity	---	---	---	-1.7389	0.462334	0.621449	0.611895	0.04322	0.491433	0.526956	0.524823	0.397864	6.285317	6.739649	6.214446	5.088596	0.0000147	0.000006684	0.00001666	0.000134	4.832784	5.174955	4.778296	3.872983
Ag.3R.979.0_CDS_at	25597268	25599732	3R:25597267-25599732 (+) // 100.0 //	Down	AGAP009100	3R	ENSANGP00000015956	9.087882	7.599578	7.601605	6.974128	7.950704	0.859664	-0.002027000000000001	-0.9765759999999997	catalytic activity	0018149 // peptide cross-linking // inferred from electronic annotation	---	0003810 // protein-glutamine gamma-glutamyltransferase activity // inferred from electronic annotation	1.596221	-0.31178	-0.30919	-1.11361	0.138359	-1.4883	-1.48628	-2.11375	-1.13718	-6.63014	-6.6211	-8.7179	-5.06593	0.000008061	0.000008187	0.0000002971	0.00014	5.093603	5.086861	6.527115	3.854017
Ag.3R.420.0_CDS_at	26744916	26763095	3R:20451455-20453439 (+) // 100.0 // /// 3R:20451455-20453439 (+) // 94.91 //	Down	AGAP008892	3R	ENSANGP00000031532	11.73094	10.16737	10.43755	10.67062	10.62332	0.914392	-0.27017999999999986	0.0472999999999999	transcription regulator activity	---	---	---	1.687442	-0.93792	-0.48427	-0.09291	-0.17234	-1.56357	-1.29339	-1.06032	-1.10762	-11.2261	-9.28631	-7.04813	-7.95248	0.00000001084	0.0000001326	0.000003978	0.0000009345	7.965158	6.877581	5.400304	6.029422
Ag.3R.404.1_CDS_s_at	28117585	28118794	3R:28117492-28120385 (-) // 100.0 //	Up	AGAP009173	3R	ENSANGP00000016841	8.929674	10.14142	9.525703	10.54256	9.488121	0.946491	0.6157170000000001	1.0544390000000003	catalytic activity	0005975 // carbohydrate metabolic process // inferred from electronic annotation	---	0016787 // hydrolase activity // inferred from electronic annotation /// 0042578 // phosphoric ester hydrolase activity // inferred from electronic annotation	-1.2702	0.663852	-0.31888	1.304098	-0.37887	1.211746	0.596029	1.612882	0.558448	10.97044	5.3961	13.5189	5.055856	0.00000001477	0.00007456	0.000000000848	0.000143	7.830584	4.127512	9.071572	3.845579
Ag.3R.467.0_CDS_s_at	31895576	31898526	3R:31923567-31928332 (-) // 100.0 // /// 3R:31895532-31898987 (-) // 45.97 //	Down	AGAP009384	3R	ENSANGP00000030744	8.989288	7.439392	7.712001	7.132407	7.76998	0.857619	-0.2726090000000001	-0.6375730000000006	binding	---	---	---	1.670395	-0.52237	-0.13669	-0.95668	-0.05466	-1.5499	-1.27729	-1.85688	-1.21931	-7.44824	-6.13818	-8.26156	-5.85956	0.000002069	0.000019	0.0000005833	0.00003156	5.684343	4.719328	6.234083	4.500931
Ag.3R.467.0_UTR_at	31923805	31928003	3R:31923567-31928332 (-) // 100.0 // /// 3R:31895532-31898987 (-) // 45.97 //	Down	AGAP009384	3R	ENSANGP00000015121	11.0564	9.694244	9.981486	9.406992	9.973031	0.930932	-0.2872420000000009	-0.566039	unknown	---	---	---	1.655845	-0.52557	-0.06557	-0.98559	-0.07911	-1.36215	-1.07491	-1.64941	-1.08337	-10.723	-8.46183	-12.0211	-8.52839	0.00000002005	0.0000004325	0.000000004281	0.000000392	7.697967	6.364003	8.36843	6.406721
Ag.3R.2458.0_CDS_at	34412358	34413303	3R:34412363-34413303 (-) // 100.0 //	Down	AGAP009480	3R	ENSANGP00000011186	6.9439	5.801276	5.762959	5.279339	5.873355	0.808137	0.03831699999999927	-0.5940159999999999	chitin binding	0006030 // chitin metabolic process // inferred from electronic annotation	0005576 // extracellular region // inferred from electronic annotation	0008061 // chitin binding // inferred from electronic annotation	1.6527	-0.21381	-0.2764	-1.06641	-0.09607	-1.14262	-1.18094	-1.66456	-1.07055	-5.34892	-5.52829	-7.21421	-5.0115	0.00008151	0.00005817	0.000003025	0.000155	4.088799	4.235292	5.519323	3.808357
Ag.3R.1544.0_CDS_s_at	42404090	42404836	3R:42388174-42389980 (-) // 100.0 // /// 3R:42404230-42404819 (-) // 69.05 //	Down	AGAP009758	3R	ENSANGP00000022326	9.284102	4.726089	6.051706	2.989939	5.744178	0.97	-1.3256170000000003	-2.7542389999999997	structural molecule activity	---	---	---	1.52943	-0.44826	0.126915	-1.20156	-0.00652	-4.55801	-3.2324	-6.29416	-3.53992	-16.0084	-11.3527	-20.4662	-12.4327	0.0000000000787	0.000000009314	0.00000000000231	0.0000000027	10.10399	8.030868	11.63593	8.568675
Ag.3R.1545.0_CDS_a_at	42430986	42431603	3R:42430510-42431600 (-) // 99.9 //	Down	AGAP009759	3R	ENSANGP00000014460	7.709941	4.539111	4.796046	4.312528	4.498135	0.958452	-0.25693499999999947	-0.18560700000000008	cuticular protein	---	---	---	1.775787	-0.44209	-0.26237	-0.60058	-0.47075	-3.17083	-2.9139	-3.39741	-3.21181	-14.4928	-13.3184	-14.3765	-14.6801	0.000000000321	0.000000001044	0.000000000359	0.000000000268	9.493866	8.981395	9.444771	9.572231
Ag.3R.2526.0_CDS_at	42482116	42483186	3R:42482115-42482892 (-) // 100.0 //	Up	AGAP009766	3R	ENSANGP00000026031	6.616674	8.535086	8.651843	8.66302	8.851698	0.744641	-0.11675699999999978	-0.18867800000000123	enzyme regulator activity	---	---	0004867 // serine-type endopeptidase inhibitor activity // inferred from electronic annotation	-1.77543	0.292588	0.41845	0.430499	0.63389	1.918412	2.035169	2.046346	2.235024	4.836328	5.130673	4.776168	5.634508	0.000219	0.000124	0.000246	0.00004773	3.660376	3.908118	3.609195	4.321168
Ag.3R.408.0_CDS_at	43300711	43304243	3R:43300527-43321185 (-) // 100.0 //	Down	AGAP009790	3R	ENSANGP00000015840	8.775232	7.643535	7.466385	6.972816	7.572357	0.910801	0.17715000000000014	-0.5995410000000003	structural molecule activity	0006030 // chitin metabolic process // inferred from electronic annotation	0005576 // extracellular region // inferred from electronic annotation	0008061 // chitin binding // inferred from electronic annotation	1.642912	-0.06415	-0.33137	-1.07587	-0.17152	-1.1317	-1.30885	-1.80242	-1.20287	-7.47676	-8.64714	-11.0247	-7.94701	0.000001976	0.0000003293	0.00000001383	0.0000009424	5.704238	6.482375	7.859329	6.025754
Ag.3R.646.0_CDS_a_at	45725343	45734159	3R:45724344-45750578 (-) // 100.0 //	Up	AGAP009940	3R	ENSANGP00000013307	10.23408	11.31314	11.37263	11.51	11.07726	0.865033	-0.05948999999999849	0.432739999999999	intracellular signaling cascade	0007242 // intracellular signaling cascade // inferred from electronic annotation	---	---	-1.70249	0.415579	0.53235	0.801982	-0.04742	1.079062	1.138551	1.275917	0.843184	7.212088	7.609697	7.895212	5.635561	0.000003035	0.000001598	0.000001021	0.00004764	5.517812	5.796375	5.990935	4.322016
Ag.UNKN.268.0_CDS_at	49237424	49238561	3L:38067039-38067868 (+) // 95.53 // /// 3L:38067039-38067868 (+) // 95.65 //	Down	---	3R	ENSANGP00000030675	10.52593	9.800451	9.916638	9.689613	9.950616	0.792678	-0.11618700000000004	-0.26100300000000054	structural molecule activity	---	---	---	1.696281	-0.54413	-0.18533	-0.88642	-0.0804	-0.72548	-0.6093	-0.83632	-0.57532	-5.81526	-4.88394	-6.20642	-4.61158	0.00003422	0.000199	0.000017	0.00034	4.465781	3.70075	4.772106	3.468281
Ag.3R.648.0_CDS_at	49426594	49429977	3R:49425842-49430314 (-) // 100.0 //	Down	AGAP010145	3R	ENSANGP00000012608	8.217282	7.388501	7.136716	7.289516	7.4321	0.775486	0.2517849999999999	-0.14258400000000027	catalytic activity	---	---	---	1.722515	-0.24804	-0.8467	-0.48339	-0.14438	-0.82878	-1.08057	-0.92777	-0.78518	-4.90043	-6.38919	-5.07878	-4.64264	0.000193	0.00001224	0.000137	0.00032	3.714712	4.912101	3.864776	3.494972
Ag.3R.2715.0_at	.	.	3R:8601802-8602333 (+) // 100.0 //	Up	---	3R		10.40446	11.00056	10.73066	11.18355	11.04875	0.934927	0.2698999999999998	0.13480000000000025	unknown	---	---	---	-1.51533	0.410094	-0.46169	1.001175	0.565756	0.596102	0.326201	0.779098	0.644294	8.427404	4.611677	10.19745	9.108718	0.0000004552	0.00034	0.00000003907	0.0000001699	6.341818	3.468367	7.408112	6.769762
Ag.3R.291.0_CDS_at	.	.	3R:19096323-19097907 (+) // 100.0 //	Up	---	3R		11.2922	11.72876	11.97975	11.90458	11.86146	0.854152	-0.2509899999999998	0.04312000000000005	unknown	---	---	---	-1.68647	-0.08994	0.827984	0.553055	0.395365	0.436555	0.687551	0.612374	0.569256	4.775681	7.521445	6.202118	6.227358	0.000246	0.000001839	0.000017	0.00001628	3.608781	5.73532	4.768788	4.788246
Ag.3R.3667.0_at	.	.	3R:34976791-34977142 (-) // 98.87 //	Up	---	3R		8.524188	9.145975	9.078185	9.242735	9.082176	0.732445	0.06779000000000046	0.16055899999999923	unknown	---	---	---	-1.73835	0.46545	0.225181	0.808395	0.239326	0.621787	0.553997	0.718546	0.557987	4.835277	4.308113	5.173222	4.339146	0.000219	0.000623	0.000114	0.000586	3.659484	3.205316	3.94355	3.232381
Ag.3R.1223.0_CDS_at	.	.	3R:10089400-10090172 (+) // 100.0 //	Down	AGAP008369	3R		8.019714	5.570537	6.285386	3.533498	5.602029	0.943593	-0.7148490000000001	-2.068531	lipid transporter activity	0006869 // lipid transport // inferred from electronic annotation	---	0005319 // lipid transporter activity // inferred from electronic annotation	1.37578	-0.14375	0.29976	-1.40758	-0.12421	-2.44918	-1.73433	-4.48622	-2.41768	-9.01568	-6.38425	-15.2892	-8.89975	0.0000001938	0.00001235	0.000000000151	0.0000002286	6.712674	4.90834	9.821162	6.640955
Ag.3R.191.3_a_at	.	.	3R:10093783-10094207 (+) // 100.0 //	Down	AGAP008370	3R		10.79351	9.849195	10.16282	10.07588	10.1001	0.757541	-0.31362500000000004	-0.024219999999999686	catalytic activity	0006508 // proteolysis // inferred from electronic annotation	---	0004180 // carboxypeptidase activity // inferred from electronic annotation /// 0008270 // zinc ion binding // inferred from electronic annotation	1.685763	-0.97979	-0.0945	-0.33993	-0.27155	-0.94431	-0.63069	-0.71763	-0.69341	-6.23161	-4.16195	-4.38443	-4.57588	0.00001616	0.000837	0.000535	0.000365	4.791518	3.077371	3.271802	3.437552
Ag.UNKN.160.0_CDS_s_at	.	.	3R:10868353-10868923 (-) // 97.47 //	Down	AGAP008445	3R		7.958863	5.387233	4.992182	4.279799	4.45761	0.915138	0.3950510000000005	-0.17781100000000016	unknown	---	---	---	1.709543	-0.01875	-0.28425	-0.76302	-0.64352	-2.57163	-2.96668	-3.67906	-3.50125	-7.85752	-9.06459	-10.4074	-10.698	0.000001083	0.0000001808	0.00000002984	0.00000002068	5.965509	6.742736	7.525238	7.68439
Ag.3R.287.0_CDS_s_at	.	.	3R:10871404-10872221 (-) // 100.0 //	Down	AGAP008446	3R		11.21412	6.785176	7.131058	4.612591	6.217876	0.973736	-0.34588200000000047	-1.6052850000000003	cuticular protein	---	---	---	1.643706	-0.16633	-0.02497	-1.05423	-0.39818	-4.42894	-4.08306	-6.60153	-4.99624	-15.5037	-14.2929	-21.3947	-17.4896	0.000000000124	0.00000000039	0.00000000000121	0.0000000000223	9.906735	9.409262	11.91561	10.65216
Ag.3R.184.0_CDS_a_at	.	.	3R:20239216-20243761 (-) // 100.0 //	Down	AGAP008878	3R		9.604138	8.086358	8.671861	7.740208	8.602613	0.856122	-0.5855029999999992	-0.8624049999999999	neuronal development and vascular remodelling	---	---	---	1.503774	-0.64315	0.185054	-1.13278	0.087102	-1.51778	-0.93228	-1.86393	-1.00153	-7.34141	-4.50937	-8.34693	-4.84432	0.000002458	0.000417	0.0000005132	0.000215	5.609404	3.380148	6.28972	3.667159
Ag.3R.586.0_CDS_a_at	.	.	3R:23784088-23786406 (+) // 100.0 //	Up	AGAP009024	3R		8.257277	9.210744	9.068865	9.402701	8.912856	0.890202	0.14187899999999942	0.48984500000000075	transcription initiation factor	---	---	---	-1.62959	0.54895	0.224775	0.987543	-0.13168	0.953467	0.811587	1.145423	0.655579	8.211424	6.989533	9.132832	5.64596	0.0000006292	0.000004386	0.0000001643	0.00004673	6.20123	5.357927	6.784491	4.330388
Ag.3R.404.2_s_at	.	.	3R:28117492-28120385 (-) // 100.0 //	Up	AGAP009173	3R		9.853865	10.96265	10.39033	11.43368	10.4297	0.949654	0.5723199999999995	1.0039800000000003	catalytic activity	0005975 // carbohydrate metabolic process // inferred from electronic annotation	---	0016787 // hydrolase activity // inferred from electronic annotation /// 0042578 // phosphoric ester hydrolase activity // inferred from electronic annotation	-1.26032	0.577953	-0.37091	1.358895	-0.30563	1.10878	0.536461	1.579817	0.575835	10.70693	5.180336	14.12385	5.560551	0.00000002045	0.000112	0.00000000046	0.00005477	7.68925	3.949465	9.336869	4.261445
Ag.3R.347.0_CDS_a_at	.	.	3R:31447235-31460578 (+) // 100.0 //	Down	AGAP009330	3R		11.72037	10.95671	11.10307	10.7989	11.09767	0.919452	-0.14636000000000138	-0.2987700000000011	binding	0001539 // ciliary or flagellar motility // inferred from electronic annotation	0009288 // flagellin-based flagellum // inferred from electronic annotation	0005509 // calcium ion binding // inferred from electronic annotation	1.67181	-0.51049	-0.09223	-0.96144	-0.10766	-0.76366	-0.6173	-0.92146	-0.6227	-9.09126	-7.34884	-10.1561	-7.41312	0.0000001741	0.000002429	0.00000004122	0.000002189	6.759084	5.614637	7.384846	5.659774
Ag.3R.347.0_CDS_s_at	.	.	3R:31447235-31460578 (+) // 100.0 //	Down	AGAP009330	3R		10.30312	9.508682	9.612969	9.267632	9.55033	0.872718	-0.10428699999999935	-0.2826979999999999	binding	0001539 // ciliary or flagellar motility // inferred from electronic annotation	0009288 // flagellin-based flagellum // inferred from electronic annotation	0005509 // calcium ion binding // inferred from electronic annotation	1.684446	-0.35992	-0.09156	-0.98022	-0.25275	-0.79444	-0.69016	-1.03549	-0.75279	-7.05137	-6.12574	-8.50912	-6.68171	0.000003957	0.00001951	0.0000004033	0.000007379	5.402641	4.709669	6.394379	5.132004
Ag.3R.347.3_a_at	.	.	3R:31272707-31460578 (+) // 100.0 //	Down	AGAP009330	3R		11.72211	10.9521	11.15524	10.86888	11.12932	0.949439	-0.20313999999999943	-0.2604399999999991	binding	0001539 // ciliary or flagellar motility // inferred from electronic annotation	0009288 // flagellin-based flagellum // inferred from electronic annotation	0005509 // calcium ion binding // inferred from electronic annotation	1.669163	-0.64007	-0.03085	-0.88964	-0.1086	-0.77001	-0.56687	-0.85323	-0.59279	-11.2642	-8.29246	-11.5556	-8.67172	0.00000001035	0.0000005568	0.000000007327	0.0000003177	7.98499	6.254268	8.135044	6.497948
Ag.3R.195.0_UTR_a_at	.	.	3R:33543197-33553806 (+) // 98.81 // /// 3R:33543197-33553807 (+) // 99.8 //	Up	AGAP009447	3R		12.00071	12.65675	12.30181	12.85714	12.37777	0.957305	0.3549400000000009	0.4793699999999994	catalytic activity	0006730 // one-carbon compound metabolic process // inferred from electronic annotation	---	0000166 // nucleotide binding // inferred from electronic annotation /// 0000287 // magnesium ion binding // inferred from electronic annotation /// 0004478 // methionine adenosyltransferase activity // inferred from electronic annotation /// 0005524 // A	-1.32564	0.659347	-0.4146	1.265664	-0.18477	0.656045	0.3011	0.856435	0.377062	10.36909	4.759031	12.53222	5.959646	0.00000003133	0.000254	0.00000000242	0.00002631	7.504016	3.594584	8.616243	4.579917
Ag.3R.2476.0_CDS_a_at	.	.	3R:36043410-36044140 (-) // 100.0 //	Up	AGAP009551	3R		6.893551	8.096207	8.145514	8.892652	8.437307	0.86991	-0.049307000000000656	0.45534499999999944	sulfotransferase family protein	---	---	0008146 // sulfotransferase activity // inferred from electronic annotation	-1.61761	0.004263	0.070756	1.078327	0.46426	1.202656	1.251962	1.999101	1.543756	5.852547	6.09249	9.006681	7.512457	0.000032	0.00002071	0.0000001963	0.000001866	4.495377	4.683829	6.707131	5.729078
Ag.3R.407.1_at	.	.	3R:42403273-42404362 (-) // 99.33 // /// 3R:42403290-42404620 (-) // 93.61 //	Down	AGAP009758	3R		11.89801	8.028167	9.347645	6.661528	8.738027	0.968027	-1.3194780000000002	-2.076499000000001	cuticular protein	---	---	---	1.531305	-0.46844	0.213402	-1.17465	-0.10162	-3.86984	-2.55036	-5.23648	-3.15998	-15.5754	-10.2648	-19.5125	-12.7184	0.000000000116	0.00000003582	0.00000000000461	0.000000001975	9.935107	7.445867	11.33602	8.704409
Ag.3R.2559.0_CDS_at	.	.	3R:44655530-44658644 (-) // 100.0 //	Down	AGAP009876	3R		6.870657	5.980377	6.097929	5.595687	6.043503	0.873397	-0.11755199999999988	-0.44781600000000044	structural molecule activity	---	---	0042302 // structural constituent of cuticle // inferred from electronic annotation	1.620072	-0.29529	-0.04239	-1.12292	-0.15948	-0.89028	-0.77273	-1.27497	-0.82715	-6.82571	-5.92445	-9.04999	-6.34173	0.000005776	0.000028	0.0000001846	0.00001331	5.238387	4.552209	6.733773	4.875939
Ag.3R.1330.1_a_at	.	.	3R:44722310-44724856 (+) // 100.0 //	Down	AGAP009879	3R		11.95974	10.98402	11.14676	10.47285	11.22118	0.906342	-0.16274000000000122	-0.748330000000001	structural molecule activity	---	---	0042302 // structural constituent of cuticle // inferred from electronic annotation	1.499503	-0.32291	-0.01896	-1.27767	0.120047	-0.97572	-0.81298	-1.48689	-0.73856	-7.76363	-6.46879	-10.9534	-5.87659	0.000001254	0.00001065	0.00000001508	0.00003059	5.901829	4.972447	7.821508	4.514416
Ag.3R.2566.0_CDS_at	.	.	3R:45286194-45290318 (-) // 100.0 //	Down	AGAP009917	3R		5.495748	3.763478	3.947285	2.586985	3.609505	0.856436	-0.18380699999999983	-1.02252	Cell envelope biogenesis	---	---	---	1.544588	-0.11201	0.063771	-1.2371	-0.25925	-1.73227	-1.54846	-2.90876	-1.88624	-5.78382	-5.17012	-8.99155	-6.29792	0.00003624	0.000115	0.0000002005	0.00001437	4.440771	3.940968	6.6978	4.842441
Ag.3R.646.0_UTR_a_at	.	.	3R:45724344-45750578 (-) // 100.0 //	Up	AGAP009940	3R		8.888781	9.683613	9.835692	9.672368	9.568005	0.739721	-0.15207900000000052	0.1043630000000011	intracellular signaling cascade	0007242 // intracellular signaling cascade // inferred from electronic annotation	---	---	-1.72854	0.415125	0.825282	0.384798	0.10333	0.794832	0.946911	0.783588	0.679224	4.814415	5.735582	4.394225	4.114158	0.000228	0.0000396	0.000524	0.000922	3.641755	4.402272	3.280322	3.035383
Ag.X.157.0_CDS_at	752726	753781	X:752562-754633 (-) // 100.0 //	Down	AGAP000047	X	ENSANGP00000017440	6.253795	4.380223	4.481604	4.780528	4.547307	0.878538	-0.10138099999999994	0.23322100000000034	structural molecule activity	---	---	0042302 // structural constituent of cuticle // inferred from electronic annotation	1.756492	-0.65425	-0.5238	-0.13918	-0.43926	-1.87357	-1.77219	-1.47327	-1.70649	-8.71552	-8.24391	-6.34499	-7.93827	0.0000002981	0.0000005991	0.00001323	0.0000009552	6.525617	6.222535	4.878431	6.01989
Ag.X.18.0_CDS_at	2455198	2455537	X:2454887-2455537 (-) // 100.0 //	Down	AGAP000151	X	ENSANGP00000019451	10.52223	8.910998	9.167345	7.236647	8.702593	0.92907	-0.2563469999999999	-1.4659460000000006	unknown	---	---	---	1.375575	0.002586	0.221029	-1.42419	-0.175	-1.61123	-1.35489	-3.28559	-1.81964	-7.18208	-6.03942	-13.5591	-8.11105	0.000003188	0.00002278	0.000000000814	0.0000007327	5.496425	4.64244	9.089521	6.135058
Ag.X.56.0_CDS_at	2456118	2456418	X:2456117-2456349 (-) // 100.0 //	Down	AGAP000152	X	ENSANGP00000019369	9.861515	7.617552	8.24305	6.284433	7.909902	0.942661	-0.6254980000000003	-1.6254689999999998	unknown	---	---	---	1.459934	-0.28429	0.20191	-1.32051	-0.05704	-2.24396	-1.61846	-3.57708	-1.95161	-10.1094	-7.29141	-14.9198	-8.79229	0.00000004381	0.000002666	0.000000000213	0.0000002668	7.358412	5.574104	9.671277	6.573882
Ag.X.136.0_CDS_at	2832638	2836037	X:2832352-2836376 (-) // 99.81 //	Up	AGAP000162	X	ENSANGP00000019297	9.287553	10.99872	10.1234	11.05801	9.997556	0.932073	0.8753200000000003	1.060454	catalytic activity	0006535 // cysteine biosynthetic process from serine // inferred from electronic annotation /// 0008152 // metabolic process // inferred from electronic annotation /// 0019343 // cysteine biosynthetic process via cystathionine // inferred from electronic 	0005737 // cytoplasm // inferred from electronic annotation	0003824 // catalytic activity // inferred from electronic annotation /// 0004122 // cystathionine beta-synthase activity // inferred from electronic annotation /// 0030170 // pyridoxal phosphate binding // inferred from electronic annotation	-1.35266	0.949316	-0.22822	1.029079	-0.39752	1.711164	0.835851	1.770455	0.710003	11.62104	5.676518	11.13179	4.821846	0.000000006788	0.00004416	0.00000001214	0.000225	8.168287	4.354951	7.9158	3.648072
Ag.X.725.0_CDS_at	5901119	5902033	X:5901220-5902028 (+) // 100.0 //	Up	AGAP000342	X	ENSANGP00000028838	6.862157	7.754538	7.56455	7.983092	7.625432	0.869698	0.1899880000000005	0.3576600000000001	signal transducer activity	---	0005576 // extracellular region // inferred from electronic annotation	0008083 // growth factor activity // inferred from electronic annotation	-1.65371	0.467226	0.015678	1.010432	0.160376	0.892382	0.702394	1.120935	0.763275	7.313619	5.75655	8.505283	6.25551	0.000002572	0.0000381	0.0000004056	0.00001549	5.589801	4.419022	6.391917	4.809904
Ag.X.239.0_CDS_a_at	5962513	5967245	X:5961990-5967875 (+) // 100.0 //	Down	AGAP000344	X	ENSANGP00000015578	8.509483	6.548058	6.959935	6.438126	6.660509	0.952832	-0.4118769999999996	-0.22238300000000066	structural molecule activity	---	---	0042302 // structural constituent of cuticle // inferred from electronic annotation	1.741728	-0.55684	-0.07417	-0.68567	-0.42506	-1.96143	-1.54955	-2.07136	-1.84897	-13.7566	-10.8678	-13.4499	-12.9679	0.000000000665	0.00000001675	0.000000000911	0.00000000151	9.177001	7.77588	9.040679	8.820907
Ag.X.295.0_CDS_at	6358270	6363034	X:6358176-6368050 (-) // 100.0 // /// X:6358191-6368050 (-) // 99.05 //	Down	AGAP000359	X	ENSANGP00000021951	8.225276	6.80611	6.952013	6.401318	6.692774	0.82216	-0.14590299999999967	-0.29145600000000016	catalytic activity	0005975 // carbohydrate metabolic process // inferred from electronic annotation /// 0006030 // chitin metabolic process // inferred from electronic annotation	0005576 // extracellular region // inferred from electronic annotation	0003824 // catalytic activity // inferred from electronic annotation /// 0008061 // chitin binding // inferred from electronic annotation /// 0016810 // hydrolase activity, acting on carbon-nitrogen (but not peptide) bonds // inferred from electronic anno	1.71397	-0.29665	-0.08994	-0.87015	-0.45722	-1.41917	-1.27326	-1.82396	-1.5325	-5.97806	-5.36346	-7.11326	-6.45547	0.00002545	0.0000793	0.000003571	0.000011	4.594385	4.100745	5.447166	4.962377
Ag.X.299.0_CDS_at	9886411	9888765	X:9885450-9889019 (-) // 100.0 // /// X:9885450-9889019 (-) // 99.75 //	Up	---	X	ENSANGP00000017311	8.223449	8.527035	8.55441	8.625865	8.583846	0.837438	-0.027375000000001037	0.042018999999999807	signal transducer activity	---	---	---	-1.74159	0.15027	0.320865	0.766155	0.504304	0.303585	0.330961	0.402416	0.360397	4.661241	5.08156	5.720348	5.533525	0.000308	0.000136	0.00004073	0.0000576	3.510935	3.867098	4.390087	4.239541
Ag.X.399.1_at	12532274	12536552	X:12519284-12536726 (+) // 100.0 // /// X:12519284-12536726 (+) // 94.9 //	Down	---	X	ENSANGP00000024734	7.840985	6.024648	6.479861	5.088173	6.324455	0.854994	-0.45521299999999965	-1.236282	unknown	---	---	---	1.500925	-0.32952	0.129233	-1.27326	-0.02738	-1.81634	-1.36112	-2.75281	-1.51653	-6.36854	-4.77244	-8.93607	-5.31734	0.00001269	0.000248	0.000000217	0.00008653	4.896382	3.606022	6.663491	4.06282
Ag.X.5.0_CDS_a_at	13067687	13073294	X:13067562-13073532 (-) // 100.0 //	Up	AGAP000719	X	ENSANGP00000011950	9.452208	10.80815	10.29312	10.67587	10.16973	0.893765	0.5150299999999994	0.5061400000000003	catalytic activity	0006730 // one-carbon compound metabolic process // inferred from electronic annotation /// 0008152 // metabolic process // inferred from electronic annotation	---	0003824 // catalytic activity // inferred from electronic annotation /// 0004013 // adenosylhomocysteinase activity // inferred from electronic annotation /// 0005488 // binding // inferred from electronic annotation /// 0016787 // hydrolase activity // i	-1.55488	0.992609	0.025	0.744098	-0.20683	1.35594	0.840914	1.223666	0.717519	9.836212	6.100132	8.218203	5.205	0.00000006278	0.00002043	0.0000006228	0.000107	7.20218	4.689775	6.20568	3.969948
Ag.X.328.0_CDS_at	13870406	13870815	X:13870124-13871264 (+) // 100.0 //	Up	AGAP000772	X	ENSANGP00000028695	7.507799	8.376189	8.7631	8.612511	8.565064	0.859009	-0.38691099999999956	0.04744700000000002	unknown	---	---	---	-1.71884	0.022573	0.798457	0.496477	0.40133	0.86839	1.255301	1.104712	1.057265	5.711375	8.256078	6.726694	6.953602	0.00004141	0.0000005882	0.000006833	0.000004658	4.382903	6.230497	5.165368	5.331844
Ag.X.80.0_CDS_a_at	15282116	15282875	X:15281267-15283382 (-) // 99.43 // /// X:15281257-15283382 (-) // 100.0 //	Down	AGAP000820	X	ENSANGP00000023009	9.364414	6.946224	7.071797	6.833694	6.758609	0.972835	-0.12557300000000016	0.07508500000000051	structural molecule activity	---	---	0042302 // structural constituent of cuticle // inferred from electronic annotation	1.77861	-0.40524	-0.29183	-0.50686	-0.57467	-2.41819	-2.29262	-2.53072	-2.60581	-17.2507	-16.3549	-16.7143	-18.5891	0.0000000000271	0.000000000058	0.0000000000426	0.00000000000928	10.56669	10.23622	10.37071	11.0324
Ag.X.77.0_CDS_a_at	15729502	15730392	X:15728706-15731353 (-) // 100.0 //	Up	AGAP000852	X	ENSANGP00000010395	11.3089	11.79947	11.85383	11.92188	11.69917	0.859295	-0.05436000000000085	0.2227099999999993	binding	0006464 // protein modification process // inferred from electronic annotation	---	---	-1.68453	0.342164	0.566725	0.847866	-0.07222	0.490571	0.544926	0.612978	0.390267	6.05848	6.729767	7.008638	4.819743	0.000022	0.000006798	0.000004248	0.000226	4.657327	5.167643	5.371766	3.646284
Ag.X.627.0_CDS_at	17036510	17042078	X:17036377-17042085 (-) // 100.0 // /// X:17036378-17042085 (-) // 99.83 //	Down	AGAP000902	X	ENSANGP00000013105	8.424383	7.5732	7.554329	6.974733	7.596579	0.843653	0.01887099999999986	-0.6218460000000006	unknown	---	---	---	1.546023	-0.09945	-0.13593	-1.25638	-0.05426	-0.85118	-0.87005	-1.44965	-0.8278	-5.34007	-5.45847	-8.42004	-5.1934	0.00008288	0.0000663	0.0000004602	0.00011	4.081529	4.178488	6.337064	3.960318
Ag.X.460.0_CDS_at	18943257	18946249	X:18943235-18946469 (-) // 100.0 //	Down	AGAP000989	X	ENSANGP00000018544	5.98405	4.699957	4.107768	4.750963	4.625886	0.828996	0.5921890000000003	0.12507699999999922	chitin metabolic process	0006030 // chitin metabolic process // inferred from electronic annotation	0005576 // extracellular region // inferred from electronic annotation	0008061 // chitin binding // inferred from electronic annotation	1.66113	-0.19317	-1.04832	-0.11951	-0.30013	-1.28409	-1.87628	-1.23309	-1.35816	-5.50294	-8.04075	-4.89237	-5.82037	0.000061	0.0000008158	0.000196	0.0000339	4.214705	6.088391	3.707887	4.469846
Ag.X.1107.0_CDS_a_at	19579781	19580329	X:19579784-19580988 (-) // 100.0 //	Up	AGAP001020	X	ENSANGP00000016101	6.868021	7.553151	7.338328	7.74903	7.33035	0.883645	0.214823	0.41868000000000016	unknown	---	---	---	-1.52116	0.564247	-0.08963	1.160466	-0.11392	0.68513	0.470307	0.881009	0.462329	7.391007	5.07355	8.799084	4.987485	0.000002268	0.000138	0.0000002642	0.000163	5.644275	3.860396	6.57814	3.788166
Ag.UNKN.203.0_CDS_a_at	20969946	20975092	X:20969558-21004978 (-) // 100.0 //	Down	AGAP001052	X	ENSANGP00000000895	8.736124	7.375447	7.439425	6.959981	7.59698	0.860811	-0.06397799999999965	-0.6369990000000003	catalytic activity	0006511 // ubiquitin-dependent protein catabolic process // inferred from electronic annotation	---	0004221 // ubiquitin thiolesterase activity // inferred from electronic annotation	1.673442	-0.36958	-0.27352	-0.99339	-0.03695	-1.36068	-1.2967	-1.77614	-1.13914	-7.02744	-6.69702	-8.49272	-5.8833	0.000004118	0.000007188	0.0000004132	0.00003022	5.385365	5.143369	6.383854	4.519719
Ag.UNKN.203.2_a_at	20996492	21004978	X:21004806-21116709 (-) // 100.0 //	Down	AGAP001052	X	ENSANGP00000031854	6.967384	5.554241	5.842574	5.008657	5.869933	0.804236	-0.28833299999999973	-0.8612759999999993	catalytic activity	0006511 // ubiquitin-dependent protein catabolic process // inferred from electronic annotation	---	0004221 // ubiquitin thiolesterase activity // inferred from electronic annotation	1.565014	-0.41169	-0.00837	-1.17485	0.029899	-1.41314	-1.12481	-1.95873	-1.09745	-5.67182	-4.51456	-7.27842	-4.40475	0.00004455	0.000412	0.000002723	0.000513	4.351179	3.384636	5.564909	3.289474
Ag.X.1127.0_at	.	.	X:11602328-11603175 (+) // 86.26 // /// X:11602219-11603250 (+) // 100.0 //	Up	---	X		3.579996	8.271891	6.395837	8.010259	7.153782	0.943043	1.8760539999999999	0.8564769999999999	unknown	---	---	---	-1.64546	0.843078	-0.15197	0.70431	0.250042	4.691896	2.815842	4.430263	3.573786	13.98333	8.392094	12.22414	10.65101	0.00000000053	0.0000004797	0.000000003405	0.00000002193	9.276133	6.319	8.467887	7.658908
Ag.X.1161.0_a_at	.	.	X:15360378-15364739 (+) // 99.78 // /// X:15360378-15364739 (+) // 98.57 //	Up	---	X		8.074247	8.624467	8.612706	8.609495	8.602179	0.81871	0.01176099999999991	0.00731600000000121	unknown	---	---	---	-1.78786	0.497877	0.449016	0.435678	0.405285	0.55022	0.538458	0.535248	0.527931	5.988417	5.860405	5.393329	5.745834	0.000025	0.00003151	0.000075	0.00003886	4.602511	4.501603	4.125241	4.410465
Ag.X.1345.0_at	.	.	X:1086869-1087788 (+) // 98.2 //	Down	---	X		8.388464	6.891825	7.53365	6.504799	7.317207	0.880203	-0.6418249999999999	-0.8124079999999996	unknown	---	---	---	1.487944	-0.6104	0.289465	-1.15302	-0.01399	-1.49664	-0.85481	-1.88367	-1.07126	-7.95277	-4.54227	-9.26684	-5.6924	0.0000009341	0.00039	0.0000001362	0.00004289	6.029616	3.40857	6.865836	4.367691
Ag.X.1748.0_at	.	.	X:10003398-10003863 (-) // 98.5 // /// X:10003392-10003863 (-) // 100.0 //	Up	---	X		9.009438	9.528205	9.60769	9.648177	9.389801	0.847211	-0.07948500000000003	0.25837600000000016	unknown	---	---	---	-1.65333	0.354266	0.661869	0.81855	-0.18135	0.518767	0.598252	0.638739	0.380363	5.865379	6.764066	6.686111	4.300525	0.00003122	0.000006412	0.000007324	0.000633	4.505542	5.192996	5.135273	3.198693
Ag.X.218.0_UTR_at	.	.	X:9590149-9594799 (-) // 100.0 //	Up	AGAP000535	X		6.053406	7.221138	6.932704	7.176721	7.495394	0.831488	0.28843399999999964	-0.31867300000000043	transferase activity	---	---	0016747 // transferase activity, transferring groups other than amino-acyl groups // inferred from electronic annotation	-1.66813	0.443524	-0.07806	0.363203	0.939471	1.167732	0.879298	1.123316	1.441988	6.288351	4.735104	5.600437	7.765244	0.00001462	0.000267	0.00005085	0.00000125	4.83511	3.574159	4.293694	5.902928
Ag.X.358.0_CDS_a_at	.	.	X:11415590-11488724 (-) // 100.0 //	Up	AGAP000645	X		6.006085	6.561128	6.610997	6.653122	6.548968	0.791293	-0.04986900000000016	0.10415399999999941	IPP transferase	---	---	---	-1.76694	0.319824	0.507315	0.66569	0.274107	0.555043	0.604912	0.647037	0.542883	5.209963	5.678068	5.622947	5.095824	0.000106	0.000044	0.00004877	0.000132	3.974066	4.356195	4.311853	3.879025
Ag.X.399.0_CDS_a_at	.	.	X:12519284-12529829 (+) // 100.0 //	Down	AGAP000697	X		7.477223	6.364239	6.702228	5.872017	6.592658	0.848875	-0.33798899999999943	-0.7206410000000005	unknown	---	---	---	1.4985	-0.40637	0.172099	-1.2488	-0.01543	-1.11298	-0.775	-1.60521	-0.88456	-6.34167	-4.41584	-8.46783	-5.04016	0.00001331	0.000502	0.0000004287	0.000147	4.875895	3.299108	6.367863	3.832421
Ag.X.80.0_UTR_a_at	.	.	X:15281267-15283382 (-) // 99.43 // /// X:15281257-15283382 (-) // 100.0 //	Down	AGAP000820	X		11.41816	8.253683	9.069291	6.99151	8.340025	0.969321	-0.8156079999999992	-1.3485150000000008	structural molecule activity	---	---	0042302 // structural constituent of cuticle // inferred from electronic annotation	1.591391	-0.3428	0.155713	-1.11427	-0.29003	-3.16448	-2.34887	-4.42665	-3.07814	-15.2214	-11.2983	-19.7131	-14.8061	0.000000000161	0.000000009938	0.00000000000398	0.000000000237	9.793908	8.002714	11.40023	9.624485
Ag.UNKN.203.2_at	.	.	X:21004806-21116709 (-) // 100.0 //	Down	AGAP001052	X		7.982095	6.602137	6.732545	5.985286	6.977436	0.819698	-0.13040800000000008	-0.9921499999999996	catalytic activity	0006511 // ubiquitin-dependent protein catabolic process // inferred from electronic annotation	---	0004221 // ubiquitin thiolesterase activity // inferred from electronic annotation	1.546705	-0.34851	-0.16941	-1.19569	0.166916	-1.37996	-1.24955	-1.99681	-1.00466	-5.7254	-5.18434	-7.67014	-4.1683	0.00004035	0.000111	0.000001452	0.000826	4.394129	3.952795	5.837937	3.082947
Ag.UNKN.1116.0_CDS_at	.	.	---	Up	---			2.32139	3.576606	3.427407	3.290339	3.197215	0.761759	0.14919899999999986	0.09312399999999998	unknown	---	---	---	-1.71116	0.842181	0.538683	0.259863	0.070431	1.255216	1.106017	0.968949	0.875825	6.135208	5.405957	4.384687	4.280833	0.00001919	0.00007318	0.000535	0.000658	4.71702	4.135583	3.272029	3.181495
Ag.UNKN.1322.0_CDS_at	.	.	3R:10877896-10878102 (+) // 64.67 //	Down	---			12.39628	9.635686	10.90175	7.003999	8.858312	0.976862	-1.266064	-1.8543129999999994	unknown	---	---	---	1.291474	-0.06049	0.559547	-1.34933	-0.4412	-2.76059	-1.49453	-5.39228	-3.53796	-12.5877	-6.81474	-22.7638	-16.1324	0.000000002277	0.000005884	0.000000000000492	0.0000000000705	8.642646	5.230329	12.30781	10.15159
Ag.UNKN.186.1_s_at	.	.	3R:10871414-10872214 (-) // 98.96 //	Down	---			11.66221	8.288704	8.665359	6.552478	7.804179	0.971239	-0.3766550000000013	-1.2517010000000006	unknown	---	---	---	1.622205	-0.16176	0.037426	-1.0799	-0.41798	-3.37351	-2.99685	-5.10973	-3.85803	-14.5328	-12.9102	-20.3794	-16.6201	0.000000000309	0.000000001606	0.00000000000246	0.0000000000462	9.51068	8.794139	11.60919	10.33571
Ag.UNKN.200.0_CDS_at	.	.	3L:26293958-26294714 (-) // 57.06 //	Down	---			9.053467	8.124287	7.706585	7.56114	7.291914	0.934349	0.41770200000000113	0.26922599999999974	unknown	---	---	---	1.608328	0.257115	-0.35031	-0.56181	-0.95332	-0.92918	-1.34688	-1.49233	-1.76155	-6.73205	-9.75836	-10.0101	-12.7627	0.000006771	0.00000006965	0.00000004989	0.000000001883	5.16933	7.157057	7.302004	8.725256
Ag.UNKN.2212.0_at	.	.	UNKN:38031005-38031771 (+) // 100.0 // /// UNKN:40136888-40137654 (+) // 99.87 // /// UNKN:40143591-40234570 (+) // 99.61 // /// UNKN:40143663-40144325 (+) // 86.29 // /// UNKN:40911339-40912002 (+) // 86.29 // /// UNKN:41008450-41009115 (+) // 86.68 // /// UNKN:33428281-33428928 (+) // 84.46 // /// UNKN:42275204-42275782 (+) // 75.33 // /// X:24366401-24390771 (+) // 98.04 // /// X:23384499-23401565 (+) // 98.04 // /// UNKN:38848271-38849037 (-) // 99.87 // /// UNKN:33568642-33569406 (-) // 99.74 // /// UNKN:35109969-35110736 (-) // 99.87 // /// UNKN:33473069-33473834 (-) // 99.74 // /// UNKN:35493163-35493929 (-) // 99.61 // /// UNKN:41733390-41734149 (-) // 98.83 // /// UNKN:36333619-36478237 (-) // 99.61 // /// UNKN:7339333-7370414 (-) // 99.09 // /// UNKN:42269014-42269761 (-) // 97.52 //	Up	---			8.679768	9.997988	9.516437	9.973014	9.529552	0.79061	0.4815509999999996	0.44346199999999847	unknown	---	---	---	-1.61162	0.859891	-0.04296	0.813067	-0.01837	1.31822	0.836669	1.293246	0.849784	6.577968	4.175011	5.974636	4.240454	0.000008819	0.000815	0.0000256	0.000714	5.054599	3.088837	4.591695	3.146187
Ag.UNKN.2219.0_s_at	.	.	UNKN:35492929-35493500 (+) // 100.0 // /// UNKN:33568408-33568978 (+) // 99.82 // /// UNKN:41733156-41733728 (+) // 99.82 // /// UNKN:33472835-33473405 (+) // 99.65 // /// UNKN:42268781-42269350 (+) // 99.65 // /// UNKN:34865006-35110306 (+) // 100.0 // /// UNKN:36333385-36477810 (+) // 100.0 // /// UNKN:7339099-7339670 (+) // 98.77 // /// UNKN:9429327-9429897 (+) // 97.72 // /// UNKN:36550690-36551207 (+) // 90.54 // /// UNKN:29814292-29814864 (-) // 100.0 // /// UNKN:40234233-40234804 (-) // 99.65 // /// UNKN:40137317-40137842 (-) // 91.94 // /// UNKN:26189683-26190211 (-) // 89.67 // /// UNKN:38031434-38839628 (-) // 100.0 // /// X:24390439-24391010 (-) // 97.9 // /// X:23398556-23401799 (-) // 98.25 //	Up	---			9.597478	10.82491	10.33246	10.68623	10.41599	0.83159	0.49244999999999983	0.27023999999999937	unknown	---	---	---	-1.62507	0.952217	-0.08178	0.661044	0.093594	1.227427	0.734987	1.088757	0.81851	7.686581	4.602744	6.312406	5.125797	0.000001415	0.000346	0.000014	0.000125	5.849211	3.460683	4.853531	3.904051
Ag.UNKN.2445.0_at	.	.	2R:61060740-61061138 (+) // 92.77 // /// 3L:211136-588077 (+) // 93.25 // /// 3R:35438865-35439276 (+) // 95.42 // /// UNKN:10971618-10972033 (+) // 100.0 // /// UNKN:3283326-3297370 (+) // 97.11 // /// UNKN:27479682-27480090 (+) // 94.7 // /// UNKN:3808849-4072024 (+) // 95.18 // /// UNKN:6989120-6989537 (+) // 92.53 // /// X:24190124-24190534 (+) // 95.42 // /// 3R:19008889-19009299 (-) // 93.01 // /// UNKN:26159798-26160213 (-) // 98.31 // /// UNKN:3298868-3299279 (-) // 95.42 // /// UNKN:4336933-4337339 (-) // 95.18 // /// UNKN:7870449-7870867 (-) // 95.42 // /// UNKN:24042751-24043166 (-) // 94.7 // /// UNKN:77671-78079 (-) // 93.98 // /// X:24178426-24178835 (-) // 94.7 // /// 2L:35632196-35632610 (-) // 95.9 // /// 2L:5275407-5275816 (-) // 95.66 // /// 2L:532063-532475 (-) // 94.7 //	Up	---			7.21575	8.129099	7.826879	7.904295	8.06967	0.787494	0.30222000000000016	-0.16537500000000005	unknown	---	---	---	-1.68554	0.824263	-0.00621	0.206524	0.660958	0.913349	0.611129	0.688545	0.85392	6.347291	4.247027	4.430073	5.93429	0.00001318	0.000705	0.000488	0.00002754	4.880185	3.151938	3.311461	4.559964
Ag.UNKN.298.0_CDS_at	.	.	3R:11139719-11144497 (-) // 96.75 //	Up	---			8.084375	8.514834	8.539187	8.7159	8.605769	0.817432	-0.024352999999999625	0.11013099999999909	unknown	---	---	---	-1.69269	0.094761	0.195889	0.92968	0.472365	0.430459	0.454813	0.631526	0.521394	4.753826	5.022781	6.456978	5.758081	0.000257	0.000152	0.00001088	0.000038	3.590143	3.817836	4.963515	4.420244
Ag.UNKN.310.0_CDS_a_at	.	.	---	Up	---			7.693343	8.261872	8.141011	8.376075	8.31511	0.865295	0.12086099999999966	0.06096499999999949	unknown	---	---	---	-1.697	0.381672	-0.06022	0.799224	0.576323	0.568529	0.447668	0.682732	0.621767	6.50732	5.123957	7.234803	7.116681	0.00001	0.000125	0.000002924	0.000003551	5.00152	3.902516	5.53397	5.449623
Ag.UNKN.377.0_CDS_at	.	.	3R:10873456-10875679 (-) // 85.92 //	Down	---			5.385471	3.946559	4.01662	3.285847	3.249236	0.845661	-0.07006099999999948	0.03661100000000017	unknown	---	---	---	1.628387	-0.03489	0.046091	-0.79863	-0.84095	-1.43891	-1.36885	-2.09962	-2.13623	-5.37915	-5.11724	-7.26688	-7.98599	0.000077	0.000127	0.000002775	0.0000008874	4.113622	3.896914	5.556734	6.051858
Ag.UNKN.134.0_CDS_at	.	.	UNKN:4564588-4589150 (-) // 100.0 //	Down	AGAP012466			9.436089	8.453876	8.56579	8.222061	8.514978	0.830749	-0.11191400000000051	-0.2929169999999992	structural molecule activity	---	---	0042302 // structural constituent of cuticle // inferred from electronic annotation	1.715813	-0.39733	-0.15656	-0.89606	-0.26587	-0.98221	-0.8703	-1.21403	-0.92111	-6.3482	-5.62488	-7.26441	-5.95329	0.00001316	0.00004859	0.000002786	0.00002661	4.880876	4.313411	5.554981	4.574916
